# Structure–Activity Relationships of Inactive‐Conformation Binding EGFR Inhibitors: Linking the ATP and Allosteric Pockets

**DOI:** 10.1002/ardp.70027

**Published:** 2025-07-16

**Authors:** Surbhi P. Chitnis, Florian Wittlinger, Mareike Möllers, Tyler J. Hartman, Marcel Günther, Michael J. Eck, Stefan A. Laufer, David E. Heppner

**Affiliations:** ^1^ Department of Chemistry The State University of New York at Buffalo Buffalo New York USA; ^2^ Department of Pharmaceutical and Medicinal Chemistry, Institute of Pharmaceutical Sciences Eberhard Karls Universität Tübingen Tübingen Germany; ^3^ Department of Medicinal Chemistry, Faculty of Medicine, Institute for Biomedical Engineering Eberhard Karls Universität Tübingen Tübingen Germany; ^4^ Department of Cancer Biology Dana‐Farber Cancer Institute Boston Massachusetts USA; ^5^ Department of Biological Chemistry and Molecular Pharmacology Harvard Medical School Boston Massachusetts USA; ^6^ Cluster of Excellence iFIT (EXC 2180) “Image‐Guided and Functionally Instructed Tumor Therapies” Eberhard Karls Universität Tübingen Tübingen Germany; ^7^ Tübingen Center for Academic Drug Discovery & Development (TüCAD2) Tübingen Germany; ^8^ Department of Structural Biology University at Buffalo, The State University of New York Buffalo New York USA; ^9^ Department of Pharmaceutical Sciences University at Buffalo, The State University of New York Buffalo New York USA; ^10^ Department of Pharmacology and Therapeutics Roswell Park Comprehensive Cancer Center Buffalo New York USA

**Keywords:** allosteric inhibitors, epidermal growth factor receptor (EGFR), kinase inhibitors, non‐small cell lung cancer (NSCLC), type I½ inhibitors

## Abstract

The epidermal growth factor receptor (EGFR) tyrosine kinase is an important therapeutic target in non‐small cell lung cancer (NSCLC). However, the continual emergence of resistance mutations in the treatment of EGFR mutation‐positive NSCLC with currently approved tyrosine kinase inhibitors warrants the development of next‐generation inhibitors. Since research for ATP‐competitive EGFR tyrosine kinase inhibitors (TKIs) that extend into the back pocket has been neglected in the recent past, we survey the extent to which such binding functional groups can be incorporated into an ATP‐site imidazole scaffold. We find that *meta*‐substituted amide linkers derivatized with fluorine in 2,6‐positions and/or a hydroxy group in 3‐position of the back pocket phenyl exhibit the highest potency. Structural insights into how the back pocket groups are bound through points of connection provide new directions for the discovery and optimization of inactive conformation targeting agents in EGFR and other kinases.

## Graphical abstract

EGFR inhibitors can be made especially effective through binding small‐molecule inhibitors that target the allosteric pocket. Structural and functional analysis affords new insights into the compositions and their molecular connections that afford effective inhibitors.

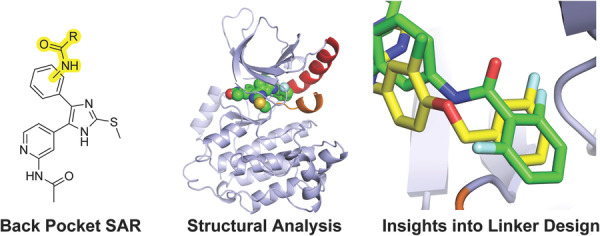

## 1 Introduction

**Figure 1:**
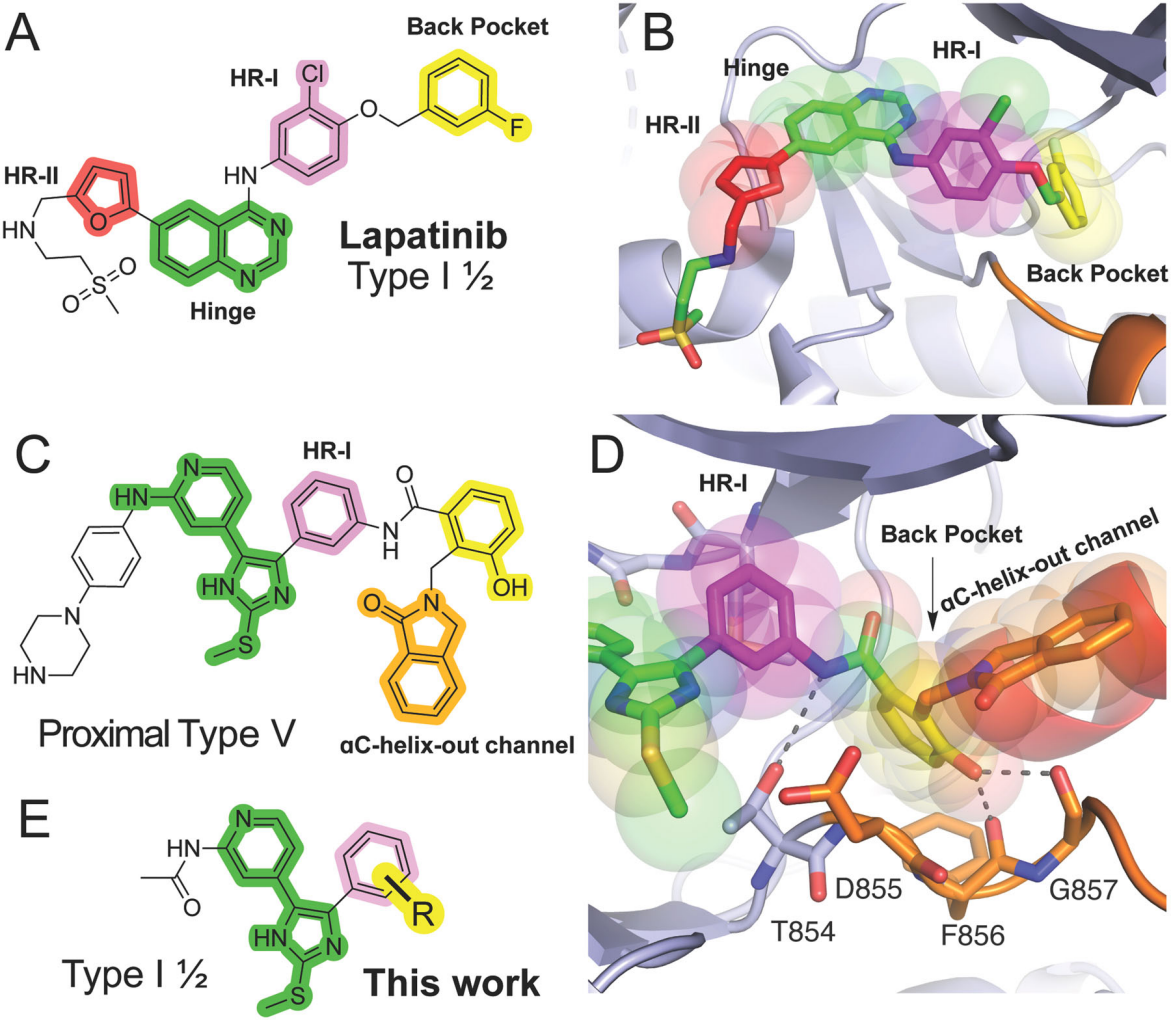
Functional groups of TKIs and their binding sites in inhibitors that bind to the back pocket of the allosteric site. (A) Chemical structure and (B) X‐ray cocrystal structure (PDB ID 1XKK) of the reversible‐binding Type I½ TKI Lapatinib anchored in the kinase domain at the hydrophobic region II (HR‐II, red), hinge (green), hydrophobic region I (HR‐I, magenta), and back pocket (yellow). (C) Chemical structure and (D) X‐ray cocrystal structure (PDB ID 8TO3) of the reversible binding proximal Type V ATP‐allosteric bivalent inhibitor (AABI) LN5461. LN5461 binds a distinct group into the ɑC‐helix‐out channel of the allosteric pocket (orange). Colors indicate identical binding sites in A–B. (E) Chemical structures of various substitutions of the back pocket.

Protein kinases are intensively pursued targets in oncology and increasingly in other human diseases and comprise approximately 80 FDA‐approved drugs [1]. With a vast number of over 500 human kinases and decades‐long emphasis on drug development, diverse kinase inhibitors have been disclosed and often differentiated by “Types” on the basis of structural details of their binding modes [2, 3]. The majority of kinase inhibitors bind the ATP‐substrate (orthosteric) site and are classified as either Type I or II based on whether the conserved DFG motif is positioned in the “in” or “out” conformation, respectively [4]. Also, inhibitors are known where ATP‐site binding is achieved with compounds that anchor groups into a pocket only accessible in the inactive conformation, that is, when the αC‐helix is positioned in the “out” conformation (Supporting Information S2: Figure S1). These inhibitors are categorized as “Type I½” that are unique as they bind into the hydrophobic “back pocket,” which is exemplified by the example of lapatinib (yellow in Figure 1A,B) [5]. These inhibitors also target the “hinge” region (green in Figure 1A,B), which is a flexible region that connects the N‐ and C‐lobe of the kinase. In addition, hydrophobic regions I (HR‐I) (magenta in Figure 1A,B) and II (HR‐II) (red in Figure 1A,B) are located “east” and “west” of the hinge region and offer opportunities to improve TKI selectivity or physiochemical properties. More recently, our groups have defined “proximal Type V” ATP‐allosteric bivalent inhibitors (AABIs), which bind via an additional isoindolinone group within the “αC‐helix‐out channel” (orange in Figure 1C,D) [6]. The back pocket and αC‐helix‐out channel comprise a site that is utilized in allosteric‐only Type III inhibitor binding (that are ATP‐noncompetitive) similar to Type I½ and proximal Type V inhibitors that exclusively bind to the inactive conformation of the kinase domain [7, 8, 9, 10]. The chemical groups that bind the back pocket and αC‐helix‐out channel represent important binding sites where structure–activity relationships (SARs) can be carried out to engender important selectivity properties in small molecules as these regions of the kinase domain are typically of lower homology between kinases and access to the allosteric site is made available in certain regulatory or disease‐related conditions [9].

A kinase target in oncology where optimization of back‐pocket binding groups has been impactful is the epidermal growth factor receptor tyrosine kinase (EGFR) that is often observed mutated in diverse human cancers [11]. Specifically, EGFR driver mutations, such as L858R (LR) and deletions in exon 19 (ex19del), are commonly found in non‐small cell lung cancer (NSCLC), and tumors harboring these mutations are especially responsive to treatment with small‐molecule tyrosine kinase inhibitors (TKIs) [12, 13]. The quinazoline‐based reversible first‐generation (e.g., gefitinib, erlotinib) and irreversible second‐generation (e.g., afatinib) TKIs are ATP‐competitive inhibitors and exhibit mutant‐selective activity by exploiting the serendipitously lower ATP affinity of these mutants compared with the WT kinase domain [14]. However, patients eventually acquire drug resistance through the acquisition of a second “gatekeeper” T790M mutation in HRI of the kinase domain [15, 16], which renders these drugs ineffective by enhancing the kinase domain affinity for ATP substrate [17]. Overcoming T790M resistance eventually accumulated in the development of the third generation of EGFR TKIs (e.g., osimertinib and lazertinib) based on the anilinopyrimidine scaffold that binds selectively to the T790M‐containing kinase and is equipped with a Michael acceptor functional group poised to form an irreversible covalent bond with residue C797 [18, 19, 20, 21]. Clinical assessments have shown that both osimertinib and lazertinib are highly effective first‐line agents, exhibiting superior efficacy and progression‐free survival in treatment naïve settings [22, 23]. Despite the success of these irreversible agents, eventual disease progression occurs with a significant number of patients acquiring another tertiary C797S mutation, rendering these compounds unable to form their potency‐enabling covalent bonds [24, 25, 26]. Additionally, allosteric inhibitors that bind to the inactive conformation have been explored in this context, and several molecules show encouraging preclinical behavior [27, 28, 29]. Accordingly, overcoming T790M/C797S has emerged as the modern challenge in advancing treatments in EGFR NSCLC; ongoing drug discovery efforts are needed to advance more effective agents [30, 31, 32, 33, 34].

A recently emerged strategy to overcome EGFR T790M/C797S drug resistance, proposed by our lab and others, involves the discovery of AABIs that simultaneously bind to the ATP (orthosteric) and allosteric pockets [6, 35, 36, 37, 38, 39]. As such, two AABIs scaffolds have been previously developed and structurally characterized based on the allosteric site being a benzodiazepinone (benzo) [39] or phenyl‐methylisoindolinone (Figure 1D) [6]. Both of these compound series are derived from ATP‐site trisubstituted imidazole scaffolds that exhibit activity against C797S [40, 41]. While the benzo series exhibits significantly potent activity against LR/TM and LR/TM/CS (~50–60 pM), the isoindolinone series is broadly potent and selective against LR, TM, CS, and exon19del mutations and exhibits diminished activity against WT EGFR. Structural studies indicate that the binding of these AABIs responsible for their selective inhibitory profiles stems from their binding to the αC‐helix‐out channel of the allosteric pocket (orange in Figure 1C,D). This site reliably anchors six‐membered benzene‐like rings as seen in Type I½, III, and AABIs; however, limited exploration of diverse groups binding to this site has been explored with respect to their structure and activity. These indications have been made through the synthesis and testing of groups of the αC‐helix‐out channel binders stemming from a phenyl at HR‐II, where the moieties that are in the center of the ATP and allosteric sites are likely important for the potency and selectivity of AABIs. To aid in the discovery of mutant EGFR TKIs, we sought to further refine the understanding of groups that bind to the back pocket of the allosteric pocket in the inactive conformation of the EGFR kinase domain (Figure 1E). We focus our attention on the biochemical profiles of a variety of molecules against LR mutant EGFR kinase enzymes, as well as LR/TM, to assess the impact of the gatekeeper M790. We discover that certain amide‐mediating aryl functional groups enable effective inhibition of these two mutations, with the most effective being a combination of hydroxyl and fluoro groups. Functional profiling shows that the most active compounds suppress EGFR activation in human cancer cell line models. An X‐ray cocrystal structure of a 2,6‐difluorophenyl analog showcases the binding mode of these derivatives, which are discussed in the context of related Type I½ inhibitors and proximal Type V AABIs. These findings highlight the various groups and points of connection that can be utilized in the design of novel TKIs and further characterize the functional groups that produce effective AABIs targeting EGFR.

## 2 Results and Discussion

### 2.1 Chemistry

**Scheme 1:**
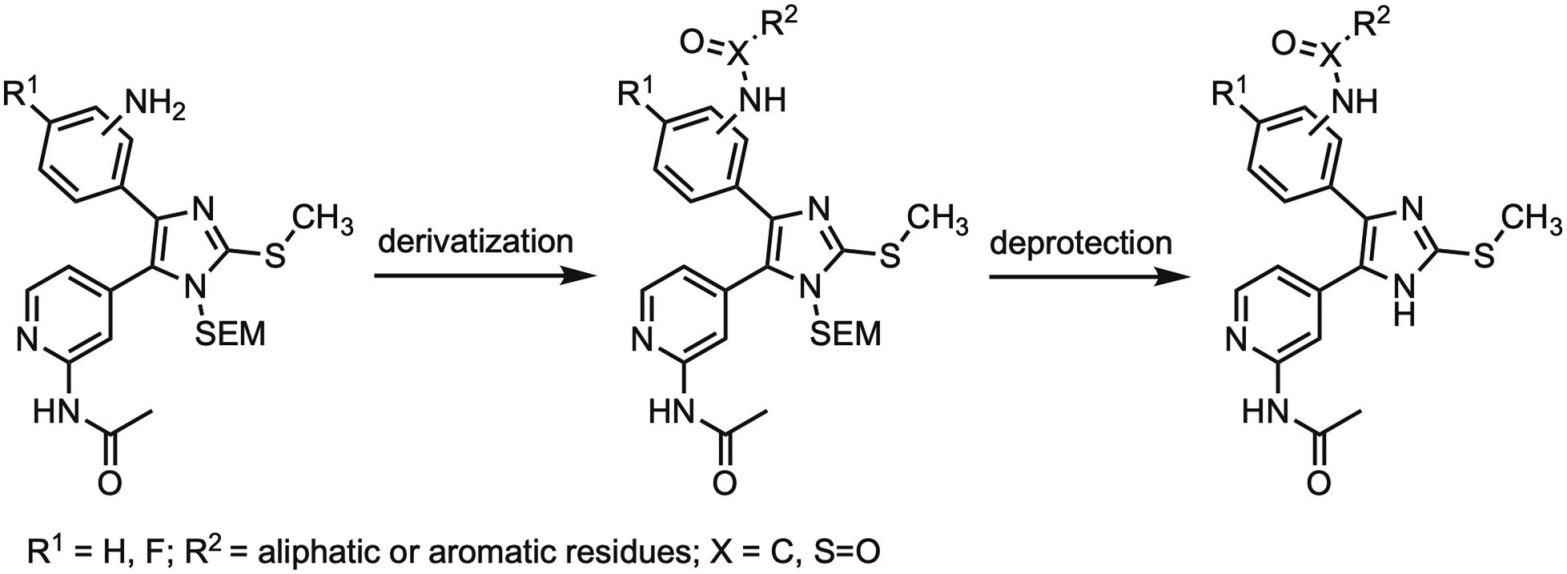
*Ortho*‐ and *meta*‐derivatization of a trisubstituted imidazole scaffold with varying functionalities to address the back pocket.

To evaluate the influences of functional groups within the back pocket and to deepen the understanding of EGFR Type I½ inhibitors, we synthesized a variety of compounds that are based on our previously established trisubstituted imidazole scaffold [40, 41, 42]. To enhance the accessibility of the back pocket for derivatization, we introduced an amino functionality at the linker region between ortho‐ and allosteric sites, since this modification allows for straightforward conversion into amides, sulfonamides, that is, functionalities which assist in the determination of the most optimal occupation of the adjacent pockets. Synthesis of imidazole scaffolds followed our previously described conditions with slight adaptations [38, 39, 40, 42] (Supporting Information S2: Schemes S1‐2), and the novel test compounds were finalized via acidic deprotection after derivatization (Scheme 1).

### 2.2 Biology

To begin, we generated SARs with a series of trisubstituted imidazole inhibitors prepared with various back‐pocket binding groups, on the basis of enzymatic inhibition for LR and LR/TM EGFR mutants assessed by a homogeneous time‐resolved fluorescence (HTRF) assay. Initially, the derivatization of *meta*‐substituted anilines was evaluated with a variety of aromatic groups (Table 1) that mimic earlier Type I½ and III inhibitors [5, 43, 44]. Beginning with compound **1**, which is unsubstituted and largely inactive, most derivatives featuring a back‐pocket motif demonstrated enhanced activity against the LR mutant enzyme. A series of benzoic acid‐derived compounds showed beneficial effects dependent on the fluorination pattern of the back pocket moiety (**2–7**), with a 2,6‐difluoro derivative (**6**) exhibiting the highest potency on both mutants. The introduction of a hydroxy group in 3‐position (**8–9**), analogous to allosteric inhibitor EAI045 [43], resulted in benefits to the potency of these inhibitors. Additionally, pyridinyl moieties within the back pocket showed inhibition for the 2‐ and 3‐substituted pyridinyl‐carboxylates (**10,11**); however, not as potent as inhibitors **6**, **8**, and **9**. The isonicotinic acid derivative (**12**) exhibited a significant drop in activity, highlighting a clear preference for the specific positioning of the nitrogen atom within the pyridine heterocycle. More unique bicyclic heterocycles were accommodated within the back pocket when oriented orthogonally to the amide as found in an *N*‐methylated indole derivative (**13**) and a 1‐napthoic acid derivative (**14**) that both showed modest potency against LR. The linear configuration of the 2‐napthoic acid derivative (**15**) resulted in full loss of activity. Additionally, 2‐ and 3‐substituted thiophenyls (**16,17**) showed IC_50_ values ~0.1 μM on LR enzyme when placed within the back pocket.

**Table 1:** Biochemical activities (IC_50_) against mutant EGFR of inhibitors with *meta*‐substituted amide‐linker derivatives.

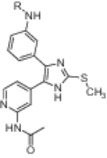			
Compound	R	EGFR IC_50_ [μM]^a^	
		LR	LR/TM
**1**	‐H	> 10	> 10^b^
**2**	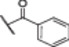	0.045 ± 0.004^c^	0.59 ± 0.03^c^
**3**	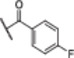	0.81 ± 0.1	> 10
**4**	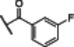	0.10 ± 0.01	0.73 ± 0.08
**5**	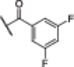	0.47 ± 0.08	> 10
**6**	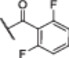	0.015 ± 0.003	0.10 ± 0.007
**7**	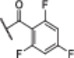	0.31 ± 0.04	3.9 ± 1
**8**	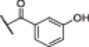	0.0052 ± 0.0003^c^	0.051 ± 0.004^c^
**9**	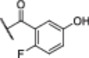	0.0055 ± 0.0003^d^	0.032 ± 0.003^d^
**10**	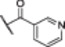	0.15 ± 0.01	1.83 ± 0.1
**11**	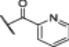	0.21 ± 0.01	2.1 ± 0.12
**12**	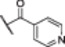	2.5 ± 0.3	> 10
**13**	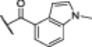	0.32 ± 0.03	1.4 ± 0.2
**14**	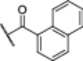	0.062 ± 0.005	0.22 ± 0.02
**15**	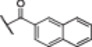	> 10	> 10
**16**	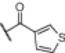	0.12 ± 0.007	2.4 ± 0.2
**17**	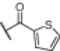	0.093 ± 0.02	1.5 ± 0.1

^a^ IC_50_ values were measured from nonlinear least squares fitting of HTRF activity data collected in triplicate. ATP concentration of 100 μM. Errors are reported as ± the standard error.

^b^ Data from Wittlinger and Ogboo et al. [39].

^c^ Data from Wittlinger et al. [6].

^d^ Data from Wittlinger and Heppner et al. [38].

We further assessed the enzymatic activity of sulfonamide derivatives that showed sub‐micromolar activity on LR for aliphatic (**18–20**) and micromolar activity for aromatic derivatives (**21**), the latter ~25 fold less active than its amide analog (**2**), wherefore this functionality appeared to be not suitable (Table 2). Additional evaluations of *ortho*‐substituted anilines also proved to be not potent on both enzymes, wherefore this linker was also excluded in further considerations (Supporting Information S2: Table S1). Implementation of an additional fluorine within the linker (**22–24**) resulted in comparable potencies on LR mutation (Table 3). However, this series suffered stronger from the gatekeeper mutation than their non‐fluorinated analogs (**6** ~ 0.10 μM compared with **23** ~ 1.20 μM). Overall, the SAR represented here from the *meta*‐aniline series showcases a range of diverse back‐pocket functional groups capable of inhibiting mutant LR EGFR. We found that derivatives with fluorine in 2,6‐positions and/or a hydroxy group in 3‐position of the back pocket phenyl yielded compounds with IC_50_ values ~5–15 nM on the LR mutant and 32–100 nM on the gatekeeper harboring LR/TM mutant.

**Table 2:** Biochemical activities against mutant EGFR of inhibitors with *meta*‐substituted sulfonamide‐linker derivatives.

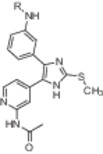			
Compound	R	EGFR IC_50_ [μM]^a^	
		LR	LR/TM
**18**	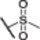	0.43 ± 0.06	7.7 ± 0.7
**19**	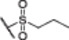	0.66 ± 0.03	1.8 ± 0.1
**20**	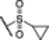	0.13 ± 0.01	5.1 ± 0.3
**21**	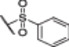	1.2 ± 0.08	1.5 ± 0.2

^a^ IC_50_ values were measured from nonlinear least squares fitting of HTRF activity data collected in triplicate. ATP concentration of 100 μM. Errors are reported as ± the standard error.

**Table 3:** Biochemical activities against mutant EGFR of inhibitors with a 4‐fluoro aniline linker.

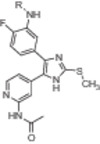			
Compound	R	EGFR IC_50_ [μM]^a^	
		LR	LR/TM
**22**	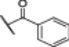	0.042 ± 0.004	1.2 ± 0.2
**23**	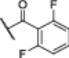	0.062 ± 0.008	1.2 ± 0.2
**24**	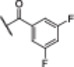	0.52 ± 0.07	> 10

^a^ IC_50_ values were measured from nonlinear least squares fitting of HTRF activity data collected in triplicate. ATP concentration of 100 μM. Errors are reported as ± the standard error.

**Figure 2:**
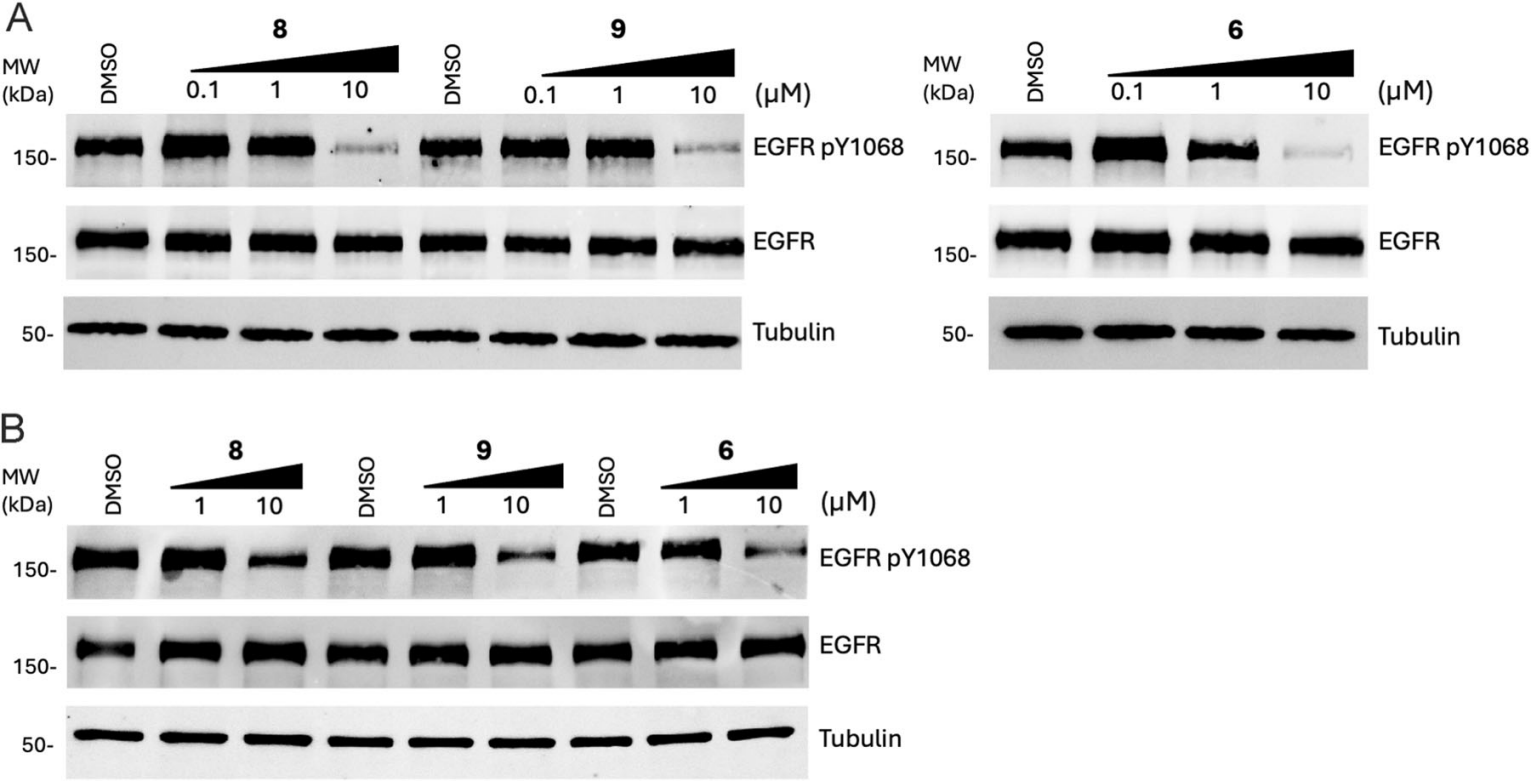
Immunoblotting of phosphorylation levels of EGFR in (A) H3255 (LR) and (B) H1975 (LR/TM) cells. All experiments performed after 6 h of treatments and Western blots are representative of three independent experiments.

We next sought to assess the ability of the most active molecules from our biochemical SAR to exhibit effects in cells. Specifically, we carried out experiments to determine the impact of these molecules on EGFR phosphorylation at Y1068, which is a hallmark of activated EGFR. Accordingly, the most effective compounds (**6**, **8**, and **9**) were dosed against human NSCLC cell line models H3255, H1975, and HCC827 as they contain the EGFR with activating mutations LR, LR/TM, and ex19del, respectively. Molecules were dosed against cell lines for 6 h and their activity was assessed by Western blot. Under these conditions, our compounds exhibited the ability to suppress pY1068 in H3255 at concentrations below 10 µM and to a lesser extent against H1975s, which is consistent with our biochemical activity measurements on EGFR LR enzyme and the expected impact of the TM gatekeeper mutation (Figure 2, Table 1). We also observed that these inhibitors are active against ex19del in HCC827 cells, although to a lesser extent compared with H3255 and H1975 cells (Supporting Information S2: Figure S2). While the impact on pY1068 EGFR is somewhat modest, these results are expected on the basis of potency values and cellular experiments in earlier papers [6, 38]. Overall, these results are encouraging, indicating that these molecules exhibit effects in mutant EGFR NSCLC cell line modes.

### 2.3 Structural Characterization With X‐Ray Crystallography

**Figure 3:**
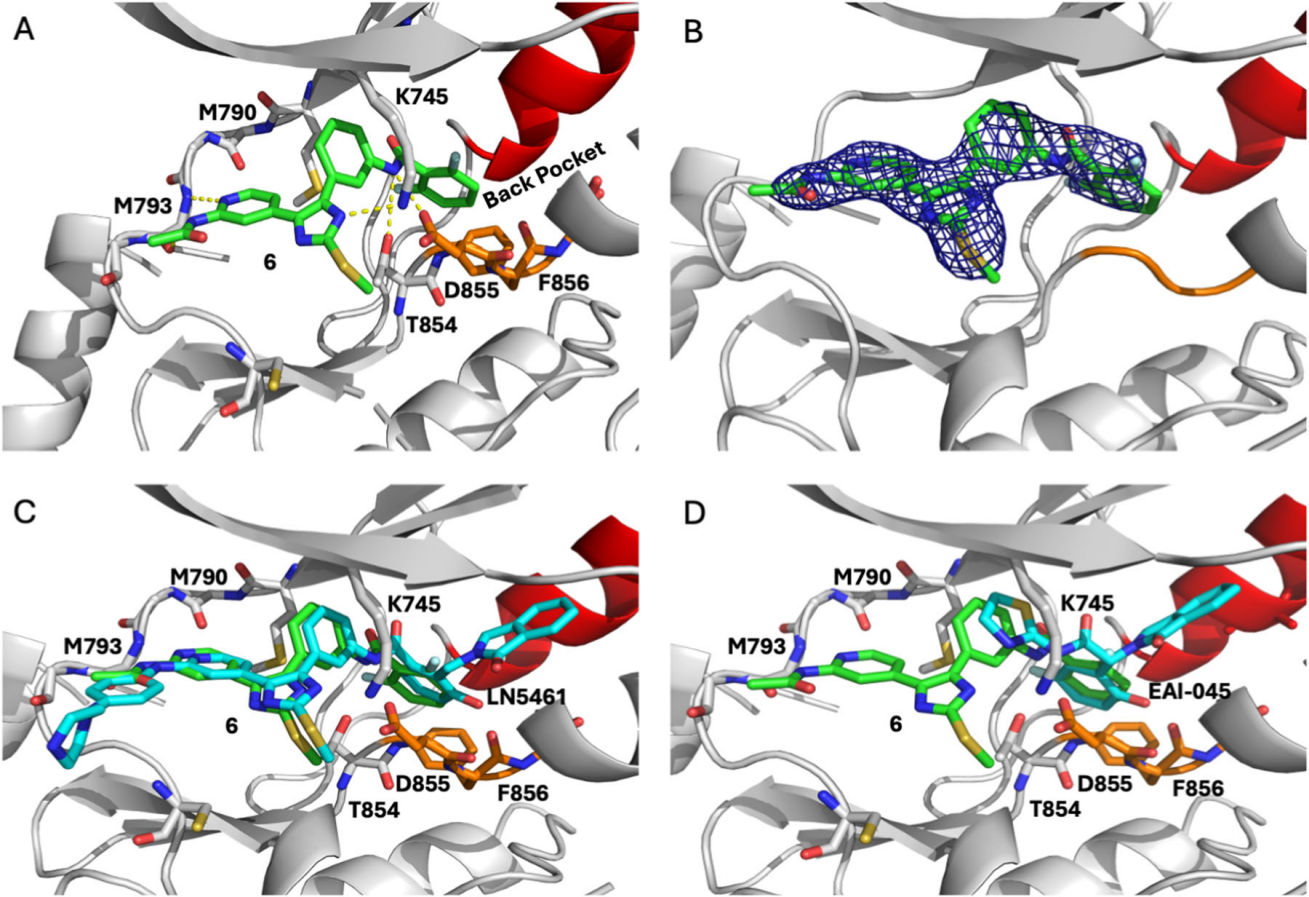
(A) Binding mode of **6** in complex with EGFR (TM/VR) with “back pocket” moiety 2,6‐difluoro‐labeled for clarity (PDB code 9N6G). (B) Corresponding m*F*_o_–D*F*_c_ simulated annealing omit map contoured to 3σ. Superposition of cocrystal structures of **6** (green) with (C) LN5461 (cyan, PDB code 6WA2), (D) EAI045 (cyan, PDB code 6P1L). In all structures, the P‐loop of the kinase domain is omitted for clarity.

To confirm the binding mode of these compounds, we succeeded in obtaining an X‐ray cocrystal structure of EGFR(T790M/V948R) crystals soaked with **6**. The inclusion of the V948R mutation is critical to reinforcing in the inactive αC‐helix “out” conformation with an accessible allosteric pocket [6]. We fitted **6** binding within a ~2.6 Å‐resolution X‐ray cocrystal structure, which showed expected anchoring of the trisubstituted imidazole at the ATP site and 2,6‐difluorophenyl group within the back pocket (Figure 3A, Supporting Information S2: Table S2). The aminopyridine interacts as previously observed with the backbone of M793 at the hinge region, and the imidazole moiety is engaged in an H‐bond with catalytic lysine (K745). The amide linker is anchored through H‐bonds to T854 and D855 of the DFG motif that positions the 2,6‐difluorophenyl within the back pocket. Accordingly, this group binds through a π‐stacking interaction with the side chain of F856 of the DFG motif. The binding mode of inhibitor **6** is practically analogous to that of proximal Type V inhibitor LN5461 (Figure 3C) and the allosteric inhibitor EAI045 (Figure 3D), indicating that the back pocket groups share interactions with these earlier examples of EGFR inhibitors.

## 3 Discussion

Structure‐guided design of bivalent compounds often relies on the search for functional groups that effectively link adjacent‐site binding groups [45]. One prominent example of successful drug discovery is the emergence of Type I½ inhibitors that anchor groups into the back pocket of the allosteric site reinforcing the inactive kinase domain conformation [5]. More recently, developments in EGFR TKIs at the canonical ATP site as well as the allosteric site have motivated the development of ATP‐allosteric bivalent inhibitors (AABIs) as a new class of compounds in the arsenal against this important target [6, 35, 36, 37, 38, 39]. In this study, we sought to better understand the structure and activity of the functional groups at the center of these AABIs. By variation within the back pocket of the αC‐helix out‐conformation, SARs confirm that *meta*‐aniline linkers are best suited for the preparation of inhibitors that extend into the back pocket, and six‐membered aryl functional groups with hydroxyl and fluoro substituents within the back pocket exhibit the highest potency. These observations are consistent with our previously reported compounds [6, 38] as well as other examples [35, 36, 37] and Type I½ TKIs [46, 47, 48].

The scope of molecular groups surveyed in this study indicates a relatively tight SAR of the back pocket in the context of trisubstituted imidazole EGFR TKIs. We observe that the most active analogs are *meta*‐substituted anilines with *meta*‐substituted sulfonamides and *ortho*‐substituted anilines being generally less effective. The minimal efficacy of *ortho*‐substituted anilines likely stems from steric incompatibility with the kinase domain. Similarly, more electropositive *meta‐*sulfonamides exhibit lower activities, which is consistent with weaker binding due to the inability to accommodate a larger polar linker compared with more potent amides (e.g., **8**,**9**). Additionally, we evaluated an array of heterocyclic ring architectures, including six‐membered phenyl, five‐membered cyclopentyl, fused bicyclic, and aliphatic derivatives within the back pocket. We see that hydrophobic phenyl rings exhibit the highest potency where activity can be modulated upon incorporation of fluorine and hydroxyl groups in potency ranging from ~15–800 nM for LR and 100–10^4 ^nM for the LR/TM mutants. Notably, the 1‐napthoic acid derivative (**14**) and thiophenyl carboxylates (**16**,**17**) also showed promising activity against the LR enzyme. Finally, pyridinyl moieties were active as well but varied in efficacy due to the positioning of the nitrogen atom and were highly sensitive to the TM gatekeeper mutation. These Type I½ inhibitors were seen to suffer more from the TM mutation as compared with ATP‐allosteric bivalent inhibitors (AABIs) (Supporting Information S2: Table S3). Overall, these results demonstrate that the back pocket region between the ATP and allosteric sites of EGFR is ideally targeted by substituted phenyl rings bearing fluorine or hydroxyl groups, in the context of trisubstituted imidazole TKIs. While several alternative moieties also exhibit activity—albeit to a lesser extent within our TKIs—our SAR highlights multiple linker options that may be valuable for other kinase drug discovery campaigns targeting alternative structures within the HR‐I and allosteric site back pockets.

**Figure 4:**
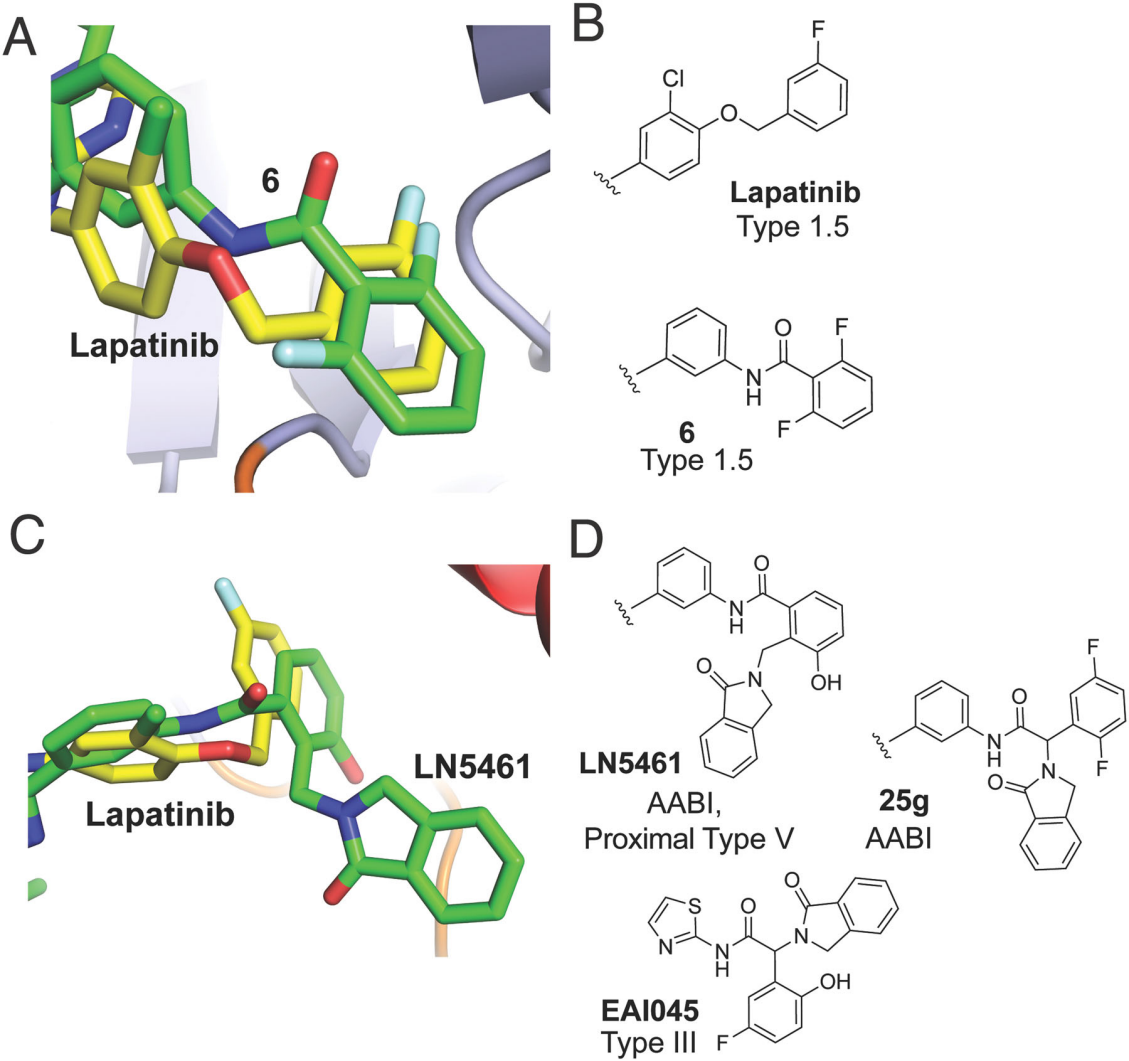
Comparison of “points of connection” to the back pocket in EGFR Type I½, III, and AABI scaffolds. (A) Overlay of X‐ray cocrystal structure binding modes of lapatinib (yellow, PDB ID 1XKK) and **6** (green, PDB ID 9N6G) and (B) chemical structures of the back pocket binding groups. (C) Overlay of lapatinib (PDB ID 1XKK) and LN5461 (PDB ID 8TO3), and (D) chemical structures of relevant AABIs as well as allosteric EAI045.

An important structural and design perspective in the context of EGFR kinase inhibitors is to consider these findings in the context of the FDA‐approved Type I½ TKIs lapatinib and the irreversible analog neratinib [48]. Both of these compounds consist of anilinoquinazoline ATP‐site scaffolds, which are closely related to first‐ and second‐generation EGFR inhibitors; however, they extend methoxy‐linked fluorophenyl or pyridinyl groups into the back pocket. While these back‐pocket groups comprise six‐membered rings, as is also the case for our most active molecules (**6**,**8**,**9**), they are unique structurally by the groups that link the HR‐I to the back pocket. Specifically, the back pocket binding group is attached to C‐4 of the linking ring in lapatinib, while for **6**, the point of connection is at C‐3 (Figure 4A,B). The phenyl ring is practically identical in position between lapatinib and **6;** however, the chlorination of lapatinib appears to shift the HR‐I moiety in the back pocket (Figure 1A), which perhaps necessitates this alternative point of connection to the back pocket phenyl between these two scaffolds (Figure 1B). Additionally, mutant‐selective AABIs deviate in the positioning of the back pocket when compared with lapatinib and may reflect a change in binding that is due to the additional isoindolinone moiety in the αC‐helix‐out channel (Figure 4C). While not structurally characterized, other AABI molecules are known and consist of tribladed allosteric groups coupled to quinazoline ATP‐site binding groups (e.g., 25 g in Figure 4D) [37]. The molecules surveyed in this study are largely differentiated from established molecules in the literature in their “point of connection” from HR‐I to the back pocket, which we have shown to be critical for optimizing bivalent scaffolds [39]. Additionally, it is also crucial to compare our inhibitors with Type III inhibitors such as EAI045, which are highly mutant‐selective and bind within an allosteric pocket created by the outward displacement of the αC‐helix [27, 43]. The allosteric‐only EAI045 and structurally related inhibitors have a connection at the back pocket that is more similar to lapatinib and triblade AABIs (Figures 3D and 4D) [37, 43, 46]. These observations additionally indicate that the point of connection at the allosteric site represents two distinct functional attributes of EGFR TKIs, further showcasing that optimization at these positions may be critical for arriving at an ideally active agent.

## 4 Conclusion

In summary, various back‐pocket binding groups were synthesized and tested to generate SAR for trisubstituted imidazole Type I½ EGFR TKIs. These results indicate that a variety of groups can be used to link the ATP and allosteric pockets, with the most effective being *meta*‐substituted aniline linkers. Additionally, potent molecules exhibit modest cellular activity in NSCLC cancer cell line models and exhibit binding to the inactive kinase domain as observed via X‐ray crystal structure. The back pocket appears to best accommodate substituted six‐member aryl rings through a variety of electronic intermolecular interactions, which can be further attenuated through hydrophobic and hydrogen bond acceptors of the 2‐fluoro and 5‐hydroxy groups of the benzamide (e.g., **9** and EAI045). The linker architecture in these trisubstituted imidazoles is distinct from quinazoline TKIs, indicating the importance of different points of connection with distinct ATP‐site groups. These results showcase an alternative point of connection from HR‐I to the allosteric site back pocket, which is under‐explored in Type I½ inhibitors. This study justifies further considerations of kinase inhibitors through the survey of structurally diverse moieties that target the inactive conformation.

## 5 Experimental

### 5.1 Chemistry

#### 5.1.1 General

All chemicals, reagents, and dry solvents were purchased from commercial sources and were used without further purification or drying procedures unless otherwise noted. Thin‐layer chromatography (TLC) was performed on fluorescent silica gel 60 F254 aluminum plates from Merck (Darmstadt, Germany) or Alugram Sil G/UV254 plates from Macherey‐Nagel (Düren, Germany) and visualized via UV illumination at 254 and 366 nm. High‐performance liquid chromatography (HPLC) was performed on an Agilent 1100 Series LC or Hewlett‐Packard HP 1090 Series II with a Phenomenex Luna C8 column (150 × 4.6 mm, 5 μm), and detection was performed using a UV diode array detector (DAD) at 254 and 230 nm wavelengths. Elution was carried out with the following gradient: 0.01 M KH_2_PO_4_, pH 2.30 (solvent A), and MeOH (solvent B), 40% B to 85% B in 8 min, 85% B for 5 min, 85% to 40% B in 1 min, 40% B for 2 min, stop time of 16 min, 5 μL injection volume, flow rate of 1.5 mL/min, and 25°C oven temperature. Column chromatography was performed on an Interchim (Montlucon, France) PuriFlash XS 420 automated flash chromatography system. Davisil LC60A 20–45 μm silica from Grace Davison (Columbia, USA) was used as the stationary phase and Geduran Si60 63–200 μm silica from Merck (Darmstadt, Germany) for the precolumn. The applied mobile phases for purified compounds are given in the experimental procedures. Nuclear magnetic resonance (NMR) spectra were obtained with Avance 200, 400, and 600 MHz or Ascend 400 MHz instruments from Bruker (Billerica, MA, USA). Residual solvent peaks were used to calibrate the chemical shifts. Chemical shifts (δ) are reported in parts per million based on TMS (*δ* = 0). Clearly assignable multiplicities are abbreviated as follows: s (singlet), d (doublet), t (triplet), q (quartet), dd (double double), br (broad signal), and m (multiplet, denotes complex multiplicity or applied at low‐resolution NMR experiments). Depending on their substitution patterns, imidazoles can display a mixture of tautomers in their NMR spectra. In such cases, clearly assignable signals were combined in 1H spectra. 13 C spectra were omitted when signals were not assignable and did not assist in the identification of compounds. Multiplicity of C‐F coupling in 13 C spectra was reported when signals were clearly assignable. Mass spectra were obtained with TLC‐MS (Expression CMS instrument from Advion [Ithaca, NY, USA] coupled to Advion Plate Express TLC plate reader from Advion [Ithaca, NY, USA]) or with ASAP‐MS (Expression CMS instrument from Advion [Ithaca, NY, USA] coupled to ASAP [Atmospheric Solids Analysis Probe] from Advion [Ithaca, NY, USA]). Samples were ionized via electrospray ionization (ESI) or atmospheric pressure chemical ionization (APCI). ESI (+) and (−) modes: 250°C capillary temperature, 180 V capillary voltage, 250°C gas temperature, and 3500 V or 2500 V ESI voltage, respectively. APCI (+) and (‐) modes: 190°C capillary temperature, 3.4 V capillary voltage, 281°C gas temperature, and 0.32 µA APCI current, respectively. ESI‐HRMS spectra were obtained from the MASS Spectrometry Department (ESI‐HRMS), Institute of Organic Chemistry, Eberhard‐Karls‐Universität (Tübingen, Germany). Preparation of compounds described here can also be found in US20230406838A1.

The InChI codes of the investigated compounds, together with some biological activity data, are provided as Supporting Information.

#### 5.1.2 General Procedure A

A corresponding amine, a corresponding carboxylic acid, and an appropriate coupling reagent (1.2–2.2 eq of PyBOP, TBTU, EDC‐HCl, or HATU) were dissolved in dry DMF (0.05 M), and a base (1.5–3.0 eq of TEA or DIPEA) was added to the solution. The reaction mixture was stirred overnight at ambient temperature. After the addition of brine, the aqueous phase was extracted several times with DCM, the combined organic layers were dried over Na_2_SO_4_, filtered, and volatiles were removed in vacuo. The crude product was purified via flash chromatography.

#### 5.1.3 General Procedures B

B1: The starting material was dissolved in a mixture of 20%–33% v/v TFA in DCM (0.05 M). The mixture was stirred at ambient temperature overnight. After complete consumption of the starting material, volatiles were removed by rotary evaporation, and the oily residue was diluted with DCM. A saturated NaHCO_3_ solution was added, and the aqueous layer was extracted several times with DCM or EtOAc. The combined organic layers were dried over Na_2_SO_4_, filtered, and the solvents were removed in vacuo. The crude product was purified via flash chromatography.

B2: The starting material was dissolved in 2.5 M HCl in Ethanol (0.05 M) or 4.0 N HCl in 1,4‐dioxane (0.05 M) and stirred at ambient temperature until complete consumption of the starting material (24–72 h). The mixture was concentrated in vacuo, and the oily residue was suspended in saturated aqueous NaHCO_3_ solution. The product was extracted several times with EtOAc or DCM. The combined organic layers were dried over Na_2_SO_4_, filtered, and the solvents were removed by rotary evaporation. The crude product was purified via flash chromatography.

#### 5.1.4 Synthesis and Characterization

*N*‐(4‐(4‐(3‐Aminophenyl)−2‐(methylthio)−1‐((2‐(trimethylsilyl)ethoxy)methyl)−1*H*‐imidazol‐5‐yl)pyridin‐2‐yl)acetamide (**S1**): Prepared as previously described [38].

2‐(4‐Fluoro‐3‐nitrophenyl)−4,4,5,5‐tetramethyl‐1,3,2‐dioxaborolane (**S2**): The title compound was synthesized from 1000 mg (4.54 mmol) 4‐bromo‐1‐fluoro‐2‐nitrobenzene, 1212 mg (4.77 mmol, 1.05 eq.) bis(pinacolato)diboron, and 1338 mg (13.63 mmol, 3.0 eq.) KOAc according to standard Miyaura borylation protocol under argon atmosphere with Pd(dppf)Cl_2_ (5 mol%). The product was precipitated in *n‐*hexane and filtered to obtain a yellow solid in quant. yield (1240 mg, 4.54 mmol). ^1^H NMR (200 MHz, CDCl_3_) δ 8.56–8.42 (m, 1H), 8.13–8.00 (m, 1H), 7.37–7.23 (m, 1H), 1.38 (s, 12H). ^13^C NMR (50 MHz, CDCl_3_) *δ* 160.1, 154.8, 142.0, 141.8, 132.6, 132.5, 118.1, 117.7, 84.9, 24.9.

4‐(4‐Fluoro‐3‐nitrophenyl)−2‐(methylthio)−1‐((2‐(trimethylsilyl)ethoxy)methyl)−1*H*‐imidazole (**S3**): The title compound was synthesized from 950 mg (2.94 mmol) 4‐bromo‐2‐(methylthio)−1‐((2‐(trimethylsilyl)ethoxy)methyl)−1*H*‐imidazole, 1020 mg (3.82 mmol, 1.3 eq.) S2, and 1871 mg (1.5 M aq., 8.81 mmol, 3.0 eq.) K_3_PO_4_ according to standard Suzuki cross coupling protocol under argon atmosphere with P(*t*‐Bu)_3_ Pd G3 (5 mol%). The crude product was purified via flash chromatography (SiO_2_, *n*‐hexane/EtOAc 7:3) to give 940 mg (2.45 mmol) of the pure product as a pale‐yellow solid in 83% yield. ^1^H NMR (200 MHz, CDCl_3_) *δ* 8.40 (dd, *J* = 7.1, 2.1 Hz, 1H), 8.03 (ddd, *J* = 8.6, 4.1, 2.2 Hz, 1H), 7.38 (s, 1H), 7.25 (t, *J* = 9.6 Hz, 1H), 5.28 (s, 2H), 3.60–3.50 (m, 2H), 2.68 (s, 3H), 0.98–0.87 (m, 2H), −0.02 (s, 9H). ^13^C NMR (50 MHz, CDCl_3_) *δ* 154.4 (d, *J* = 264.3 Hz), 145.3, 139.2, 131.6 (d, *J* = 8.3 Hz), 131.2, 131.1, 122.0 (d, *J* = 2.8 Hz), 118.6 (d, *J* = 21.2 Hz), 117.5, 75.4, 66.8, 17.9, 16.5, −1.3. TLC‐MS (ESI): calcd. *m/z* 383.11 for C_16_H_22_FN_3_O_3_SSi, found 383.9 [M + H]^+^.

5‐Bromo‐4‐(4‐fluoro‐3‐nitrophenyl)−2‐(methylthio)−1‐((2‐(trimethylsilyl)ethoxy)methyl)−1*H*‐imidazole (**S4**): To begin, 900 mg (2.34 mmol) S3 was dissolved in ACN and cooled down to −30°C under an inert atmosphere. A total of 418 mg (2.34 mmol, 1.0 eq.) NBS dissolved in ACN was added dropwise to the stirring solution. After complete conversion, aqueous, saturated sodium sulfite solution was added, and the aqueous layer was extracted several times with EtOAc. The combined organic layers were dried over Na_2_SO_4_, filtered, and evaporated to dryness. The crude product was purified via flash chromatography (SiO_2_, *n*‐hexane/EtOAc 7:3) to obtain 950 mg (2.05 mmol) of the pure product as a pale‐yellow solid in 87% yield. ^1^H NMR (200 MHz, CDCl_3_) δ 8.72 (dd, *J* = 7.2, 2.3 Hz, 1H), 8.29 (ddd, *J* = 8.8, 4.3, 2.3 Hz, 1H), 7.37–7.26 (m, 1H), 5.36 (s, 2H), 3.68–3.58 (m, 2H), 2.69 (s, 3H), 1.01–0.91 (m, 2H), 0.00 (s, 9H). ^13^C NMR (50 MHz, CDCl_3_) δ 154.6 (d, *J* = 265.2 Hz), 146.4, 136.2, 133.3 (d, *J* = 8.4 Hz), 130.3, 130.2, 123.9 (d, *J* = 2.9 Hz), 118.4 (d, *J* = 21.1 Hz), 101.3, 74.1, 67.1, 18.0, 16.0, −1.3.

*N*‐(4‐(4‐(4‐Fluoro‐3‐nitrophenyl)−2‐(methylthio)−1‐((2‐(trimethylsilyl)ethoxy)methyl)−1*H*‐imidazol‐5‐yl)pyridin‐2‐yl)acetamide (**S5**): The title compound was synthesized from 920 mg (1.99 mmol) **S4**, 782 mg (2.99 mmol, 1.5 eq.) *N*‐(4‐(4,4,5,5‐tetramethyl‐1,3,2‐dioxaborolan‐2‐yl)pyridin‐2‐yl)acetamide, and 1267 mg (1.5 M aq., 5.97 mmol, 3.0 eq.) K_3_PO_4_ according to standard Suzuki cross coupling protocol under argon atmosphere with P(*t*‐Bu)_3_ Pd G3 (5 mol%). The crude product was purified via flash chromatography (SiO_2_, *n*‐hexane/EtOAc 2:8) to give 770 mg (1.49 mmol) of the pure product as a yellow solid in 75% yield. ^1^H NMR (200 MHz, CDCl_3_) *δ* 8.73 (s, 1H), 8.35–8.28 (m, 2H), 8.22 (dd, *J* = 7.2, 2.2 Hz, 1H), 7.70 (ddd, *J* = 8.6, 4.2, 2.3 Hz, 1H), 7.18–7.05 (m, 2H), 5.18 (s, 2H), 3.59–3.48 (m, 2H), 2.74 (s, 3H), 2.20 (s, 3H), 0.97–0.87 (m, 2H), −0.03 (s, 9H). ^13^C NMR (50 MHz, CDCl_3_) δ 168.9, 154.5 (d, *J* = 265.2 Hz), 152.4, 148.4, 147.1, 140.3, 137.4 (d, *J* = 7.7 Hz), 137.1, 133.9 (d, *J* = 8.2 Hz), 131.1 (d, *J* = 4.1 Hz), 128.7, 124.6 (d, *J* = 2.8 Hz), 121.0, 118.6, 118.4 (d, *J* = 21.0 Hz), 73.2, 66.8, 24.8, 17.9, 16.2, −1.3. TLC‐MS (ESI): calcd. *m/z* 517.16 for C_23_H_28_FN_5_O_4_SSi, found 540.0 [M+Na]^+^.

*N*‐(4‐(4‐(3‐Amino‐4‐fluorophenyl)−2‐(methylthio)−1‐((2‐(trimethylsilyl)ethoxy)methyl)−1*H*‐imidazol‐5‐yl)pyridin‐2‐yl)acetamide (**S6**): The title compound was synthesized from 570 mg (1.10 mmol) **S5** and 719 mg (11.0 mmol, 10 eq.) zinc dust and 589 mg (11.0 mmol, 10 eq.) NH_4_Cl were added portion‐wise over 1 h. After complete conversion, the mixture was diluted with MeOH and filtered over celite. Solvents were evaporated, and the residue was suspended in EtOAc and again filtered over celite. The crude product was obtained in quantitative yield (600 mg, 1.10 mmol) as a yellow solid and used directly in the next step without further purification. ^1^H NMR (200 MHz, CDCl_3_) *δ* 9.48 (s, 1H), 8.28 (d, *J* = 5.5 Hz, 1H), 8.19 (s, 1H), 7.15–7.03 (m, 1H), 6.99–6.88 (m, 1H), 6.86–6.74 (m, 1H), 6.72–6.59 (m, 1H), 5.21 (s, 2H), 3.81–3.37 (m, 4H), 2.70 (s, 3H), 2.23–2.14 (m, 3H), 1.01–0.89 (m, 2H), −0.02 (s, 9H). TLC‐MS (ESI): calcd. *m/z* 487.19 for C_23_H_30_FN_5_O_2_SSi, found 510.0 [M+Na]^+^.

2‐Bromo‐1‐(2‐nitrophenyl)ethan‐1‐one (**S7**): To begin, 10.1 g (61.2 mmol) 2‐nitroacetophenone was dissolved in 100 ml CHCl_3_ (~0.6 M). 2 drops of conc. bromine were added to the solution, and the mixture was stirred until decolorization at ambient temperature. A volume of 3.29 mL bromine (64.2 mmol, 1.05 eq) dissolved in 100 mL CHCl_3_ was added dropwise at ambient temperature under vigorous stirring. After complete addition, the mixture was stirred for 30 min at room temperature, whereupon an aqueous saturated sodium sulfite solution was added, and the mixture was extracted three times with DCM. The combined organic layers were dried over Na_2_SO_4_, filtered, and the solvents were removed in vacuo. The crude product was isolated as yellow oil in quant. yield (15.4 g) and was used in the next step without further purification. ^1^H NMR (200 MHz, CDCl_3_) *δ* 8.28–8.16 (m, *J* = 8.9 Hz, 1H), 7.83–7.73 (m, 1H), 7.71–7.61 (m, 1H), 7.48 (dd, *J* = 7.3, 1.1 Hz, 1H), 4.28 (s, 2H). ^13^C NMR (50 MHz, CDCl_3_) δ 194.4, 135.1, 134.9, 131.9, 131.4, 129.2, 124.6, 33.9.

2‐Amino‐1‐(2‐nitrophenyl)ethan‐1‐one hydrochloride (**S8**): To begin, 15.0 g (61.5 mmol) **S7** was dissolved in 130 mL CHCl_3_ (~0.3 M). 8.62 g Hexamethylenetetramine (61.5 mmol, 1.0 eq.) was added portion‐wise, and the suspension was stirred overnight at 70°C. After cooling to ambient temperature, solids were collected by filtration and washed with CHCl_3_. The dried solid was resuspended in 100 mL EtOH and treated with 50 mL of concentrated HCl solution. The suspension was stirred overnight at ambient temperature. The mixture was cooled to 0°C, filtered, and the solids were washed with EtOH. The crude product was obtained with 70% yield (9.4 g, 43.4 mmol) as a white solid and used in the next step without further purification. ^1^H NMR (200 MHz, MeOD) δ 8.28–8.18 (m, 1H), 7.97–7.75 (m, 3H), 4.48 (s, 2H). ^13^C NMR (50 MHz, MeOH) δ 196.0, 147.5, 135.8, 134.3, 133.6, 129.5, 125.8.

4‐(2‐Nitrophenyl)−1,3‐dihydro‐2*H*‐imidazole‐2‐thione (**S9**): To begin, 9.4 g (43.4 mmol) **S8** was suspended in 150 mL glacial acetic acid. 4.7 g (47.7 mmol, 1.1 eq.) KSCN was added in one portion under vigorous stirring, and the mixture was refluxed for 1 h. After complete reaction, the mixture was cooled to 0°C, and solids were collected by filtration. Demineralized water was added to the filtrate, and the aqueous layer was extracted several times with DCM. Solids from filtration and organic layers of the extraction were combined, and solvents were removed in vacuo. The crude product was obtained in 29% yield (2.8 g, 12.65 mmol) and used without further purification. ^1^H NMR (200 MHz, DMSO) *δ* 12.47 (s, 1H), 12.29 (s, 1H), 8.12–7.98 (m, *J* = 7.7 Hz, 1H), 7.82–7.71 (m, 1H), 7.67–7.56 (m, *J* = 6.7, 3.5 Hz, 2H), 7.07 (s, 1H). ^13^C NMR (50 MHz, DMSO) *δ* 161.9, 147.1, 133.5, 131.7, 129.7, 124.8, 124.7, 122.6, 114.6.

2‐(Methylthio)−4‐(2‐nitrophenyl)−1*H*‐imidazole (**S10**): To begin, 2.8 g (12.65 mmol) **S9** and 2.1 g (15.19 mmol, 1.2 eq.) K_2_CO_3_ were suspended in 75 mL MeOH, and the mixture was vigorously stirred for 30 min at ambient temperature. A volume of 827 µL of methyl iodide (13.29 mmol, 1.05 eq.) was added dropwise, and the mixture was stirred overnight at ambient temperature. After complete consumption of the starting material, water was added, and the aqueous layer was extracted several times with EtOAc. The combined organic layers were dried over Na_2_SO_4_, filtered, and the solvents were removed in vacuo. The crude product was purified via flash chromatography (SiO_2_, DCM → DCM/MeOH 95:5; SiO_2_, *n*‐hexane to *n*‐hexane/EtOAc 1:1) to obtain 1.7 g (7.34 mmol, 58%) of the pure product as a white solid. ^1^H NMR (200 MHz, DMSO) *δ* 12.45 (s, 1H), 7.80–7.50 (m, 4H), 7.45–7.32 (m, 1H), 2.47 (s, 3H). ^13^C NMR (50 MHz, DMSO) δ 148.0, 142.0, 136.1, 131.5, 129.0, 127.7, 126.8, 123.3, 116.9, 15.2. TLC‐MS (ESI): calcd. *m/z* 235.04 for C_10_H_9_N_3_O_2_S, found 235.7 [M + H]^+^.

2‐(Methylthio)−4‐(2‐nitrophenyl)−1‐((2‐(trimethylsilyl)ethoxy)methyl)−1*H*‐imidazole (**S11**): To begin, 1.7 g (7.22 mmol) **S10** was dissolved in 60 mL THF (0.125 M) and cooled to −15°C under an argon atmosphere. 346 mg (8.67 mmol, 1.2 eq., 60% disp. in mineral oil) sodium hydride was added portion‐wise under vigorous stirring, and the mixture was stirred for 10 min at −10°C. 1.34 mL (7.58 mmol, 1.05 eq.) SEM‐Cl dissolved in 30 mL THF (0.25 M) was added dropwise, and the mixture was stirred for 2 h while slowly warming up to ambient temperature. A saturated aqueous NH_4_Cl solution was added, and the aqueous layer was extracted several times with EtOAc. Combined organic layers were dried over Na_2_SO_4_, filtered, and evaporated. The residue was dissolved in 12 mL of ACN and cat. amounts of SEM‐Cl (65 µL, 0.05 eq.) were added under an argon atmosphere. The flask was sealed and stirred at 80°C for 1 h. The crude product was purified via flash chromatography (SiO2, *n*‐hexane to *n*‐hexane/EtOAc 7:3) to give 2 g of the pure product as a colorless oil in 76% (5.52 mmol) yield. ^1^H NMR (200 MHz, CDCl_3_) *δ* 7.95–7.86 (m, 1H), 7.67–7.50 (m, 2H), 7.42–7.28 (m, 2H), 5.26 (s, 2H), 3.55 (t, 2H), 2.65 (s, 3H), 0.93 (t, 2H), − 0.00 (s, 9H). ^13^C NMR (50 MHz, CDCl_3_) *δ* 148.6, 144.8, 136.5, 131.8, 130.2, 127.7, 127.3, 123.6, 119.7, 75.3, 66.7, 17.9, 16.4, −1.3. TLC‐MS (ESI): calcd. *m/z* 365.12 for C_16_H_23_N_3_O_3_SSi, found 387.9 [M+Na]^+^.

5‐Bromo‐2‐(methylthio)−4‐(2‐nitrophenyl)−1‐((2‐(trimethylsilyl)ethoxy)methyl)−1*H*‐imidazole (**S12**): To begin, 2 g (5.51 mmol) **S11** was dissolved in ACN and cooled down to −30°C under an inert atmosphere. 1030 mg (5.79 mmol, 1.05 eq.) NBS dissolved in ACN was added dropwise to the stirring solution. After complete conversion, an aqueous, saturated sodium sulfite solution was added, and the aqueous layer was extracted several times with EtOAc. The combined organic layers were dried over Na_2_SO_4_, filtered, and evaporated to dryness. The crude product was purified via flash chromatography (SiO2, *n*‐hexane to *n‐*hexane/EtOAc 7:3) to give 2.2 g of the pure product as a pale‐yellow oil in 89% (5.51 mmol) yield. ^1^H NMR (200 MHz, CDCl_3_) *δ* 7.87 (dd, *J* = 8.0, 0.9 Hz, 1H), 7.74–7.55 (m, 2H), 7.53–7.42 (m, 1H), 5.35 (s, 2H), 3.62 (t, 2H), 2.62 (s, 3H), 0.95 (t, 2H), 0.01 (s, 9H). ^13^C NMR (50 MHz, CDCl_3_) *δ* 149.3, 146.2, 136.3, 132.3, 131.8, 128.9, 127.4, 124.6, 102.4, 74.2, 67.0, 18.0, 16.1, −1.3. TLC‐MS (ESI): calcd. *m/z* 443.03, 445.03 for C_16_H_22_BrN_3_O_3_SSi, found 466.0, 468.0 [M+Na]^+^.

*N*‐(4‐(2‐(Methylthio)−4‐(2‐nitrophenyl)−1‐((2‐(trimethylsilyl)ethoxy)methyl)−1*H*‐imidazol‐5‐yl)pyridin‐2‐yl)acetamide (**S13**): The title compound was synthesized from 500 mg (1.12 mmol) **S12**, 442 mg (1.69 mmol, 1.5 eq.) *N*‐(4(4,4,5,5‐tetramethyl‐1,3,2‐dioxaborolan‐2‐yl)pyridin‐2‐yl) acetamide, an aqueous solution of K_3_PO_4_ (1.5 M, 3.38 mmol, 3 eq.), and 32 mg (0.06 mmol, 5 mol%.) of P(*t*‐Bu)_3_ Pd G3 according to standard Suzuki cross coupling protocol under argon atmosphere. The crude product was purified via flash chromatography (SiO_2_, *n*‐hexane→*n*‐hexane/EtOAc 3:7) to give 370 mg of the pure product as a pale‐yellow solid in 65% (0.74 mmol) yield. ^1^H NMR (200 MHz, DMSO) *δ* 10.60 (s, 1H), 8.30 (d, *J* = 5.2 Hz, 1H), 8.05 (s, 1H), 7.93–7.82 (m, 1H), 7.62–7.46 (m, 2H), 7.32–7.20 (m, 1H), 7.04–6.94 (m, 1H), 5.24 (s, 2H), 3.44 (t, 2H), 2.57 (s, 3H), 2.04 (s, 3H), 0.82 (t, 2H), −0.07 (s, 9H). ^13^C NMR (50 MHz, DMSO) δ 169.2, 152.6, 149.4, 148.2, 146.1, 138.2, 135.7, 132.5, 131.5, 129.2, 129.0, 127.7, 124.2, 119.4, 113.5, 73.5, 65.6, 23.8, 17.2, 15.4, −1.5. TLC‐MS (ESI): calcd. *m/z* 499.17 for C_23_H_29_N_5_O_4_SSi, found 521.7 [M+Na]^+^.

*N*‐(4‐(4‐(2‐Aminophenyl)−2‐(methylthio)−1‐((2‐(trimethylsilyl)ethoxy)methyl)−1*H*‐imidazol‐5‐yl)pyridin‐2‐yl)acetamide (**S14**): To begin, 370 mg (0.74 mmol) **S13** and 242 mg (3.70 mmol, 5.0 eq.) zinc dust were suspended in 6 mL of MeOH (0.125 M). Under vigorous stirring, 233 mg (3.70 mmol, 5.0 eq.) of ammonium formate was added in one portion. The mixture was stirred for 1 h at ambient temperature, whereupon DCM was added, and the mixture was filtered over celite. The filtrate was washed with saturated aqueous NH_4_Cl solution, and the combined organic layers were dried over Na_2_SO_4_, filtered, and volatiles were removed in vacuo. The product was obtained in 94% yield (327 mg, 0.70 mmol) as a pale yellow solid. ^1^H NMR (200 MHz, DMSO) *δ* 10.57 (s, 1H), 8.28 (d, *J* = 5.1 Hz, 1H), 8.08 (s, 1H), 6.98–6.86 (m, 2H), 6.73–6.60 (m, 2H), 6.31 (t, *J* = 7.3 Hz, 1H), 5.64 (s, 2H), 5.17 (s, 2H), 3.46–3.36 (m, 2H), 2.64 (s, 3H), 2.06 (s, 3H), 0.86–0.75 (m, 2H), −0.08 (s, 9H). ^13^C NMR (50 MHz, DMSO) *δ* 169.2, 152.6, 148.2, 146.8, 144.6, 139.7, 138.9, 135.0, 129.4, 128.2, 128.0, 120.3, 116.4, 115.6, 114.0, 65.6, 25.0, 23.9, 17.2, 15.5, −1.5. TLC‐MS (ESI): calcd. *m/z* 469.20 for C_23_H_31_N_5_O_2_SSi, found 492.1 [M+Na]^+^.

*N*‐(4‐(4‐(3‐Aminophenyl)−2‐(methylthio)−1*H*‐imidazol‐5‐yl)pyridin‐2‐yl)acetamide (**1**): Prepared as previously described [39].

*N*‐(3‐(5‐(2‐Acetamidopyridin‐4‐yl)−2‐(methylthio)−1*H*‐imidazol‐4‐yl)phenyl)benzamide (**2**): The precursor of the title compound was synthesized from 40 mg (0.085 mmol) **S1** and 11 mg (0.085 mmol, 1 eq.) benzoic acid diluted in dry DCM (0.03 M). 48 mg (0.13 mmol, 1.5 eq.) HATU and 44 µl (0.26 mmol, 3.0 eq.) DIPEA were added, and the mixture was stirred overnight at ambient temperature. After complete consumption, celite was added and volatiles were removed in vacuo. Flash chromatographic purification (SiO_2_, *n‐*hexane/EtOAc 1:2) yielded 88% (43 mg, 0.075 mmol) of the precursor of the title compound as a yellow solid. TLC‐MS (ESI): calcd. *m*/*z* 573.22 for C_30_H_35_N_5_O_3_SSi, found 573.3 [M + H]^+^. The title compound was synthesized from 43 mg (0.075 mmol) of the above‐described precursor dissolved in a mixture of 20% v/v TFA in DCM (0.3 M). The mixture was stirred at ambient temperature overnight. After complete consumption, the mixture was poured into saturated aqueous NaHCO_3_ solution and vigorously stirred for 15 min. The aqueous phase was extracted with ethyl acetate three times. The combined organic layers were dried over Na_2_SO_4_, filtered, and evaporated to dryness. Flash chromatographic purification (SiO_2_, DCM/MeOH 9:1) yielded 90% (30 mg, 0.068 mmol) of the product as an off‐white solid. ^1^H NMR (200 MHz, DMSO) *δ* 12.73 (s, 1H), 10.65–10.13 (m, 2H), 8.41–8.20 (m, 1H), 8.09–7.80 (m, 4H), 7.69–7.32 (m, 4H), 7.24–6.96 (m, 2H), 2.63 (s, 3H), 2.05 (s, 3H). ^13^C NMR (50 MHz, DMSO) *δ* 169.0, 165.7, 152.5, 147.6, 143.7, 142.2, 139.6, 134.9, 134.3, 134.2, 131.6, 131.1, 130.6, 129.2, 128.4, 127.7, 123.8, 120.1, 116.5, 110.5, 23.9, 15.1. TLC‐MS (ESI): calcd. *m*/*z* 443.14 for C_24_H_21_N_5_O_2_S, found 444.2 [M + H]^+^. HPLC: tr = 6.00 min (99.7% purity).

*N*‐(3‐(5‐(2‐Acetamidopyridin‐4‐yl)−2‐(methylthio)−1*H*‐imidazol‐4‐yl)phenyl)−4‐fluoro benzamide (**3**): The precursor of the title compound was synthesized according to General Procedure A from 70 mg (0.15 mmol) **S1**, 27 mg (0.19 mmol, 1.3 eq.) 4‐fluorobenzoic acid, 101 mg (0.19 mmol, 1.3 eq.) PyBOP, and 77 µL (0.45 mmol, 3.0 eq.) DIPEA. Flash chromatographic purification (SiO_2_, *n‐*hexane/EtOAc 4:6) yielded 77% (68 mg, 0.12 mmol) of the product as a white solid. ^1^H NMR (200 MHz, CDCl_3_ + MeOD) *δ* 9.81–9.62 (m, 2H), 8.36–8.23 (m, 2H), 7.99 (dd, *J* = 8.6, 5.4 Hz, 2H), 7.88 (d, *J* = 8.3 Hz, 1H), 7.67 (s, 1H), 7.26–7.08 (m, 4H), 7.07–6.98 (m, 1H), 5.31 (s, 2H), 3.57 (t, 2H), 2.73 (s, 3H), 2.21 (s, 3H), 0.97 (t, 2H), 0.02 (s, 9H). TLC‐MS (ESI + ): calcd. *m/z* 591.21 for C_30_H_34_FN_5_O_3_SSi, found 613.8 [M+Na]^+^. The title compound was synthesized according to General Procedure B2 from 68 mg (0.12 mmol) of the above‐described precursor. Flash chromatographic purification (SiO_2_, DCM/MeOH 9:1) yielded 60% (32 mg, 0.07 mmol) of the product as an off‐white solid. ^1^H NMR (200 MHz, DMSO) *δ* 12.87–12.65 (m, 1H), 10.54–10.24 (m, 2H), 8.38 (s, 1H), 8.24–7.98 (m, 3H), 7.94–7.71 (m, 2H), 7.49–7.29 (m, 3H), 7.25–7.11 (m, 1H), 7.10–6.97 (m, 1H), 2.62 (s, 3H), 2.12–1.98 (m, 3H). ^13^C NMR (50 MHz, DMSO) *δ* 169.0, 164.6, 152.4, 147.5, 143.7, 142.2, 139.5, 134.3, 131.3, 131.1, 130.6, 130.5, 130.3, 129.2, 123.9, 120.2, 116.4, 115.6, 115.1, 110.5, 23.9, 15.1. HRMS (ESI): exact mass calcd. for C_24_H_21_FN_5_O_2_S [M + H]^+^: 462.13945, found: 462.14009. HPLC: tr = 6.61 min (99.8% purity).

*N*‐(3‐(5‐(2‐Acetamidopyridin‐4‐yl)−2‐(methylthio)−1*H*‐imidazol‐4‐yl)phenyl)−3‐fluoro benzamide (**4**): The precursor of the title compound was synthesized according to General Procedure A from 70 mg (0.15 mmol) **S1**, 27 mg (0.19 mmol, 1.3 eq.) 3 fluorobenzoic acid, 101 mg (0.19 mmol, 1.3 eq.) PyBOP, and 77 µL (0.45 mmol, 3.0 eq.) DIPEA. Flash chromatographic purification (SiO_2_, n‐hexane/EtOAc 4:6) yielded 92% (82 mg, 0.14 mmol) of the product as a white solid. ^1^H NMR (200 MHz, CDCl_3_ + MeOD) *δ* 9.78 (s, 1H), 9.60 (s, 1H), 8.37–8.18 (m, 2H), 7.94–7.84 (m, 1H), 7.80–7.61 (m, 3H), 7.52–7.38 (m, 1H), 7.28–7.14 (m, 2H), 7.11–6.96 (m, 2H), 5.28 (s, 2H), 3.66–3.45 (m, 2H), 2.71 (s, 3H), 2.20 (s, 3H), 1.00–0.86 (m, 2H), −0.00 (s, 9H). TLC‐MS (ESI + ): calcd. *m/z* 591.21 for C_30_H_34_FN_5_O_3_SSi, found 613.8 [M+Na]^+^. The title compound was synthesized according to General Procedure B1 from 80 mg (0.14 mmol) of the above‐described precursor. Flash chromatographic purification (SiO_2_, DCM/MeOH 9:1) yielded 41% (26 mg, 0.06 mmol) of the product as an off‐white solid. ^1^H NMR (200 MHz, DMSO) *δ* 12.76 (s, 1H), 10.49–10.26 (m, 2H), 8.42–8.23 (m, 1H), 8.22–8.07 (m, 1H), 7.93 (s, 1H), 7.87–7.71 (m, 3H), 7.66–7.39 (m, 3H), 7.22–7.14 (m, 1H), 7.10–7.01 (m, 1H), 2.63 (s, 3H), 2.05 (s, 3H). ^13^C NMR (101 MHz, DMSO) δ 169.6, 164.8, 163.6, 161.2, 152.8, 148.1, 148.0, 143.1, 139.6, 137.6, 131.1, 131.0, 129.5, 124.3, 120.6, 120.4, 119.1, 118.9, 117.2, 115.0, 114.8, 111.2, 24.3, 15.6. HRMS (ESI): exact mass calcd. for C_24_H_21_FN_5_O_2_S [M + H]^+^: 462.13945, found: 462.14022. HPLC: tr = 6.99 min (97.8% purity).

*N*‐(3‐(5‐(2‐Acetamidopyridin‐4‐yl)−2‐(methylthio)−1*H*‐imidazol‐4‐yl)phenyl)−3,5‐difluorobenzamide (**5**): The precursor of the title compound was synthesized according to General Procedure A from 80 mg (0.17 mmol) **S1**, 32 mg (0.20 mmol, 1.2 eq.) 3,5‐difluorobenzoic acid, 106 mg (0.20 mmol, 1.2 eq.) PyBOP, and 45 µl (0.26 mmol, 1.5 eq.) DIPEA. Flash chromatographic purification (SiO_2_, n‐hexane/EtOAc 50:50) yielded 84% (87 mg, 0.14 mmol) of the product as a white solid. ^1^H NMR (200 MHz, CDCl_3_) *δ* 9.57 (s, 1H), 9.01 (s, 1H), 8.29–8.20 (m, 2H), 7.94–7.82 (m, 1H), 7.71 (s, 1H), 7.42–7.34 (m, 2H), 7.23–7.11 (m, 3H), 6.88 (t, *J* = 8.6 Hz, 1H), 5.15 (s, 2H), 3.59–3.48 (m, 2H), 2.66 (s, 3H), 2.14 (s, 3H), 0.96–0.86 (m, 2H), −0.03 (s, 9H). The title compound was synthesized according to General Procedure B2 from 87 mg (0.14 mmol) of the above‐described precursor. Flash chromatographic purification (SiO_2_, DCM/MeOH 1%–10%) yielded 86% (51 mg, 0.12 mmol) of the product as an off‐white solid. ^1^H NMR (200 MHz, DMSO) *δ* 12.75 (s, 1H), 10.61–10.14 (m, 2H), 8.49–8.25 (m, 1H), 8.23–8.07 (m, 1H), 8.00–7.77 (m, 2H), 7.76–7.61 (m, 2H), 7.59–7.35 (m, 2H), 7.31–7.13 (m, 1H), 7.13–6.99 (m, 1H), 2.63 (s, 3H), 2.05 (s, 3H). HRMS (ESI): exact mass calcd. for C_24_H_20_F_2_N_5_O_2_S [M + H]^+^: 480.13003, found: 480.13039. HPLC: tr = 6.85 min (98.8% purity).

*N*‐(3‐(5‐(2‐Acetamidopyridin‐4‐yl)−2‐(methylthio)−1*H*‐imidazol‐4‐yl)phenyl)−2,6‐difluorobenzamide (**6**): The precursor of the title compound was synthesized from 43 mg (0.092 mmol) **S1** and 15 mg (0.092 mmol, 1 eq.) 2,6‐difluorobenzoic acid diluted in dry DCM (0.03 M). 52 mg (0.14 mmol, 1.5 eq.) HATU and 48 µl (0.28 mmol, 3.0 eq.) of DIPEA were added, and the mixture was stirred overnight at ambient temperature. After complete consumption, celite was added, and volatiles were removed in vacuo. Flash chromatographic purification (SiO_2_, *n‐*hexane/EtOAc 1:2) yielded 84% (46 mg, 0.075 mmol) of the precursor of the title compound as a yellow solid. TLC‐MS (ESI): calcd. *m*/*z* 609.20 for C_30_H_33_F_2_N_5_O_3_SSi, found 631.8 [M+Na]^+^. The title compound was synthesized from 46 mg (0.075 mmol) of the above‐described precursor dissolved in a mixture of 20% v/v TFA in DCM (0.3 M). The mixture was stirred at ambient temperature overnight. After complete consumption, the mixture was poured into saturated aqueous NaHCO_3_ solution and vigorously stirred for 15 min. The aqueous phase was extracted with ethyl acetate three times. The combined organic layers were dried over Na_2_SO_4_, filtered, and evaporated to dryness. Flash chromatographic purification (SiO_2_, DCM/MeOH 9:1) yielded 94% (34 mg, 0.071 mmol) of the product as an off‐white solid. ^1^H NMR (200 MHz, DMSO) *δ* 12.78 (s, 1H), 10.83 (s, 1H), 10.41 (s, 1H), 8.38–8.22 (m, 1H), 8.14 (d, *J* = 18.2 Hz, 1H), 7.89–7.38 (m, 4H), 7.29–7.16 (m, 3H), 7.10–7.00 (m, 1H), 2.62 (s, 3H), 2.04 (s, 3H). TLC‐MS (ESI): calcd. *m*/*z* 479.12 for C_24_H_19_F_2_N_5_O_2_S, found 504.3 [M+Na]^+^. HPLC: tr = 5.50 min (99.9% purity).

*N*‐(3‐(5‐(2‐Acetamidopyridin‐4‐yl)−2‐(methylthio)−1*H*‐imidazol‐4‐yl)phenyl)−2,4,6‐trifluoro benzamide (**7**): The precursor of the title compound was synthesized from 50 mg (0.11 mmol) **S1**, which was dissolved in 1 ml THF, and an aqueous 1 M NaHCO_3_ solution (0.21 mmol, 2.0 eq.) was added to the solution. The mixture was cooled down to 0°C, and 17 µL (0.133 mmol, 1.25 eq.) 2,4,6‐trifluorobenzoyl chloride was added dropwise. The mixture was stirred for 30 min at 0°C. Brine was added, and the aqueous phase was extracted several times with DCM. The combined organic layers were dried over Na_2_SO_4_, filtered, and volatiles were removed in vacuo. Flash chromatographic purification (SiO_2_, DCM/MeOH 1%–10%). TLC‐MS (ESI + ): calcd. *m/z* 627.19 for C_30_H_32_F_3_N_5_O_3_SSi, found 650.3 [M+Na]^+^. The product was directly used in the next step without further characterization. The title compound was synthesized according to General Procedure B1 from the product of the above‐described precursor. Flash chromatographic purification (SiO_2_, DCM/MeOH 1%–10%) yielded 77% (41 mg, 0.08 mmol) of the product over two steps as an off‐white solid. ^1^H NMR (200 MHz, DMSO) *δ* 12.77 (s, 1H), 11.10–10.16 (m, 2H), 8.45–8.05 (m, 2H), 7.89–7.60 (m, 2H), 7.53–7.27 (m, 3H), 7.24– 7.15 (m, 1H), 7.10–6.99 (m, 1H), 2.62 (s, 3H), 2.05 (s, 3H). HRMS (ESI): exact mass calcd. for C_24_H_19_F_3_N_5_O_2_S [M + H]^+^: 498.12061, found: 498.12106. HPLC: tr = 4.72 min (100% purity).

*N*‐(3‐(5‐(2‐Acetamidopyridin‐4‐yl)−2‐(methylthio)−1*H*‐imidazol‐4‐yl)phenyl)−3‐hydroxybenzamide (**8**): Prepared as previously described [6].

*N*‐(3‐(5‐(2‐Acetamidopyridin‐4‐yl)−2‐(methylthio)−1*H*‐imidazol4‐yl)phenyl)−2‐fluoro‐5‐hydroxybenzamide (**9**): Prepared as previously described [38].

*N*‐(3‐(5‐(2‐Acetamidopyridin‐4‐yl)−2‐(methylthio)−1*H*‐imidazol‐4‐yl)phenyl)nicotinamide (**10**): The precursor of the title compound was synthesized according to General Procedure A from **S1** (80 mg, 0.168 mmol, 1.0 eq.), 3‐pyridinylcarbonic acid (25 mg, 0.20 mmol, 1.2 eq.), PyBOP (104 mg, 0.20 mmol, 1.2 eq.), and DIPEA (44 µl, 0.25 mmol, 1.5 eq.) dissolved in 4 mL DMF. The crude mixture was purified by flash chromatography (SiO_2_, DCM/MeOH 5%) to give 82 mg of the pure product as a pale‐yellow solid in 85% yield. ^1^H NMR (200 MHz, CDCl_3_) *δ* 9.56 (s, 1H), 9.42 (s, 1H), 9.06 (s, 1H), 8.79–8.56 (m, 1H), 8.39–8.07 (m, 3H), 8.02–7.74 (m, 2H), 7.50–7.01 (m, 4H), 5.17 (s, 2H), 3.53 (t, *J* = 8.3 Hz, 2H), 2.69 (s, 3H), 2.15 (s, 3H), 1.00–0.84 (m, 2H), −0.02 (s, 9H). ^13^C NMR (50 MHz, CDCl_3_) *δ* 169.6, 164.2, 151.8, 148.5, 147.7, 146.0, 140.3, 139.9, 138.1, 135.4, 134.0, 130.9, 128.9, 127.8, 124.0, 123.2, 121.3, 119.8, 119.6, 116.4, 72.9, 66.4, 46.2, 26.2, 16.2, −1.5. The title compound was prepared from 82 mg (0.14 mmol) of the above‐described precursor according to general procedure B2. The crude product was purified by flash chromatography (SiO_2_, DCM/MeOH 10%). 49 mg (0.11 mmol) of the pure product was obtained as a white solid in 77% yield. ^1^H NMR (200 MHz, DMSO) *δ* 12.74 (s, 1H), 10.53 (s, 1H), 10.41 (s, 1H), 9.12 (s, 1H), 8.87–8.74 (m, 1H), 8.43–8.27 (m, 2H), 8.18 (d, *J* = 5.3 Hz, 1H), 7.96 (s, 1H), 7.86 (d, *J* = 8.3 Hz, 1H), 7.66–7.55 (m, 1H), 7.50–7.36 (m, 1H), 7.21 (d, *J* = 7.5 Hz, 1H), 7.10 (d, *J* = 5.2 Hz, 1H), 2.66 (s, 3H), 2.07 (s, 3H). ^13^C NMR (50 MHz, DMSO) δ 169.4, 164.5, 152.9, 152.5, 149.1, 148.1, 139.6, 135.8, 130.9, 129.4, 123.9, 120.4, 117.1, 111.0, 24.3, 15.5. ASAP‐MS (APCI): calcd. *m*/*z* 444.14 for C_23_H_20_N_6_O_2_S, found 445.0 [M + H]^+^. HPLC: tr = 4.34 min (97.2% purity).

*N*‐(3‐(5‐(2‐Acetamidopyridin‐4‐yl)−2‐(methylthio)−1*H*‐imidazol‐4‐yl)phenyl)picolinamide (**11**): The precursor of the title compound was synthesized according to General Procedure A from **S1** (80 mg, 0.168 mmol, 1.0 eq.), 2‐pyridinylcarclkdmclkdcbonic acid (25 mg, 0.20 mmol, 1.2 eq.), PyBOP (104 mg, 0.20 mmol, 1.2 eq.), and DIPEA (44 µL, 0.25 mmol, 1.5 eq.) dissolved in 4 mL DMF. The crude mixture was purified by flash chromatography (SiO_2_, DCM/MeOH 5%) to give 61 mg of the pure product as a pale‐yellow solid in 63% yield. ^1^H NMR (200 MHz, CDCl_3_) *δ* 10.05 (s, 1H), 9.33 (s, 1H), 8.60 (d, *J* = 4.7 Hz, 1H), 8.38 (s, 1H), 8.28 (d, *J* = 6.5 Hz, 2H), 7.97–7.85 (m, 3H), 7.54–7.38 (m, 1H), 7.35–7.17 (m, 1H), 7.17–7.04 (m, 2H), 5.27 (s, 2H), 3.63–3.49 (m, 2H), 2.77 (s, 3H), 2.21 (s, 3H), 0.95 (dd, *J* = 9.3, 7.4 Hz, 2H), −0.00 (s, 9H). ^13^C NMR (50 MHz, CDCl_3_) *δ* 168.9, 161.9, 152.2, 149.8, 147.9, 147.8, 145.9, 140.8, 139.9, 137.8, 137.5, 134.4, 128.9, 127.8, 126.3, 123.4, 122.3, 121.4, 118.8, 118.7, 115.3, 72.9, 66.3, 24.5, 17.7, 16.4, −1.5, −1.6. The title compound was prepared from 61 mg (0.106 mmol) of the above‐described precursor according to general procedure B2. The crude product was purified by flash chromatography (SiO_2_, DCM/MeOH 10%). 38 mg (0.085 mmol) of the pure product was obtained as a white solid in 81% yield. ^1^H NMR (400 MHz, DMSO) *δ* 12.84–12.57 (m, 1H), 10.84–10.26 (m, 2H), 8.88–8.61 (m, 1H), 8.40 (s, 1H), 8.26–8.03 (m, 4H), 8.01–7.80 (m, 1H), 7.72–7.63 (m, 1H), 7.49–7.28 (m, 1H), 7.24–7.16 (m, 1H), 7.12–7.02 (m, 1H), 2.64 (s, 3H), 2.05 (s, 3H). ^13^C NMR (101 MHz, DMSO) *δ* 169.4, 163.1, 152.9, 150.3, 148.9, 148.0, 144.3, 142.7, 139.2, 138.6, 134.9, 131.5, 131.2, 129.7, 127.4, 124.6, 122.9, 120.7, 120.6, 117.0, 111.1, 24.3, 15.6. ASAP‐MS (APCI): calcd. *m*/*z* 444.14 for C_23_H_20_N_6_O_2_S, found 444.9 [M + H]^+^. HPLC: tr = 5.46 min (95.6% purity).

*N*‐(3‐(5‐(2‐Acetamidopyridin‐4‐yl)−2‐(methylthio)−1*H*‐imidazol‐4‐yl)phenyl)isonicotinamide (**12**): The precursor of the title compound was synthesized according to General Procedure A from **S1** (80 mg, 0.168 mmol, 1.0 eq.), 4‐pyridinylcarbonic acid (25 mg, 0.20 mmol, 1.2 eq.), PyBOP (104 mg, 0.20 mmol, 1.2 eq.), and DIPEA (44 µL, 0.25 mmol, 1.5 eq.) dissolved in 4 mL DMF. The crude mixture was purified by flash chromatography (SiO_2_, DCM/MeOH 5%) to give 104 mg of the pure product as a pale‐yellow solid in quantitative yield. ^1^H NMR (200 MHz, CDCl_3_) *δ* 9.84 (s, 1H), 9.62 (s, 1H), 8.61 (d, *J* = 4.9 Hz, 2H), 8.40–8.05 (m, 2H), 7.95–7.62 (m, 4H), 7.23–7.01 (m, 3H), 5.17 (s, 2H), 3.60–3.43 (m, 2H), 2.66 (s, 3H), 2.11 (s, 3H), 0.99–0.83 (m, 2H), −0.04 (s, 9H). ^13^C NMR (50 MHz, CDCl_3_) *δ* 169.5, 164.1, 152.0, 149.9, 147.8, 145.8, 142.3, 140.2, 140.0, 138.1, 134.1, 128.7, 127.8, 124.1, 121.6, 121.2, 120.0, 116.0, 72.9, 66.3, 24.3, −1.5. The title compound was prepared from 104 mg (0.18 mmol) of the above‐described precursor according to general procedure B2. The crude product was purified by flash chromatography (SiO_2_, DCM/MeOH 10%). A volume of 50 mg (0.11 mmol) of the pure product was obtained as a white solid in 63% yield. ^1^H NMR (200 MHz, DMSO) *δ* 12.78 (s, 1H), 10.47 (d, *J* = 43.4 Hz, 2H), 8.82 (s, 2H), 8.47–7.77 (m, 6H), 7.59–7.00 (m, 3H), 2.65 (s, 3H), 2.08 (s, 3H). ^13^C NMR (50 MHz, DMSO) *δ* 169.3, 164.5, 152.8, 150.7, 147.9, 144.1, 142.7, 142.2, 139.4, 134.8, 131.3, 131.1, 129.7, 124.8, 122.0, 120.7, 116.9, 110.9, 24.3, 15.5. TLC‐MS (ESI): calcd. *m*/*z* 444.14 for C_23_H_20_N_6_O_2_S, found 445.5 [M + H]^+^. HPLC: tr = 1.97 min (98.3% purity).

*N*‐(3‐(5‐(2‐Acetamidopyridin‐4‐yl)−2‐(methylthio)−1*H*‐imidazol‐4‐yl)phenyl)−1‐methyl‐1*H*‐indole‐4‐carboxamide (**13**): The precursor of the title compound was synthesized according to General Procedure A from 80 mg (0.17 mmol) **S1**, 36 mg (0.20 mmol, 1.2 eq.) 1‐methyl‐1*H*‐indole‐4‐carboxylic acid, 106 mg (0.20 mmol, 1.2 eq.) PyBOP, and 45 µL (0.26 mmol, 1.5 eq.) DIPEA. Flash chromatographic purification (SiO_2_, n‐hexane/EtOAc 5:5) yielded 80% (85 mg, 0.14 mmol) of the product as a white solid. ^1^H NMR (200 MHz, CDCl_3_) *δ* 9.32 (s, 1H), 8.43 (s, 1H), 8.39–8.30 (m, 2H), 7.99 (d, *J* = 7.6 Hz, 1H), 7.84 (s, 1H), 7.67 (d, *J* = 7.1 Hz, 1H), 7.55 (d, *J* = 8.2 Hz, 1H), 7.36 (d, *J* = 7.4 Hz, 2H), 7.31–7.27 (m, 2H), 7.21–7.16 (m, 1H), 6.97 (d, *J* = 3.0 Hz, 1H), 5.33 (s, 2H), 3.90 (s, 3H), 3.68–3.58 (m, 2H), 2.83 (s, 3H), 2.23 (s, 3H), 1.08–0.98 (m, 2H), 0.08 (s, 9H). The title compound was synthesized according to General Procedure B2 from 85 mg (0.14 mmol) of the above‐described precursor. Flash chromatographic purification (SiO_2_, DCM/MeOH 1% to 10%) yielded 67% (32 mg, 0.09 mmol) of the product as an off‐white solid. As mixture of tautomers: ^1^H NMR (200 MHz, DMSO) *δ* 12.90–12.59 (m, 1H), 10.52–10.13 (m, 2H), 8.44–8.09 (m, 2H), 8.03–7.77 (m, 2H), 7.66 (d, *J *= 8.0 Hz, 1H), 7.57 (d, *J *= 7.1 Hz, 1H), 7.51–7.35 (m, 2H), 7.27 (t, *J* = 7.7 Hz, 1H), 7.19–7.03 (m, 2H), 6.85–6.75 (m, 1H), 3.85 (s, 3H), 2.63 (s, 3H), 2.05 (s, 3H). HRMS (ESI): exact mass calcd. for C_27_H_25_N_6_O_2_S [M + H]^+^: 497.17542, found: 497.17577. HPLC: tr = 5.85 min (97.8% purity).

*N*‐(3‐(5‐(2‐Acetamidopyridin‐4‐yl)−2‐(methylthio)−1*H*‐imidazol‐4‐yl)phenyl)−1‐naphthamide (**14**): The precursor of the title compound was synthesized according to General Procedure A from 60 mg (0.13 mmol) **S1**, 33 mg (0.19 mmol, 1.5 eq.) 1‐naphthoic acid, 82 mg (0.26 mmol, 2.0 eq.) TBTU, and 53 µL (0.45 mmol, 3.0 eq.) TEA. Flash chromatographic purification (SiO_2_, DCM/MeOH 1%–10%). TLC‐MS (ESI + ): calcd. *m/z* 623.24 for C_34_H_37_N_5_O_3_SSi, found 646.1 [M+Na]^+^. The product was directly used in the next step without further characterization. The title compound was synthesized according to General Procedure B1 from the product of the above‐described precursor. Flash chromatographic purification (SiO_2_, DCM/MeOH 1%–10%) yielded 62% (40 mg, 0.08 mmol) of the product over two steps as an off‐white solid. ^1^H NMR (200 MHz, DMSO) *δ* 12.76 (s, 1H), 10.75–10.25 (m, 2H), 8.43–7.95 (m, 6H), 7.90–7.69 (m, 2H), 7.66–7.55 (m, 3H), 7.53–7.27 (m, 1H), 7.27–7.05 (m, 2H), 2.63 (s, 3H), 2.05 (s, 3H). HRMS (ESI): exact mass calcd. for C_28_H_24_N_5_O_2_S [M + H]^+^: 494.16452, found: 494.16477. HPLC: tr = 6.19 min (100% purity).

*N*‐(3‐(5‐(2‐Acetamidopyridin‐4‐yl)−2‐(methylthio)−1*H*‐imidazol‐4‐yl)phenyl)−2‐naphthamide (**15**): The precursor of the title compound was synthesized according to General Procedure A from 60 mg (0.13 mmol) **S1**, 33 mg (0.19 mmol, 1.5 eq.) 2‐naphthoic acid, 82 mg (0.26 mmol, 2.0 eq.) TBTU, and 53 µL (0.45 mmol, 3.0 eq.) TEA. Flash chromatographic purification (SiO_2_, DCM/MeOH 1%–10%). TLC‐MS (ESI + ): calcd. *m/z* 623.24 for C_34_H_37_N_5_O_3_SSi, found 646.1 [M+Na]^+^. The product was directly used in the next step without further characterization. The title compound was synthesized according to General Procedure B1 from the product of the above‐described precursor. Flash chromatographic purification (SiO_2_, DCM/MeOH 1%–10%) yielded 70% (45 mg, 0.09 mmol) of the product over two steps as an off‐white solid. As mixture of tautomers: ^1^H NMR (400 MHz, DMSO) *δ* 12.90–12.62 (m, 1H), 10.57–10.25 (m, 2H), 8.57 (s, 1H), 8.41–8.11 (m, 2H), 8.10–7.97 (m, 5H), 7.93–7.81 (m, 1H), 7.72–7.57 (m, 2H), 7.53–7.27 (m, 1H), 7.22–7.03 (m, 2H), 2.63 (s, 3H), 2.05 (s, 3H). HRMS (ESI): exact mass calcd. for C_28_H_24_N_5_O_2_S [M + H]^+^: 494.16452, found: 494.16497. HPLC: tr = 6.96 min (97.5% purity).

*N*‐(3‐(5‐(2‐Acetamidopyridin‐4‐yl)−2‐(methylthio)−1*H*‐imidazol‐4‐yl)phenyl)thiophene‐3‐carboxamide (**16**): The precursor of the title compound was synthesized from 41 mg (0.087 mmol) **S1** and 11 mg (0.087 mmol, 1 eq.) thiophene‐3‐carboxylic acid diluted in dry DCM (0.03 M). 50 mg (0.13 mmol, 1.5 eq.) HATU and 45 µL (0.26 mmol, 3.0 eq.) of DIPEA were added, and the mixture was stirred overnight at ambient temperature. After complete consumption, celite was added and volatiles were removed in vacuo. Flash chromatographic purification (SiO_2_, *n‐*hexane/EtOAc 1:2) yielded 62 mg (0.107 mmol) of the precursor of the title compound as a colorless oil with *N*‐hydroxypyridinotriazole as impurity. TLC‐MS (ESI): calcd. *m*/*z* 579.18 for C_28_H_33_N_5_O_3_S_2_Si, found 601.7 [M+Na]^+^. The title compound was synthesized from 60 mg (0.104 mmol) of the above‐described precursor dissolved in a mixture of 20% v/v TFA in DCM (0.3 M). The mixture was stirred at ambient temperature overnight. After complete consumption, the mixture was poured into saturated aqueous NaHCO_3_ solution and vigorously stirred for 15 min. The aqueous phase was extracted with ethyl acetate three times. The combined organic layers were dried over Na_2_SO_4_, filtered, and evaporated to dryness. Flash chromatographic purification (SiO_2_, DCM/MeOH 9:1) yielded 84% (38 mg, 0.085 mmol) of the product as an off‐white solid. ^1^H NMR (200 MHz, CDCl_3_ + *MeOD*) δ 8.42 (d, *J* = 13.9 Hz, 2H), 8.10–7.81 (m, 4H), 7.75–7.42 (m, 4H), 2.92 (s, 3H), 2.44 (s, 3H). TLC‐MS (ESI): calcd. *m*/*z* 449.10 for C_22_H_19_N_5_O_2_S_2_, found 450.3 [M + H]^+^. HPLC: tr = 5.68 min (99.7% purity).

*N*‐(3‐(5‐(2‐Acetamidopyridin‐4‐yl)−2‐(methylthio)−1*H*‐imidazol‐4‐yl)phenyl)thiophene‐2‐carboxamide (**17**). The precursor of the title compound was synthesized from 41 mg (0.087 mmol) **S1** and 11 mg (0.087 mmol, 1 eq.) thiophene‐2‐carboxylic acid diluted in dry DCM (0.03 M). 50 mg (0.13 mmol, 1.5 eq.) HATU and 45 µL (0.26 mmol, 3.0 eq.) DIPEA were added, and the mixture was stirred overnight at ambient temperature. After complete consumption, celite was added, and volatiles were removed in vacuo. Flash chromatographic purification (SiO_2_, *n‐*hexane/EtOAc 1:2) yielded 52% (26 mg, 0.045 mmol) of the precursor of the title compound as a colorless oil. ^1^H NMR (200 MHz, CDCl_3_) *δ* 9.30 (s, 1H), 8.41–8.31 (m, 2H), 8.26 (d, *J* = 5.3 Hz, 1H), 7.92–7.82 (m, 1H), 7.71 (d, *J* = 3.8 Hz, 2H), 7.61–7.47 (m, 1H), 7.25–7.07 (m, 4H), 5.24 (s, 2H), 3.65–3.51 (m, 2H), 2.75 (s, 3H), 2.23 (s, 3H), 1.02–0.95 (m, 2H), 0.02 (s, 9H). TLC‐MS (ESI): calcd. *m*/*z* 579.18 for C_28_H_33_N_5_O_3_S_2_Si, found 601.7 [M+Na]^+^. The title compound was synthesized from 26 mg (0.058 mmol) of the above‐described precursor dissolved in a mixture of 20% v/v TFA in DCM (0.3 M). The mixture was stirred at ambient temperature overnight. After complete consumption, the mixture was poured into saturated aqueous NaHCO_3_ solution and vigorously stirred for 15 min. The aqueous phase was extracted with ethyl acetate three times. The combined organic layers were dried over Na_2_SO_4_, filtered, and evaporated to dryness. Flash chromatographic purification (SiO_2_, DCM/MeOH 9:1) yielded 70% (14 mg, 0.031 mmol) of the product as an off‐white solid. ^1^H NMR (200 MHz, DMSO) *δ* 12.85–12.70 (m, 1H), 10.55–10.21 (m, 2H), 8.41–8.09 (m, 2H), 8.05–7.98 (m, 1H), 7.93–7.72 (m, 3H), 7.52–7.36 (m, 1H), 7.28–7.13 (m, 2H), 7.09–7.01 (m, 1H), 2.67–2.60 (m, 3H), 2.10–2.01 (m, 3H). TLC‐MS (ESI): calcd. *m*/*z* 449.10 for C_22_H_19_N_5_O_2_S_2_, found 450.3 [M + H]^+^. HPLC: tr = 4.27 min (94.7% purity).

*N*‐(4‐(4‐(3‐(Methylsulfonamido)phenyl)−2‐(methylthio)−1*H*‐imidazol‐5‐yl)pyridin‐2‐yl)acetamide (**18**): The title compound was synthesized from **S1** (88 mg, 0.19 mmol, 1.0 eq.) and NaHCO_3_ (32 mg, 0.376 mmol, 2.0 eq.) dissolved in THF/water (400 µL 1:1) and stirred at ambient temperature. Methane sulfonylchloride (17 µL, 0.225 mmol, 1.2 eq.) was added to the well‐stirred solution. The mixture was stirred for 4 h at ambient temperature until complete consumption of the starting material. Then, brine was added, followed by EtOAc. The organic layer was separated, dried over sodium sulfate, filtered, and the solvents were removed by rotary evaporation. The residue was dissolved in 5 mL DCM and 2.5 mL TFA. Stirring was continued for 24 h. The mixture was quenched by the careful addition of saturated NaHCO_3_ solution, and the product was extracted with EtOAc three times. The combined organic layers were dried over sodium sulfate, filtered, and the solvents were removed by rotary evaporation. The crude product was purified by flash chromatography (SiO_2_, EtOAc) to give the final compound in 51% yield (40 mg, 0.096 mmol) over two steps as an off‐white solid. ^1^H NMR (200 MHz, DMSO) *δ* 12.76 (s, 1H), 10.61–10.26 (m, 1H), 9.80 (s, 1H), 8.22 (d, *J* = 26.0 Hz, 2H), 7.53–7.06 (m, 5H), 2.96 (s, 3H), 2.64 (s, 3H), 2.06 (s, 3H). ASAP‐MS (APCI): calcd. *m*/*z* 417.50 for C_18_H_19_N_5_O_3_S_2_, found 417.8 [M + H]^+^. HPLC: tr = 3.25 min (100% purity).

*N*‐(4‐(2‐(Methylthio)−4‐(3‐(propylsulfonamido)phenyl)−1*H*‐imidazol‐5‐yl)pyridin‐2‐yl)acetamide (**19**): The title compound was synthesized from **S1** (55 mg, 0.12 mmol, 1.0 eq.) dissolved in 100 µL pyridine and stirred at ambient temperature. Propane sulfonylchloride (16 µL, 0.14 mmol, 1.2 eq.) was added to the well‐stirred solution. The mixture was stirred at ambient temperature until complete consumption of the starting material. Then, dry DCM (4 mL) was added, followed by 2 mL TFA. Stirring was continued for 24 h. The mixture was quenched by the careful addition of saturated NaHCO_3_ solution, and the product was extracted with EtOAc three times. The combined organic layers were dried over sodium sulfate, filtered, and the solvents were removed by rotary evaporation. The crude product was purified by flash chromatography (SiO_2_, EtOAc) to give the final compound in 37% yield (20 mg, 0.044 mmol) over two steps as an off‐white solid. ^1^H NMR (200 MHz, DMSO) *δ* 12.76 (s, 1H), 10.44 (s, 1H), 9.87 (s, 1H), 8.33–8.07 (m, 2H), 7.22 (ddd, *J* = 37.8, 25.1, 6.5 Hz, 5H), 3.04 (t, *J* = 7.6 Hz, 2H), 2.64 (s, 3H), 2.07 (s, 3H), 1.67 (h, *J* = 7.4 Hz, 2H), 0.94 (t, *J* = 7.4 Hz, 3H). ^13^C NMR (50 MHz, CDCl_3_) *δ* 169.4, 152.8, 148.1, 143.0, 139.1, 130.2, 123.8, 119.1, 117.2, 111.1, 52.6, 24.3, 17.2, 15.5, 12.9. ASAP‐MS (APCI): calcd. *m*/*z* 445.12 for C_20_H_23_N_5_O_3_S_2_, found 445.9 [M + H]^+^. HPLC: tr = 2.82 min (95.1% purity).

*N*‐(4‐(4‐(3‐(Cyclopropanesulfonamido)phenyl)−2‐(methylthio)−1*H*‐imidazol‐5‐yl)pyridin‐2‐yl)acetamide (**20**): The title compound was synthesized from **S1** (66 mg, 0.14 mmol, 1.0 eq.) dissolved in 200 µL DCM, followed by the addition of 17 µL pyridine (0.21 mmol, 1.5 eq.), and stirred at ambient temperature. Cyclopropane sulfonylchloride (17 µL, 0.17 mmol, 1.2 eq.) was added to the well‐stirred solution. The mixture was stirred at ambient temperature until complete consumption of the starting material. Then, dry DCM (4 mL) was added, followed by 2 mL TFA. Stirring was continued for 24 h. The mixture was quenched by the careful addition of saturated NaHCO_3_ solution, and the product was extracted with EtOAc three times. The combined organic layers were dried over sodium sulfate, filtered, and the solvents were removed by rotary evaporation. The crude product was purified by flash chromatography (SiO_2_, EtOAc) to yield the final compound in 50% yield (31 mg, 0.07 mmol) over two steps as an off‐white solid. ^1^H NMR (400 MHz, DMSO‐*d*_6_) *δ* 12.75 (d, *J* = 23.6 Hz, 1H), 10.41 (d, *J* = 55.5 Hz, 1H), 9.71 (d, *J* = 54.5 Hz, 1H), 8.40– 8.03 (m, 2H), 7.48–7.10 (m, 4H), 7.02 (d, *J* = 5.3 Hz, 1H), 2.62 (s, 3H), 2.04 (s, 3H), 1.09 (t, *J* = 7.0 Hz, 1H), 0.98–0.84 (m, 4H). ^13^C NMR (101 MHz, DMSO) *δ* 169.5, 153.0, 148.0, 144.3, 142.8, 135.2, 131.6, 131.0, 130.2, 124.3, 120.4, 117.2, 111.3, 65.3, 29.9, 24.3, 15.6, 5.4 ASAP‐MS (APCI): calcd. *m*/*z* 443.11 for C_20_H_21_N_5_O_3_S_2_, found 443.9 [M + H]^+^. HPLC: tr = 1.81 min (97.6% purity).

*N*‐(4‐(2‐(Methylthio)−4‐(3‐(phenylsulfonamido)phenyl)−1*H*‐imidazol‐5‐yl)pyridin‐2‐yl)acetamide (**21**): To begin, **S1** (64 mg, 0.14 mmol, 1.0 eq.) and NaHCO_3_ (23 mg, 0.27 mmol, 2.0 eq.) were dissolved in THF/water (400 µL 1:1) and stirred at ambient temperature. Benzene sulfonylchloride (21 µL, 0.16 mmol, 1.2 eq.) was added to the well‐stirred solution. The mixture was stirred at ambient temperature until complete consumption of the starting material (4 h). Then brine was added, followed by EtOAc. The organic layer was separated, dried over sodium sulfate, filtered, and the solvents were removed by rotary evaporation. The residue was dissolved in 5 mL DCM and 2.5 mL TFA. Stirring was continued for 24 h. The mixture was quenched by the careful addition of saturated NaHCO_3_ solution, and the product was extracted with EtOAc three times. The combined organic layers were dried over sodium sulfate, filtered, and the solvents were removed by rotary evaporation. The crude product was purified by flash chromatography (SiO_2_, EtOAc) to yield the final compound in 43% yield (28 mg, 0.06 mmol) over two steps as a white solid. ^1^H NMR (200 MHz, DMSO) *δ* 12.72 (s, 1H), 10.41 (d, *J* = 18.0 Hz, 2H), 8.41–8.03 (m, 2H), 7.73 (d, *J* = 7.4 Hz, 2H), 7.69–7.47 (m, 3H), 7.37–7.00 (m, 4H), 6.87 (d, *J* = 5.4 Hz, 1H), 2.62 (s, 3H), 2.10 (s, 3H). ^13^C NMR (50 MHz, DMSO) *δ* 169.4, 152.9, 148.0, 143.0, 140.0, 138.4, 133.3, 129.9, 129.6, 126.9, 124.1, 120.0, 117.1, 111.3, 24.3, 15.4. ASAP‐MS (APCI): calcd. *m*/*z* 479.11 for C_23_H_21_N_5_O_3_S_2_, found 479.8 [M + H]^+^. HPLC: tr = 1.80 min (97.7% purity).

*N*‐(5‐(5‐(2‐Acetamidopyridin‐4‐yl)−2‐(methylthio)−1*H*‐imidazol‐4‐yl)−2‐fluorophenyl) benzamide (**22**): The precursor of the title compound was synthesized according to General Procedure A from 60 mg (0.12 mmol) **S6**, 29 mg (0.18 mmol, 1.5 eq.) benzoic acid, 79 mg (0.25 mmol, 2.0 eq.) TBTU, and 51 µL (0.37 mmol, 3.0 eq.) TEA. Flash chromatographic purification (SiO_2_, DCM/MeOH 1%–10%). TLC‐MS (ESI + ): calcd. *m/z* 591.21 for C_30_H_34_FN_5_O_3_SSi, found 614.2 [M+Na]^+^. The product was directly used in the next step without further characterization. The title compound was synthesized according to General Procedure B1 from the product of the above‐described precursor. Flash chromatographic purification (SiO_2_, DCM/MeOH 1%–10%) yielded 35% (20 mg, 0.04 mmol) of the product over two steps as an off‐white solid. As mixture of tautomers: 1H NMR (400 MHz, DMSO) *δ* 12.89–12.69 (m, 1H), 10.55–10.32 (m, 1H), 10.26–10.08 (m, 1H), 8.37–8.11 (m, 2H), 8.00–7.93 (m, 2H), 7.82–7.71 (m, J = 6.3 Hz, 1H), 7.63–7.50 (m, 3H), 7.42–7.19 (m, 2H), 7.13–7.03 (m, 1H), 2.62 (s, 3H), 2.10–2.03 (m, 3H). HRMS (ESI): exact mass calcd. for C_24_H_21_FN_5_O_2_S [M + H]^+^: 462.13945, found: 462.14022. HPLC: tr = 5.15 min (97.8% purity).

*N*‐(5‐(5‐(2‐Acetamidopyridin‐4‐yl)−2‐(methylthio)−1*H*‐imidazol‐4‐yl)−2‐fluorophenyl)−2,6‐difluorobenzamide (**23**): The precursor of the title compound was synthesized according to General Procedure A from 60 mg (0.12 mmol) **S6**, 24 mg (0.15 mmol, 1.25 eq.) 2,6‐difluorobenzoic acid, 80 mg (0.15 mmol, 1.25 eq.) PyBOP, and 51 µL (0.37 mmol, 3.0 eq.) TEA. Flash chromatographic purification (SiO_2_, DCM/MeOH 1%–10%). TLC‐MS (ESI + ): calcd. *m/z* 627.19 for C_30_H_32_F_3_N_5_O_3_SSi, found 649.9 [M+Na]^+^. The product was directly used in the next step without further characterization. The title compound was synthesized according to General Procedure B1 from the product of the above‐described precursor. Flash chromatographic purification (SiO_2_, DCM/MeOH 1%–10%) yielded 34% (21 mg, 0.04 mmol) of the product over two steps as an off‐white solid. As mixture of tautomers: 1H NMR (400 MHz, DMSO) *δ* 12.94–12.66 (m, 1H), 10.86–10.57 (m, 1H), 10.52–10.26 (m, 1H), 8.38–8.10 (m, 2H), 8.08–7.96 (m, J = 6.2 Hz, 1H), 7.65–7.53 (m, 1H), 7.42–7.19 (m, 4H), 7.13–7.02 (m, J = 5.1 Hz, 1H), 2.63 (s, 3H), 2.13–2.00 (m, 3H). HRMS (ESI): exact mass calcd. for C_24_H_19_F_3_N_5_O_2_S [M + H]^+^: 498.12061, found: 498.12098. HPLC: tr = 4.28 min (94.1% purity at 254 nm, 94.5% at 230 nm).

*N*‐(5‐(5‐(2‐Acetamidopyridin‐4‐yl)−2‐(methylthio)−1*H*‐imidazol‐4‐yl)−2‐fluorophenyl)−3,5‐difluorobenzamide (**24**): The precursor of the title compound was synthesized according to General Procedure A from 60 mg (0.12 mmol) **S6**, 29 mg (0.18 mmol, 1.5 eq.) 3,5‐difluorobenzoic acid, 79 mg (0.25 mmol, 2.0 eq.) TBTU, and 51 µL (0.37 mmol, 3.0 eq.) TEA. Flash chromatographic purification (SiO_2_, DCM/MeOH 1%–10%). TLC‐MS (ESI + ): calcd. *m/z* 627.19 for C_30_H_32_F_3_N_5_O_3_SSi, found 650.1 [M+Na]^+^. The product was directly used in the next step without further characterization. The title compound was synthesized according to General Procedure B1 from the product of the above‐described precursor. Flash chromatographic purification (SiO_2_, DCM/MeOH 1% to 10%) yielded 50% (30 mg, 0.06 mmol) of the product over two steps as an off‐white solid. As mixture of tautomers: 1H NMR (200 MHz, DMSO) *δ* 12.98–12.56 (m, 1H), 10.56–10.25 (m, 2H), 8.37–8.08 (m, 2H), 7.82–7.48 (m, 4H), 7.45–7.19 (m, 2H), 7.13–7.01 (m, 1H), 2.62 (s, 3H), 2.06 (s, 3H). HRMS (ESI): exact mass calcd. for C_24_H_19_F_3_N_5_O_2_S [M + H]^+^: 498.12061, found: 498.12159. HPLC: tr = 6.76 min (100% purity).

*N*‐(2‐(5‐(2‐Acetamidopyridin‐4‐yl)−2‐(methylthio)−1*H*‐imidazol‐4‐yl)phenyl)benzamide (**25**): The precursor of the title compound was synthesized according to General Procedure A from 50 mg (0.13 mmol) **S14**, 17 mg (0.14 mmol, 1.3 eq.) benzoic acid, 72 mg (0.14 mmol, 1.3 eq.) PyBOP, and 45 µL (0.26 mmol, 3.0 eq.) DIPEA. Flash chromatographic purification (SiO_2_, *n*‐hexane/EtOAc 3:7) yielded 96% (59 mg, 0.13 mmol) of the product as an off‐white solid. TLC‐MS (ESI + ): calcd. *m*/*z* 573.22 for C_30_H_35_N_5_O_3_SSi, found 596.6 [M+Na]^+^. The title compound was synthesized according to General Procedure B2 from 59 mg (0.13 mmol) of the above‐described precursor. Flash chromatographic purification (SiO_2_, DCM/MeOH 1%–8%) yielded 70% (40 mg, 0.09 mmol) of the product as an off‐white solid. As mixture of tautomers: ^1^H NMR (200 MHz, DMSO) *δ* 13.36–12.47 (m, 1H), 11.55–10.40 (m, 1H), 10.36–9.27 (m, 1H), 8.52–8.11 (m, 2H), 8.06–7.74 (m, 2H), 7.64–7.18 (m, 6H), 7.10–6.39 (m, 2H), 2.60 (s, 3H), 2.05 (s, 3H). HRMS (ESI): exact mass calcd. for C_24_H_22_N_5_O_2_S [M + H]^+^: 444.14887, found: 444.14929. HPLC: t_r_ = 4.19 min (100% purity).

*N*‐(2‐(5‐(2‐Acetamidopyridin‐4‐yl)−2‐(methylthio)−1*H*‐imidazol‐4‐yl)phenyl)−4‐fluorobenzamide (**26**): The precursor of the title compound was synthesized according to General Procedure A from 58 mg (0.13 mmol) **S14**, 22 mg (0.16 mmol, 1.3 eq.) 4‐fluorobenzoic acid, 83 mg (0.16 mmol, 1.3 eq.) PyBOP, and 65 µL (0.37 mmol, 3.0 eq.) DIPEA. Flash chromatographic purification (SiO_2_, *n*‐hexane/EtOAc 3:7) yielded 86% (63 mg, 0.11 mmol) of the product as an off‐white solid. ^1^H NMR (200 MHz, CDCl_3_) *δ* 11.72 (s, 1H), 9.22–9.03 (m, 1H), 8.68–8.56 (m, 1H), 8.41 (s, 1H), 8.28–8.18 (m, 1H), 8.15–8.01 (m, 2H), 7.36–7.14 (m, 3H), 6.99–6.80 (m, 3H), 5.32 (s, 2H), 3.59–3.46 (m, 2H), 2.69 (s, 3H), 2.25 (s, 3H), 1.00–0.89 (m, 2H), −0.02 (s, 9H). TLC‐MS (ESI + ): calcd. *m*/*z* 591.21 for C_30_H_34_FN_5_O_3_SSi, found 614.1 [M+Na]^+^. The title compound was synthesized according to General Procedure B2 from 53 mg (0.09 mmol) of the above‐described precursor. Flash chromatographic purification (SiO_2_, DCM/MeOH 1%–6%) yielded 60% (25 mg, 0.05 mmol) of the product as an off‐white solid. As mixture of tautomers: ^1^H NMR (200 MHz, DMSO) *δ* 13.27–12.45 (m, 1H), 11.36–10.43 (m, 1H), 10.22–9.41 (m, 1H), 8.46–8.06 (m, 1H), 8.02–7.70 (m, 1H), 7.60–7.24 (m, 1H), 7.24–6.64 (m, 1H), 2.59 (s, 3H), 2.04 (s, 3H). HRMS (ESI): exact mass calcd. for C_24_H_21_FN_5_O_2_S [M + H]^+^: 462.13945, found: 462.14002. HPLC: *t*_r_ = 6.51 min (98.5% purity).

*N*‐(2‐(5‐(2‐Acetamidopyridin‐4‐yl)−2‐(methylthio)−1*H*‐imidazol‐4‐yl)phenyl)−2,6‐difluorobenzamide (**27**): The precursor of the title compound was synthesized according to General Procedure A from 40 mg (0.09 mmol) **S14**, 16 mg (0.10 mmol, 1.2 eq.) 2,6‐difluorobenzoic acid, 62 mg (0.12 mmol, 1.4 eq.) PyBOP, and 40 µL (0.26 mmol, 3.0 eq.) TEA. Flash chromatographic purification (SiO_2_, *n*‐hexane/EtOAc 35:65) yielded 69% (36 mg, 0.06 mmol) of the product as a pale‐yellow solid. ^1^H NMR (200 MHz, CDCl_3_) *δ* 11.70 (s, 1H), 9.10–8.89 (m, 1H), 8.72–8.61 (m, 1H), 8.48–8.34 (m, 1H), 8.29–8.18 (m, 1H), 7.51–7.24 (m, 2H), 7.06–6.82 (m, 5H), 5.23 (s, 2H), 3.60–3.46 (m, 2H), 2.35 (s, 3H), 2.25 (s, 3H), 1.00–0.89 (m, 2H), −0.01 (s, 9H). TLC‐MS (ESI + ): calcd. *m*/*z* 609.20 for C_30_H_33_F_2_N_5_O_3_SSi, found 632.7 [M+Na]^+^. The title compound was synthesized according to General Procedure B2 from 32 mg (0.05 mmol) of the above‐described precursor. Flash chromatographic purification (SiO_2_, DCM/MeOH 1%–10%) yielded 75% (19 mg, 0.04 mmol) of the product as an off‐white solid. ^1^H NMR (200 MHz, DMSO) *δ* 13.77–12.96 (m, 1H), 11.35 (s, 1H), 10.45 (s, 1H), 8.22–7.85 (m, 3H), 7.65–7.31 (m, 4H), 7.23–7.00 (m, 3H), 2.62 (s, 3H), 2.15 (s, 3H). HRMS (ESI): exact mass calcd. for C_24_H_20_F_2_N_5_O_2_S [M + H]^+^: 480.13003, found: 480.13067. HPLC: t_r_ = 3.45 min (98.9% purity).

*N*‐(2‐(5‐(2‐Acetamidopyridin‐4‐yl)−2‐(methylthio)−1*H*‐imidazol‐4‐yl)phenyl)−3‐hydroxybenzamide (**28**): The precursor of the title compound was synthesized according to General Procedure A from 70 mg (0.15 mmol) **S14**, 40 mg (0.22 mmol 1.5 eq.) 3‐acetoxybenzoic acid, 72 mg (0.22 mmol, 1.5 eq.) TBTU, and 62 µL (0.45 mmol, 3.0 eq.) TEA. Flash chromatographic purification (SiO_2_, *n*‐hexane/EtOAc 2:8) yielded 53% (50 mg, 0.08 mmol) of the product as a yellow solid. ^1^H NMR (200 MHz, CDCl_3_) *δ* 11.66 (s, 1H), 9.03–8.80 (m, 1H), 8.60 (d, *J* = 8.2 Hz, 1H), 8.42 (s, 1H), 8.27–8.18 (m, 1H), 8.04–7.90 (m, 1H), 7.81–7.71 (m, 1H), 7.54 (t, *J* = 7.9 Hz, 1H), 7.48–7.25 (m, 2H), 7.00–6.79 (m, 3H), 5.32 (s, 2H), 3.61–3.48 (m, 2H), 2.69 (s, 3H), 2.38 (s, 3H), 2.25 (s, 3H), 1.01–0.90 (m, 2H), −0.02 (s, 9H). TLC‐MS (ESI + ): calcd. *m*/*z* 631.23 for C_32_H_37_N_5_O_5_SSi, found 654.6 [M+Na]^+^. The title compound was synthesized according to General Procedure B2 from 45 mg (0.07 mmol) of the above‐described precursor. After stirring overnight under acidic conditions [identification of intermediate via TLC‐MS (ESI + ): calcd. *m/z* 501.15 for C_26_H_23_N_5_O_4_S, found 524.6 [M+Na]^+^], the residue was dissolved in MeOH, saturated aqueous NaHCO_3_ was added until precipitation was observed, and the mixture was stirred at ambient temperature for 1 h. The aqueous phase was extracted with DCM several times, and the combined organic layers were dried over Na_2_SO_4_, filtered, and volatiles were removed in vacuo. Flash chromatographic purification (SiO_2_, DCM/MeOH 1% to 10%) yielded 61% (20 mg, 0.04 mmol) of the product over two steps as an off‐white solid. As mixture of tautomers: ^1^H NMR (200 MHz, DMSO) *δ* 13.45–12.30 (m, 1H), 11.31 (s, 1H), 10.45 (s, 1H), 9.72 (s, 1H), 8.50–7.71 (m, 3H), 7.45–7.06 (m, 5H), 7.02–6.73 (m, 3H), 2.61 (s, 3H), 2.06 (s, 3H). HRMS (ESI): exact mass calcd. for C_24_H_22_N_5_O_3_S [M + H]^+^: 460.14379, found: 460.14451. HPLC: *t*_r_ = 2.93 min (98.1% purity).

*N*‐(2‐(5‐(2‐Acetamidopyridin‐4‐yl)−2‐(methylthio)−1*H*‐imidazol‐4‐yl)phenyl)nicotinamide (**29**): The precursor of the title compound was synthesized according to General Procedure A from 50 mg (0.11 mmol) **S14**, 17 mg (0.14 mmol, 1.3 eq.) nicotinic acid, 72 mg (0.14 mmol, 1.3 eq.) PyBOP, and 45 µL (0.32 mmol, 3.0 eq.) TEA. Flash chromatographic purification (SiO_2_, *n*‐hexane/EtOAc 2:8) yielded 73% (45 mg, 0.08 mmol) of the product as a white solid. ^1^H NMR (200 MHz, CDCl_3_) *δ* 11.74 (s, 1H), 9.32 (s, 1H), 9.10 (s, 1H), 8.73–8.58 (m, 1H), 8.54–8.13 (m, 4H), 7.89–7.28 (m, 2H), 7.19 (t, *J* = 7.1 Hz, 1H), 6.96–6.70 (m, 3H), 5.17 (s, 2H), 3.48–3.35 (m, 2H), 2.55 (s, 3H), 2.10 (s, 3H), 0.86–0.73 (m, 2H), −0.16 (s, 9H). TLC‐MS (ESI + ): calcd. *m*/*z* 574.22 for C_29_H_34_N_6_O_3_SSi, found 597.3 [M+Na]^+^. The title compound was synthesized according to General Procedure B2 from 45 mg (0.08 mmol) of the above‐described precursor. Flash chromatographic purification (SiO_2_, *n*‐hexane/EtOAc/MeOH 5:90:5) yielded 45% (15 mg, 0.04 mmol) of the product as an off‐white solid. ^1^H NMR (200 MHz, DMSO) δ 12.77 (s, 1H), 11.31–9.40 (m, 2H), 9.04–8.42 (m, 2H), 8.32–7.65 (m, 4H), 7.59– 7.04 (m, 4H), 6.82 (s, 1H), 2.60 (s, 3H), 2.05 (s, 3H). HRMS (ESI): exact mass calcd. for C_23_H_21_N_6_O_2_S [M + H]^+^: 445.14412, found: 445.14437. HPLC: t_r_ = 2.67 min (97.4% purity).

*N*‐(2‐(5‐(2‐Acetamidopyridin‐4‐yl)−2‐(methylthio)−1*H*‐imidazol‐4‐yl)phenyl)picolinamide (**30**): The precursor of the title compound was synthesized according to General Procedure A from 50 mg (0.11 mmol) **S14**, 20 mg (0.16 mmol, 1.5 eq.) picolinic acid, 83 mg (0.16 mmol, 1.5 eq.) PyBOP, and 45 µL (0.32 mmol, 3.0 eq.) TEA. Flash chromatographic purification (SiO_2_, *n*‐hexane/EtOAc 2:8) yielded 82% (50 mg, 0.09 mmol) of the product as a white solid. ^1^H NMR (200 MHz, CDCl_3_) *δ* 12.11 (s, 1H), 9.40 (s, 1H), 8.78–8.52 (m, 1H), 8.39 (s, 1H), 8.31 (d, *J* = 7.8 Hz, 1H), 8.12 (d, *J* = 5.1 Hz, 1H), 7.87 (t, *J* = 7.5 Hz, 1H), 7.51–7.39 (m, 1H), 7.37–7.26 (m, 1H), 7.09–6.82 (m, 3H), 5.30 (s, 2H), 3.72–3.52 (m, 2H), 2.97 (s, 3H), 2.19 (s, 3H), 1.03–0.91 (m, 2H), 0.02 (s, 9H). TLC‐MS (ESI + ): calcd. *m*/*z* 574.22 for C_29_H_34_N_6_O_3_SSi, found 597.5 [M+Na]^+^. The title compound was synthesized according to General Procedure B2 from 50 mg (0.09 mmol) of the above‐described precursor. Flash chromatographic purification (SiO_2_, DCM/MeOH 1%–10%) yielded 75% (29 mg, 0.07 mmol) of the product as an off‐white solid. ^1^H NMR (200 MHz, DMSO) *δ* 13.07 (s, 1H), 11.92 (s, 1H), 10.40 (s, 1H), 8.70–8.38 (m, 2H), 8.29–7.91 (m, 4H), 7.68–7.55 (m, 1H), 7.51–7.03 (m, 3H), 6.96–6.77 (m, 1H), 2.76 (s, 3H), 2.04 (s, 3H). HRMS (ESI): exact mass calcd. for C_23_H_21_N_6_O_2_S [M + H]^+^: 445.14412, found: 445.14489. HPLC: *t*_r_ = 4.35 min (98.3% purity).

*N*‐(2‐(5‐(2‐Acetamidopyridin‐4‐yl)−2‐(methylthio)−1*H*‐imidazol‐4‐yl)phenyl)isonicotinamide (**31**): The precursor of the title compound was synthesized according to General Procedure A from 50 mg (0.11 mmol) **S14**, 17 mg (0.14 mmol, 1.3 eq.) isonicotinic acid, 72 mg (0.14 mmol, 1.3 eq.) PyBOP, and 45 µL (0.32 mmol, 3.0 eq.) TEA. Flash chromatographic purification (SiO_2_, *n*‐hexane/EtOAc 2:8) yielded 82% (50 mg, 0.09 mmol) of the product as a white solid. ^1^H NMR (200 MHz, CDCl_3_) *δ* 11.87 (s, 1H), 9.38 (s, 1H), 8.63 (d, *J* = 4.2 Hz, 2H), 8.35 (d, *J* = 8.2 Hz, 1H), 8.15–8.10 (m, 1H), 7.87–7.74 (m, 3H), 7.15 (t, *J* = 7.0 Hz, 1H), 6.96–6.61 (m, 3H), 5.15 (s, 2H), 3.43–3.29 (m, 2H), 2.44 (s, 3H), 2.05 (s, 3H), 0.81–0.70 (m, 2H), −0.19 (s, 9H). TLC‐MS (ESI + ): calcd. *m*/*z* 574.22 for C_29_H_34_N_6_O_3_SSi, found 597.3 [M+Na]^+^. The title compound was synthesized according to General Procedure B2 from 50 mg (0.09 mmol) of the above‐described precursor. Flash chromatographic purification (SiO_2_, DCM/MeOH 1% to 9%) yielded 41% (16 mg, 0.04 mmol) of the product as an off‐white solid. ^1^H NMR (200 MHz, DMSO) *δ* 12.69 (s, 1H), 10.66–9.39 (m, 2H), 8.77–8.55 (m, 2H), 8.30–7.89 (m, 3H), 7.58–7.12 (m, 5H), 6.80 (s, 1H), 2.59 (s, 3H), 2.04 (s, 3H). HRMS (ESI): exact mass calcd. for C_23_H_21_N_6_O_2_S [M + H]^+^: 445.14412, found: 445.14497. HPLC: *t*_r_ = 2.49 min (100% purity).

### 5.2 Pharmacological/Biological Assays

#### 5.2.1 Protein Expression and Purification

The EGFR kinase domain (residues 696–1022) was cloned into pTriEx with an N‐terminal 6xHis‐glutathione S‐transferase (GST) fusion tag followed by a TEV protease cleavage site. EGFR L858R, L858R/T790M, and T790M/V948R were expressed after baculoviral infection in SF9. Briefly, cells were pelleted and resuspended in lysis buffer composed of 50 mM Tris pH 8.0, 500 mM NaCl, 1 mM tris(2‐carboxyethyl) phosphine (TCEP), and 5% glycerol. Cells were lysed via sonication before ultracentrifugation at > 200,000*g* for 1 h. Imidazole pH 8.0 was added to the supernatant for a final concentration of 40 mM and flowed through a column containing Ni‐NTA agarose beads. The resin was washed with lysis buffer supplemented with 40 mM imidazole and eluted with lysis buffer containing 200 mM imidazole. The eluted EGFR kinase domain was dialyzed overnight in the presence of 5% (w/w) TEV protease against dialysis buffer containing 50 mM Tris pH 8.0, 500 mM NaCl, 1 mM TCEP, and 5% glycerol. The cleaved protein was passed through Ni‐NTA resin to remove the 6xHis‐GST fusion protein and TEV before size exclusion chromatography on a prep‐grade Superdex S200 column in 50 mM Tris pH 8.0, 500 mM NaCl, 1 mM TCEP, and 5% glycerol. Fractions containing EGFR kinase of ≥ 95% purity, as assessed by Coomassie‐stained SDS‐PAGE, were concentrated to approximately 4 mg/mL as determined by Bradford assay or absorbance.

#### 5.2.2 HTRF Assays

Biochemical assays for EGFR domains were carried out using an HTRF KinEASE‐TK (Cisbio) assay, as described previously [38]. Assays were optimized for ATP concentration of 100 µM with enzyme concentrations L858R 0.1 nM and L858R/T790M at 0.02 nM. Inhibitor compounds in DMSO were dispensed directly in 384‐well plates with the D300 digital dispenser (Hewlett Packard), followed immediately by the addition of aqueous buffered solutions using the Multidrop Combi Reagent Dispenser (Thermo Fischer). Compound IC_50_ values were determined by 11‐point inhibition curves (from 1.0 to 0.00130 μM) in triplicate. The data were graphically displayed using GraphPad Prism version 7.0 (GraphPad software). The curves were fitted using a nonlinear regression model with a sigmoidal dose response. Well‐characterized controls are included in each experiment (JBJ‐04‐125‐02, osimertinib) to confirm reliability, and IC_50_ values have been reproduced to ensure consistency.

#### 5.2.3 Western Blotting

H1975, H3255, and HCC827 cells were treated for 6 h with concentrations and inhibitors indicated in the figure legend. Cells were collected and lysed with lysis buffer (5 M NaCl, 1 M TRIS pH 8.0, 10% SDS, 10% Triton X‐100, and a tablet of protease inhibitors). Cell lysates were prepared using SDS‐PAGE loading buffer and subjected to SDS‐PAGE. They were then transferred to nitrocellulose membranes and blocked with 5% dry milk in TBS. Membranes were then probed with different primary and secondary antibodies. Primary antibodies used, phospho‐EGFR (Tyr1068; #2234, 1:1,000) and EGFR (#4267; 1:1,000) antibodies, were purchased from Cell Signaling Technology. Secondary Goat anti‐rabbit IgG starbright blue 700 and Anti‐tubulin hFAB‐Rhodamine were purchased from Bio‐Rad.

### 5.3 Crystallization and Structure Determination

EGFR(T790M/V948R) pre‐incubated with 1 mM AMP‐PNP and 10 mM MgCl_2_ on ice was prepared by hanging‐drop vapor diffusion over a reservoir solution containing 0.1 M Bis‐Tris (pH = 5.5), 25‐30% PEG‐3350, and 5 mM TCEP. Drops containing crystals in buffer were exchanged with solutions of buffers containing ~1.0 mM **6** three times for an hour and then left to soak overnight. Crystals were flash frozen after rapid immersion in a cryoprotectant solution with buffer containing 25% ethylene glycol. X‐ray diffraction data on soaked T790M/V948R crystals were collected at 100 K at the Advanced Light Source, a part of the Northeastern Collaborative

Access Team (NE‐CAT) on beamline 24‐ID‐C. Diffraction data were processed and merged in Xia2 using aimless and dials. The structure was determined by molecular replacement with the program PHASER using the inactive kinase domain EGFR(T790M/V948R) kinase from our previous work, excluding the LN3844 ligand (PDB 6WXN). Repeated rounds of manual refitting and crystallographic refinement were performed using COOT and Phenix. The inhibitor was modeled into the closely fitting positive *F*_o_ − *F*_c_ electron density and then included in the following refinement cycles. Statistics for diffraction data processing and structure refinement are shown in Supporting Information S2: Table S2. The co‐crystal structure of compound **6** in complex with EGFR has been validated and deposited on PDB (ID: 9N6G).

## Conflicts of Interest

Florian Wittlinger, Marcel Günther, Michael J. Eck, Stefan A. Laufer, and David E. Heppner are inventors of the patent US20230406838A1.

## Supporting information

ArchPharm SupplMat InChI.

SI SAR at back pocket submit R1 v02.

## Data Availability

The data that support the findings of this study are available on request from the corresponding author. The data are not publicly available due to privacy or ethical restrictions. The X‐ray cocrystal structure is available freely from the Protein Data Bank (PDB ID 9N6G).
