# Supplementary material for: Structure–Activity Relationships of Inactive‐Conformation Binding EGFR Inhibitors: Linking the ATP and Allosteric Pockets
Source: Arch Pharm (Weinheim). 2025 Jul 16;358(7):e70027. doi: 10.1002/ardp.70027 (PMC12264581; doi:10.1002/ardp.70027)
Supplement: Supplementary file 1 — ArchPharm SupplMat InChI. [file ARDP-358-e70027-s001.doc]

**Supplemental Material: Novel Compounds and Biological Screening Results**

**Structure-activity relationships of inactive-conformation binding EGFR inhibitors: Linking the ATP and allosteric pockets.**

Surbhi P. Chitnis,^1^ Florian Wittlinger,^2,3^ Mareike Möllers,^2^ Tyler J. Hartman,^1^ Marcel Günther,^2^ Michael J. Eck,^4,5^ Stefan A. Laufer,^2,6,7^* David E. Heppner^1,8,9,10^*

1. Department of Chemistry, University at Buffalo, The State University of New York, Buffalo, NY, 14260, USA

2. Department of Pharmaceutical and Medicinal Chemistry, Institute of Pharmaceutical Sciences, Eberhard Karls Universität Tübingen, Auf der Morgenstelle 8, 72076 Tübingen, Germany

3. Department of Medicinal Chemistry, Eberhard Karls Universität Tübingen, Faculty of Medicine, Institute for Biomedical Engineering, Tübingen, 72076, Germany

4. Department of Cancer Biology, Dana-Farber Cancer Institute, Boston, MA, 02215 USA

5. Department of Biological Chemistry and Molecular Pharmacology, Harvard Medical School, Boston, MA, 02115 USA

6. Cluster of Excellence iFIT (EXC 2180) “Image-Guided and Functionally Instructed Tumor Therapies” Eberhard Karls Universität Tübingen, 72076 Tübingen, Germany.

7. Tübingen Center for Academic Drug Discovery & Development (TüCAD2), 72076 Tübingen, Germany

8. Department of Structural Biology, University at Buffalo, The State University of New York, Buffalo, NY, 14260, USA

9. Department of Pharmaceutical Sciences, University at Buffalo, The State University of New York, Buffalo, NY 14214.

10. Department of Pharmacology and Therapeutics, Roswell Park Comprehensive Cancer Center, Buffalo, NY,14203, USA

*Corresponding authors

To whom correspondence may be addressed:

David E. Heppner [davidhep@buffalo.edu](mailto:davidhep@buffalo.edu)

Stefan A. Laufer [stefan.laufer@uni-tuebingen.de](mailto:stefan.laufer@uni-tuebingen.de)

| **Compound No.** | **InChI** | **Biological Activity (IC_50_)^a^** | |
| --- | --- | --- | --- |
|  |  | **LR** | **LR/TM** |
| 1 | InChI=1S/C17H17N5OS/c1-10(23)20-14-9-12(6-7-19-14)16-15(21-17(22-16)24-2)11-4-3-5-13(18)8-11/h3-9H,18H2,1-2H3,(H,21,22)(H,19,20,23) | > 10 μM | > 10^b^ μM |
| 2 | InChI=1S/C24H21N5O2S/c1-15(30)26-20-14-18(11-12-25-20)22-21(28-24(29-22)32-2)17-9-6-10-19(13-17)27-23(31)16-7-4-3-5-8-16/h3-14H,1-2H3,(H,27,31)(H,28,29)(H,25,26,30) | 0.045 ± 0.004^c^ μM | 0.59 ± 0.03^c^ μM |
| 3 | InChI=1S/C24H20FN5O2S/c1-14(31)27-20-13-17(10-11-26-20)22-21(29-24(30-22)33-2)16-4-3-5-19(12-16)28-23(32)15-6-8-18(25)9-7-15/h3-13H,1-2H3,(H,28,32)(H,29,30)(H,26,27,31) | 0.81 ± 0.1 μM | > 10 μM |
| 4 | InChI=1S/C24H20FN5O2S/c1-14(31)27-20-13-16(9-10-26-20)22-21(29-24(30-22)33-2)15-5-4-8-19(12-15)28-23(32)17-6-3-7-18(25)11-17/h3-13H,1-2H3,(H,28,32)(H,29,30)(H,26,27,31) | 0.10 ± 0.01 μM | 0.73 ± 0.08 μM |
| 5 | InChI=1S/C24H19F2N5O2S/c1-13(32)28-20-11-15(6-7-27-20)22-21(30-24(31-22)34-2)14-4-3-5-19(10-14)29-23(33)16-8-17(25)12-18(26)9-16/h3-12H,1-2H3,(H,29,33)(H,30,31)(H,27,28,32) | 0.47 ± 0.08 μM | > 10 μM |
| 6 | InChI=1S/C24H19F2N5O2S/c1-13(32)28-19-12-15(9-10-27-19)22-21(30-24(31-22)34-2)14-5-3-6-16(11-14)29-23(33)20-17(25)7-4-8-18(20)26/h3-12H,1-2H3,(H,29,33)(H,30,31)(H,27,28,32) | 0.015 ± 0.003 μM | 0.102 ± 0.007 μM |
| 7 | InChI=1S/C24H18F3N5O2S/c1-12(33)29-19-9-14(6-7-28-19)22-21(31-24(32-22)35-2)13-4-3-5-16(8-13)30-23(34)20-17(26)10-15(25)11-18(20)27/h3-11H,1-2H3,(H,30,34)(H,31,32)(H,28,29,33) | 0.31 ± 0.04 μM | 3.91 ± 1 μM |
| 8 | InChI=1S/C24H21N5O3S/c1-14(30)26-20-13-16(9-10-25-20)22-21(28-24(29-22)33-2)15-5-3-7-18(11-15)27-23(32)17-6-4-8-19(31)12-17/h3-13,31H,1-2H3,(H,27,32)(H,28,29)(H,25,26,30) | 0.0052 ± 0.0003^c^ μM | 0.051 ± 0.004^c^ μM |
| 9 | InChI=1S/C24H20FN5O3S/c1-13(31)27-20-11-15(8-9-26-20)22-21(29-24(30-22)34-2)14-4-3-5-16(10-14)28-23(33)18-12-17(32)6-7-19(18)25/h3-12,32H,1-2H3,(H,28,33)(H,29,30)(H,26,27,31) | 0.0055 ± 0.0003^d^ μM | 0.032 ± 0.003^d^ μM |
| 10 | InChI=1S/C23H20N6O2S/c1-14(30)26-19-12-16(8-10-25-19)21-20(28-23(29-21)32-2)15-5-3-7-18(11-15)27-22(31)17-6-4-9-24-13-17/h3-13H,1-2H3,(H,27,31)(H,28,29)(H,25,26,30) | 0.15 ± 0.01 μM | 1.83 ± 0.1 μM |
| 11 | InChI=1S/C23H20N6O2S/c1-14(30)26-19-13-16(9-11-25-19)21-20(28-23(29-21)32-2)15-6-5-7-17(12-15)27-22(31)18-8-3-4-10-24-18/h3-13H,1-2H3,(H,27,31)(H,28,29)(H,25,26,30) | 0.21 ± 0.01 μM | 2.11 ± 0.12 μM |
| 12 | InChI=1S/C23H20N6O2S/c1-14(30)26-19-13-17(8-11-25-19)21-20(28-23(29-21)32-2)16-4-3-5-18(12-16)27-22(31)15-6-9-24-10-7-15/h3-13H,1-2H3,(H,27,31)(H,28,29)(H,25,26,30) | 2.5 ± 0.3 μM | > 10 μM |
| 13 | InChI=1S/C27H24N6O2S/c1-16(34)29-23-15-18(10-12-28-23)25-24(31-27(32-25)36-3)17-6-4-7-19(14-17)30-26(35)21-8-5-9-22-20(21)11-13-33(22)2/h4-15H,1-3H3,(H,30,35)(H,31,32)(H,28,29,34) | 0.323 ± 0.03 μM | 1.4 ± 0.2 μM |
| 14 | InChI=1S/C28H23N5O2S/c1-17(34)30-24-16-20(13-14-29-24)26-25(32-28(33-26)36-2)19-9-5-10-21(15-19)31-27(35)23-12-6-8-18-7-3-4-11-22(18)23/h3-16H,1-2H3,(H,31,35)(H,32,33)(H,29,30,34) | 0.062 ± 0.005 μM | 0.22 ± 0.02 μM |
| 15 | InChI=1S/C28H23N5O2S/c1-17(34)30-24-16-21(12-13-29-24)26-25(32-28(33-26)36-2)20-8-5-9-23(15-20)31-27(35)22-11-10-18-6-3-4-7-19(18)14-22/h3-16H,1-2H3,(H,31,35)(H,32,33)(H,29,30,34) | > 10 μM | > 10 μM |
| 16 | InChI=1S/C22H19N5O2S2/c1-13(28)24-18-11-15(6-8-23-18)20-19(26-22(27-20)30-2)14-4-3-5-17(10-14)25-21(29)16-7-9-31-12-16/h3-12H,1-2H3,(H,25,29)(H,26,27)(H,23,24,28) | 0.12 ± 0.007 μM | 2.4 ± 0.2 μM |
| 17 | InChI=1S/C22H19N5O2S2/c1-13(28)24-18-12-15(8-9-23-18)20-19(26-22(27-20)30-2)14-5-3-6-16(11-14)25-21(29)17-7-4-10-31-17/h3-12H,1-2H3,(H,25,29)(H,26,27)(H,23,24,28) | 0.093 ± 0.02 μM | 1.5 ± 0.1 μM |
| 18 | InChI=1S/C18H19N5O3S2/c1-11(24)20-15-10-13(7-8-19-15)17-16(21-18(22-17)27-2)12-5-4-6-14(9-12)23-28(3,25)26/h4-10,23H,1-3H3,(H,21,22)(H,19,20,24) | 0.43 ± 0.06 μM | 7.71 ± 0.70 μM |
| 19 | InChI=1S/C20H23N5O3S2/c1-4-10-30(27,28)25-16-7-5-6-14(11-16)18-19(24-20(23-18)29-3)15-8-9-21-17(12-15)22-13(2)26/h5-9,11-12,25H,4,10H2,1-3H3,(H,23,24)(H,21,22,26) | 0.66 ± 0.03 μM | 1.75 ± 0.11 μM |
| 20 | InChI=1S/C20H21N5O3S2/c1-12(26)22-17-11-14(8-9-21-17)19-18(23-20(24-19)29-2)13-4-3-5-15(10-13)25-30(27,28)16-6-7-16/h3-5,8-11,16,25H,6-7H2,1-2H3,(H,23,24)(H,21,22,26) | 0.13 ± 0.01 μM | 5.10 ± 0.33 μM |
| 21 | InChI=1S/C23H21N5O3S2/c1-15(29)25-20-14-17(11-12-24-20)22-21(26-23(27-22)32-2)16-7-6-8-18(13-16)28-33(30,31)19-9-4-3-5-10-19/h3-14,28H,1-2H3,(H,26,27)(H,24,25,29) | 1.23 ± 0.08 μM | 1.51 ± 0.19 μM |
| 22 | InChI=1S/C24H20FN5O2S/c1-14(31)27-20-13-17(10-11-26-20)22-21(29-24(30-22)33-2)16-8-9-18(25)19(12-16)28-23(32)15-6-4-3-5-7-15/h3-13H,1-2H3,(H,28,32)(H,29,30)(H,26,27,31) | 0.042 ± 0.004 μM | 1.23 ± 0.15 μM |
| 23 | InChI=1S/C24H18F3N5O2S/c1-12(33)29-19-11-14(8-9-28-19)22-21(31-24(32-22)35-2)13-6-7-15(25)18(10-13)30-23(34)20-16(26)4-3-5-17(20)27/h3-11H,1-2H3,(H,30,34)(H,31,32)(H,28,29,33) | 0.062 ± 0.008 μM | 1.21 ± 0.24 μM |
| 24 | InChI=1S/C24H18F3N5O2S/c1-12(33)29-20-10-14(5-6-28-20)22-21(31-24(32-22)35-2)13-3-4-18(27)19(9-13)30-23(34)15-7-16(25)11-17(26)8-15/h3-11H,1-2H3,(H,30,34)(H,31,32)(H,28,29,33) | 0.517 ± 0.071 μM | > 10 μM |
| 25 | InChI=1S/C24H21N5O2S/c1-15(30)26-20-14-17(12-13-25-20)21-22(29-24(28-21)32-2)18-10-6-7-11-19(18)27-23(31)16-8-4-3-5-9-16/h3-14H,1-2H3,(H,27,31)(H,28,29)(H,25,26,30) | > 10 μM | > 10 μM |
| 26 | InChI=1S/C24H20FN5O2S/c1-14(31)27-20-13-16(11-12-26-20)21-22(30-24(29-21)33-2)18-5-3-4-6-19(18)28-23(32)15-7-9-17(25)10-8-15/h3-13H,1-2H3,(H,28,32)(H,29,30)(H,26,27,31) | > 10 μM | > 10 μM |
| 27 | InChI=1S/C24H19F2N5O2S/c1-13(32)28-19-12-14(10-11-27-19)21-22(31-24(30-21)34-2)15-6-3-4-9-18(15)29-23(33)20-16(25)7-5-8-17(20)26/h3-12H,1-2H3,(H,29,33)(H,30,31)(H,27,28,32) | > 10 μM | > 10 μM |
| 28 | InChI=1S/C24H21N5O3S/c1-14(30)26-20-13-15(10-11-25-20)21-22(29-24(28-21)33-2)18-8-3-4-9-19(18)27-23(32)16-6-5-7-17(31)12-16/h3-13,31H,1-2H3,(H,27,32)(H,28,29)(H,25,26,30) | > 10 μM | > 10 μM |
| 29 | InChI=1S/C23H20N6O2S/c1-14(30)26-19-12-15(9-11-25-19)20-21(29-23(28-20)32-2)17-7-3-4-8-18(17)27-22(31)16-6-5-10-24-13-16/h3-13H,1-2H3,(H,27,31)(H,28,29)(H,25,26,30) | > 10 μM | > 10 μM |
| 30 | InChI=1S/C23H20N6O2S/c1-14(30)26-19-13-15(10-12-25-19)20-21(29-23(28-20)32-2)16-7-3-4-8-17(16)27-22(31)18-9-5-6-11-24-18/h3-13H,1-2H3,(H,27,31)(H,28,29)(H,25,26,30) | > 10 μM | > 10 μM |
| 31 | InChI=1S/C23H20N6O2S/c1-14(30)26-19-13-16(9-12-25-19)20-21(29-23(28-20)32-2)17-5-3-4-6-18(17)27-22(31)15-7-10-24-11-8-15/h3-13H,1-2H3,(H,27,31)(H,28,29)(H,25,26,30) | > 10 μM | > 10 μM |

^a^IC_50_ values were measured from non-linear least squares fitting of HTRF activity data collected in triplicate. ATP concentration of 100 μM. Errors are reported as ± the standard error. ^b^Data from Wittlinger and Ogboo et al. ^c^Data from Wittlinger et al. ^d^Data from Wittlinger and Heppner et al.
