# Supplementary material for: Structure–Activity Relationships of Inactive‐Conformation Binding EGFR Inhibitors: Linking the ATP and Allosteric Pockets
Source: Arch Pharm (Weinheim). 2025 Jul 16;358(7):e70027. doi: 10.1002/ardp.70027 (PMC12264581; doi:10.1002/ardp.70027)

Supporting Information for

**Structure-activity relationships of inactive-conformation binding EGFR inhibitors: Linking the ATP and allosteric pockets.**

Surbhi P. Chitnis,^1^ Florian Wittlinger,^2,3^ Mareike Möllers,^2^ Tyler J. Hartman,^1^ Marcel Günther,^2^ Michael J. Eck,^4,5^ Stefan A. Laufer,^2,6,7^* David E. Heppner^1,8,9,10^*

1. Department of Chemistry, University at Buffalo, The State University of New York, Buffalo, NY, 14260, USA

2. Department of Pharmaceutical and Medicinal Chemistry, Institute of Pharmaceutical Sciences, Eberhard Karls Universität Tübingen, Auf der Morgenstelle 8, 72076 Tübingen, Germany

3. Department of Medicinal Chemistry, Eberhard Karls Universität Tübingen, Faculty of Medicine, Institute for Biomedical Engineering, Tübingen, 72076, Germany

4. Department of Cancer Biology, Dana-Farber Cancer Institute, Boston, MA, 02215 USA

5. Department of Biological Chemistry and Molecular Pharmacology, Harvard Medical School, Boston, MA, 02115 USA

6. Cluster of Excellence iFIT (EXC 2180) “Image-Guided and Functionally Instructed Tumor Therapies” Eberhard Karls Universität Tübingen, 72076 Tübingen, Germany.

7. Tübingen Center for Academic Drug Discovery & Development (TüCAD2), 72076 Tübingen, Germany

8. Department of Structural Biology, University at Buffalo, The State University of New York, Buffalo, NY, 14260, USA

9. Department of Pharmaceutical Sciences, University at Buffalo, The State University of New York, Buffalo, NY 14214.

10. Department of Pharmacology and Therapeutics, Roswell Park Comprehensive Cancer Center, Buffalo, NY,14203, USA

*Corresponding authors

To whom correspondence may be addressed:

David E. Heppner [davidhep@buffalo.edu](mailto:davidhep@buffalo.edu)

Stefan A. Laufer [stefan.laufer@uni-tuebingen.de](mailto:stefan.laufer@uni-tuebingen.de)

Table of Contents

1. Topological arrangement of sites of the EGFR kinase domain.........................................................................S3
2. Synthesis of novel 3-amino-4-fluorophenyl derivatives....................................................................................S4
3. Synthesis of the imidazole-scaffold with *ortho*-substituted aniline linker..........................................................S5
4. Biochemical Activities of Inhibitors with *ortho*-substituted anilino-linkers ..……………………...................S6
5. Immunoblotting of phosphorylation levels of EGFR in HCC827 (ex19del) cells ……………………………S7
6. Crystallographic data and refinement statistics ……….………….................………………………………..S7
7. Biochemical Activities against Mutant EGFR of Type I½ Inhibitors vs AABIs .............................................S9
8. NMR Spectra ……………………………………………………………………………………..…………S10


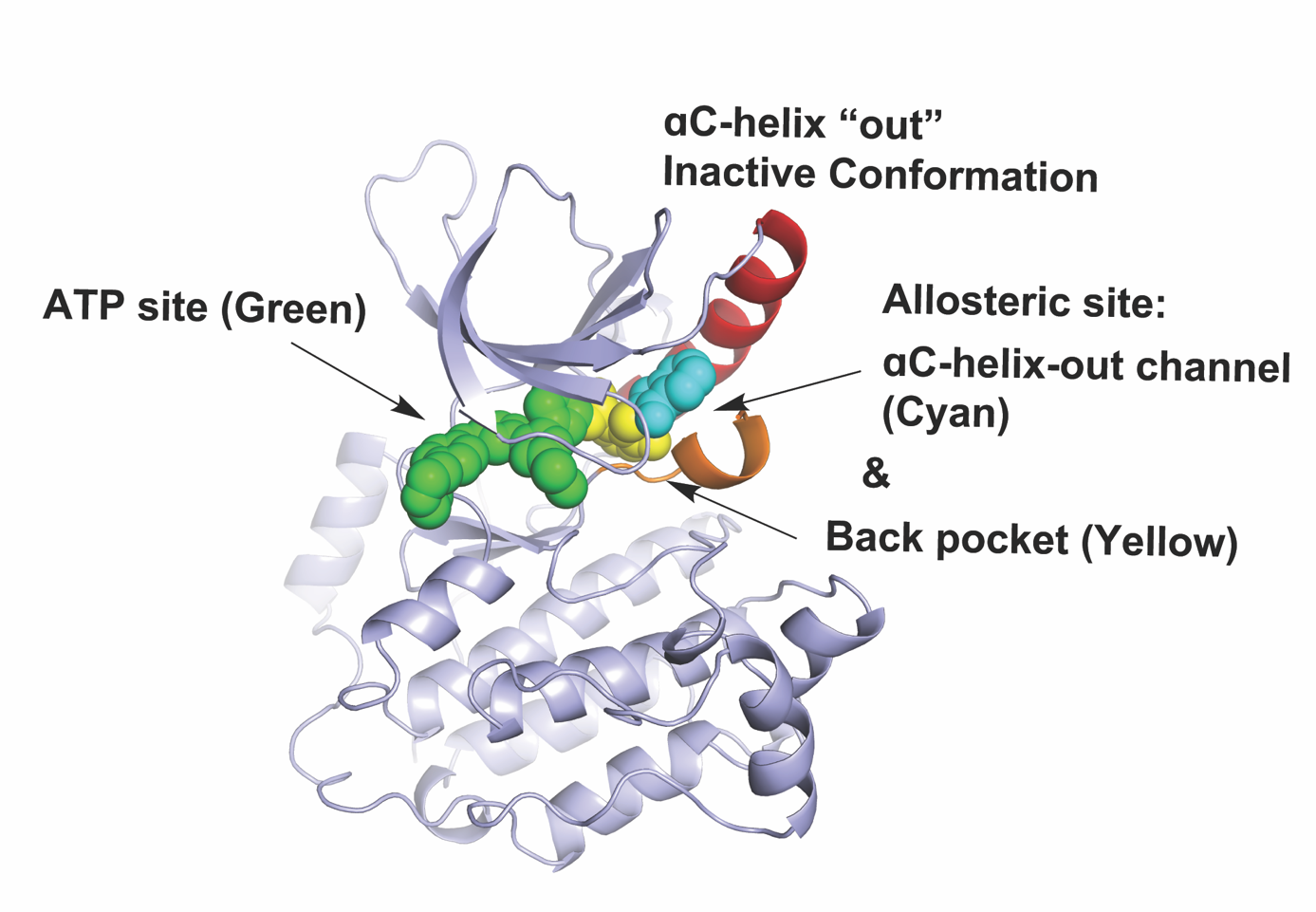


**Figure S1:** Topological arrangement of sites of the EGFR kinase domain as spheres: ATP site (green), Back pocket (yellow), and ⍺C-helix-out channel (cyan) of allosteric site (PDB ID 8TO3).

**Scheme S1:** Synthesis of novel 3-amino-4-fluorophenyl derivatives: i) Bis(pinacolato)diboron, KOAc, 1,4-dioxane, Pd(dppf)Cl_2_, 90 °C, quant.; ii) 4-Bromo-2-(methylthio)-1-((2-(trimethylsilyl)ethoxy)methyl)-1*H*-imidazole, K_3_PO_4_, 1,4-dioxane/H_2_O, P(*t*-Bu)_3_ Pd G3, 50°C, 83%; iii) NBS, ACN, -30°C, 87%; iv) *N*-(4-(4,4,5,5-tetramethyl-1,3,2-dioxaborolan-2-yl)pyridin-2-yl)acetamide, K_3_PO_4_, 1,4-dioxane/H_2_O, P(*t*-Bu)_3_ Pd G3, 50°C, 74%; v) Zn, NH_4_Cl, MeOH, rt, quant.; vi) amide coupling with corresponding benzoic acid according to general method A; vii) SEM deprotection according to general method B.

Scheme S2: Synthesis of the imidazole-scaffold with *ortho*-substituted aniline linker: i) Br_2_, CHCl_3_, rt, quant; ii) Hexamethylenetetramine, CHCl_3_, 70°C, then EtOH, conc. HCl (aq.), rt, 70%; iii) KSCN, AcOH, rf, 29%, iv) K_2_CO_3_, MeOH, methyl iodide, rt, 58%, v) NaH, SEM-Cl, THF, then cat. SEM-Cl, ACN, 80°C, 76%, vi) NBS, ACN, -30 °C, 89%; vii) *N*-(4-(4,4,5,5-tetramethyl-1,3,2-dioxaborolan-2-yl)pyridin-2-yl)acetamide, K_3_PO_4_, P(*t*-Bu)_3_Pd G3, 1,4-dioxane/H_2_O, 50°C, 65%;viii) Zn, NH_4_HCO_2_, MeOH, rt, 94%; ix) amide coupling with corresponding benzoic acid according to general method A; x) SEM deprotection according to general method B.

Table S1*:* Biochemical Activities against Mutant EGFR of Inhibitors with *ortho*-substituted anilino-linker derivatives.

|  | | | |
| --- | --- | --- | --- |
| **Compound** | **R** | **EGFR IC_50_ [μM] ^[a]^** | |
|  |  | **LR** | **LR/TM** |
| 25 |  | > 10 | > 10 |
| 26 |  | > 10 | > 10 |
| 27 |  | > 10 | > 10 |
| 28 |  | > 10 | > 10 |
| 29 |  | > 10 | > 10 |
| 30 |  | > 10 | > 10 |
| 31 |  | > 10 | > 10 |

[a] IC_50_ values were measured from non-linear least squares fitting of HTRF activity data collected in triplicate. ATP concentration of 100 μM.


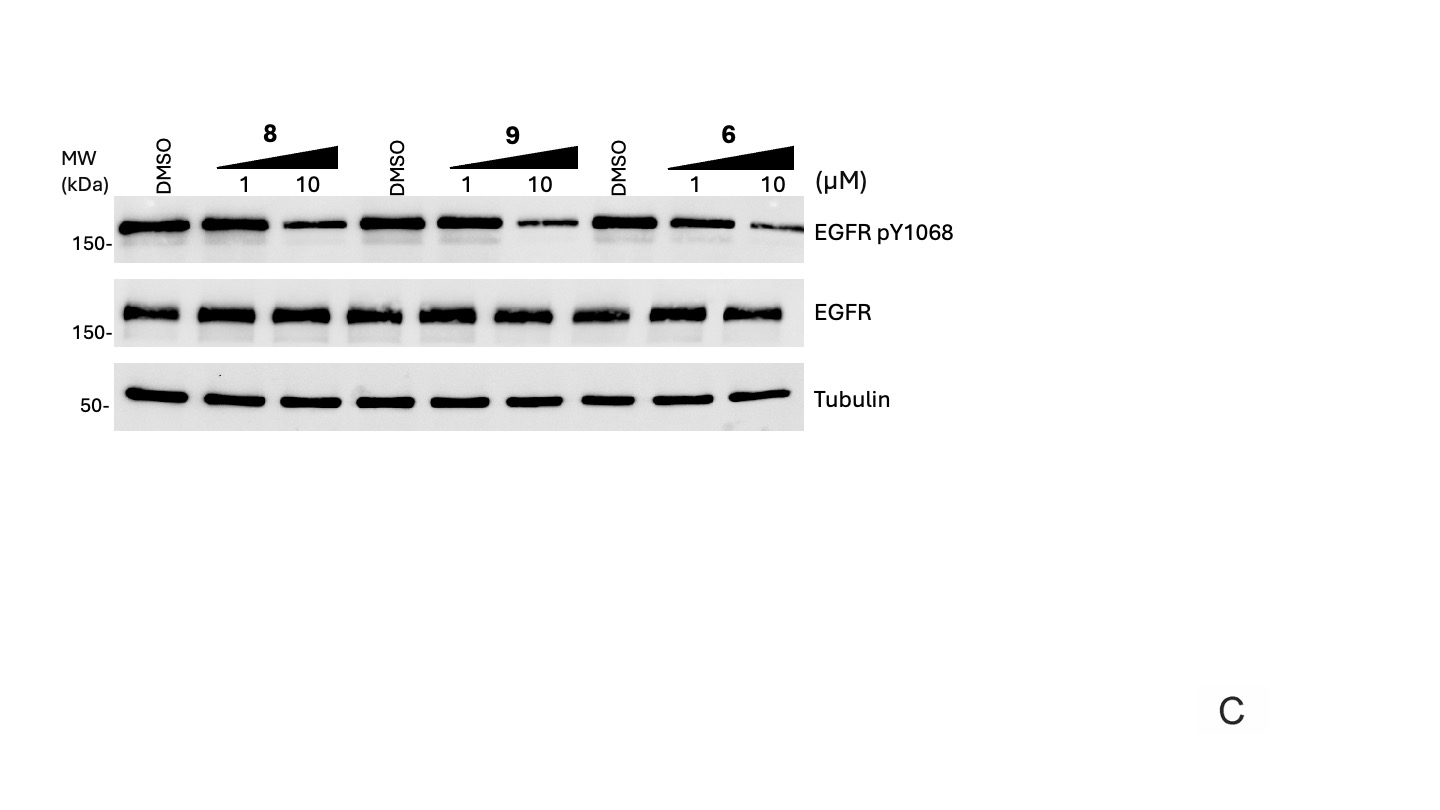


**Figure S2:** Immunoblotting of phosphorylation levels of EGFR in HCC827 (ex19del) cells. All experiments performed after 6 hours treatments and Western blots are representative of three independent experiments.

**Table S2** Crystallographic data collection and refinement statistics for compound **6** in complex with EGFR(T790M/V948R) crystals. Values in parenthesis pertain to the highest resolution shell.

| PDB ID  Compound | 9N6G  **6 (LN2827)** |
| --- | --- |
| Wavelength | 0.97920 |
| Resolution range | 85.63 – 2.59 (2.65 – 2.59) |
| Space group | P 1 21 1 |
| Unit cell  a, b, c (Å)  ɑ, β, γ (°) | 71.47, 101.88, 87.73  90, 102.55, 90 |
| Total reflections | 249988 (17709) |
| Unique reflections | 37605 (4243) |
| Multiplicity | 6.7 (6.7) |
| Completeness (%) | 98.14 (89.14) |
| Mean I/sigma(I) | 9.96 (0.98) |
| Wilson B-factor | 45.94 |
| R-merge | 0.135 (0.5541) |
| R-meas | 0.1464 (0.6009) |
| R-pim | 0.05606 (0.2297) |
| CC1/2 | 0.994 (0.904) |
| CC* | 0.999 (0.974) |
| Reflections used in refinement | 37570 (2430) |
| Reflections used for R-free | 2000 (130) |
| R-work | 0.210 (0.2172) |
| R-free | 0.255 (0.2781) |
| Number of non-hydrogen atoms | 8948 |
| macromolecules | 8797 |
| ligands | 136 |
| solvent | 15 |
| Protein residues | 1124 |
| RMS(bonds) | 0.009 |
| RMS (angles) | 1.02 |
| Ramachandran favored (%) | 97.42 |
| Ramachandran allowed (%) | 2.58 |
| Ramachandran outliers (%) | 0.00 |
| Rotamer outliers (%) | 5.55 |
| Clashscore | 2.34 |
| Average B-factor | 46.35 |
| macromolecules | 46.45 |
| ligands | 40.07 |
| solvent | 40.45 |

Table S3*:* Biochemical Activities against Mutant EGFR of Type I½ Inhibitors vs AABIs.

| **Compound** | **LR IC_50_ (nM)** | **LR/TM IC_50_ (nM)** |
| --- | --- | --- |
| 2c | 1.2 ± 0.4 ^[a]^ | 51 ± 3 ^[a]^ |
| 2 | 1.5 ± 0.3 ^[b]^ | 0.059 ± 0.005 ^[b]^ |
| 20 | 2 ± 0.2 ^[c]^ | 0.91 ± 0.1^[c]^ |
| 6 (This work) | 15 ± 3 | 102 ± 7 |
| 8 (This work) | 5.2 ± 0.3 ^[c]^ | 51 ± 4 ^[c]^ |
| 9 (This work) | 5.5 ± 0.3 ^[a]^ | 32 ± 3 ^[a]^ |
| Lapatinib | < 0.1^[c]^ | > 1000 ^[c]^ |

[a] Data from Wittlinger and Heppner et al.[^38^](#_ENREF_38) [b] Data from Wittlinger and Ogboo et al.[^39^](#_ENREF_39) [c] Data from Wittlinger et al.[^6^](#_ENREF_6)

**NMR Spectra**

**S2**


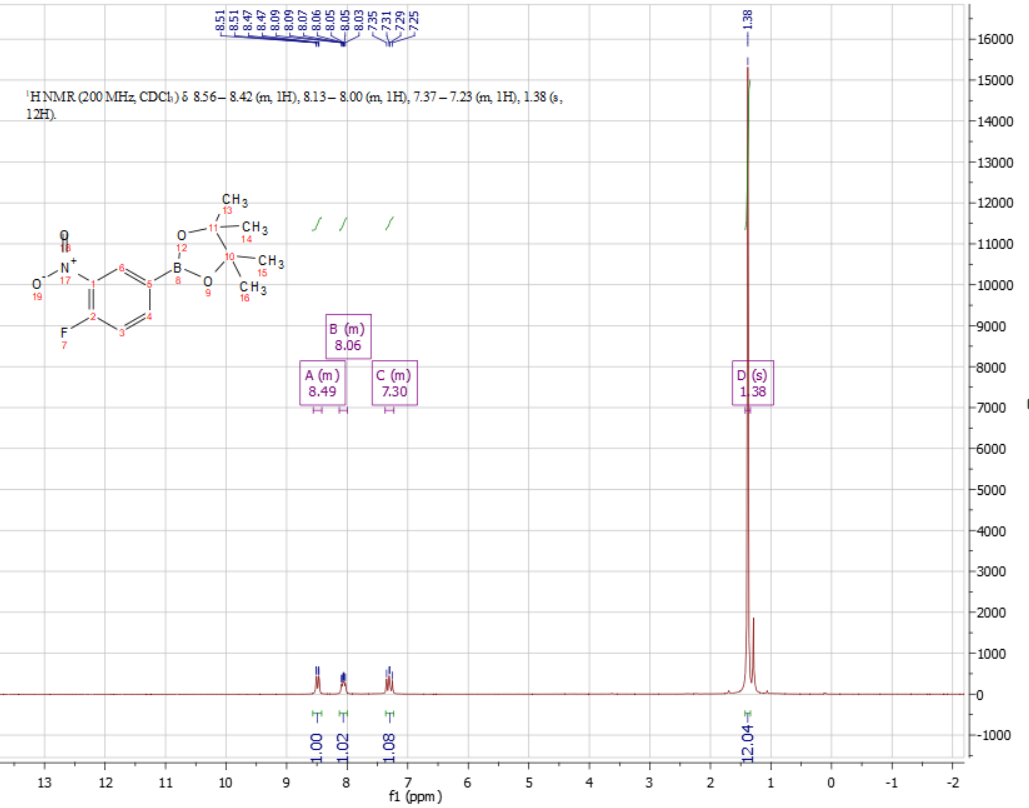


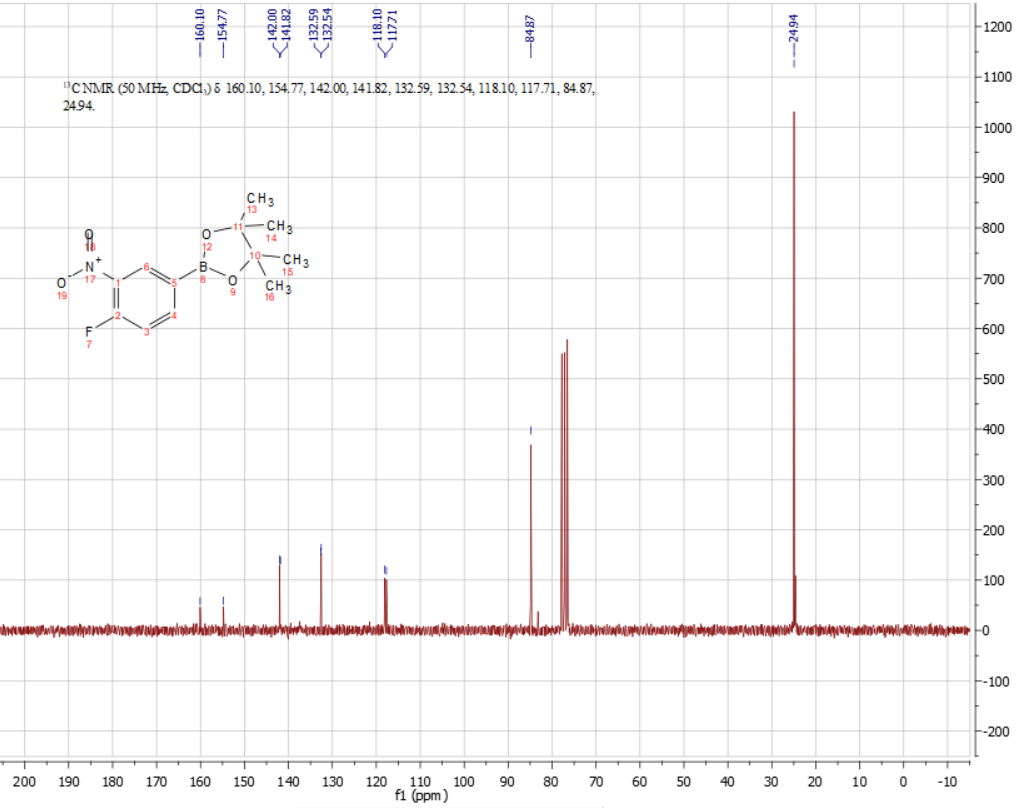


**S3**


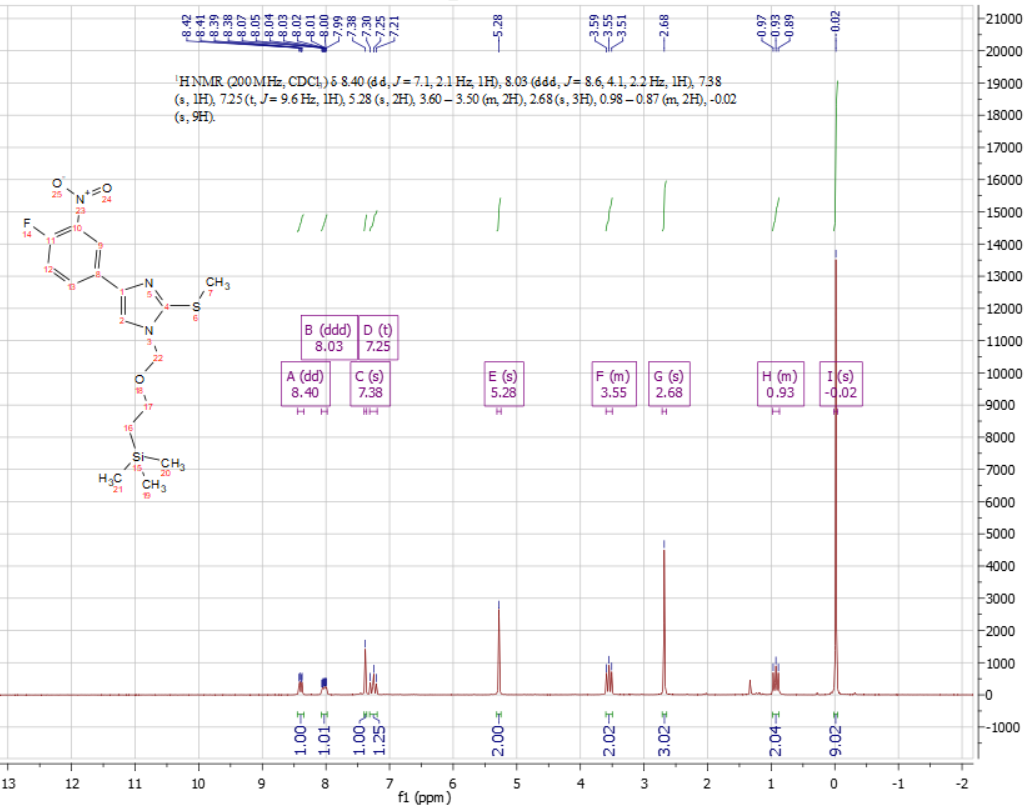


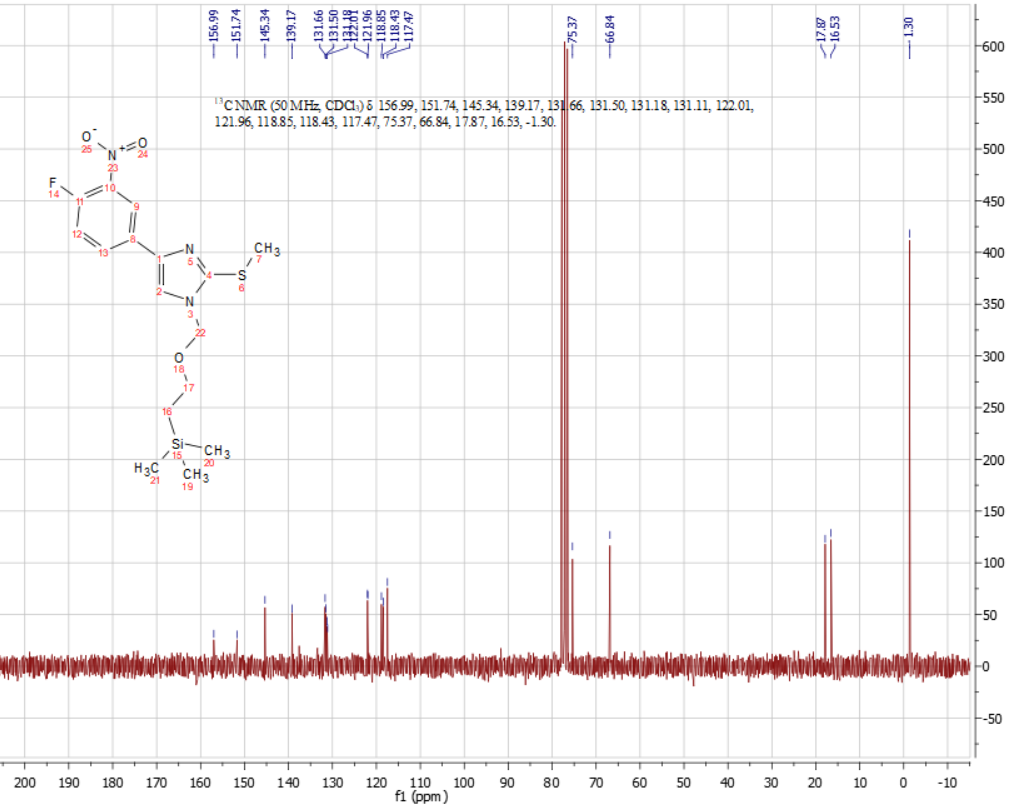


**S4**


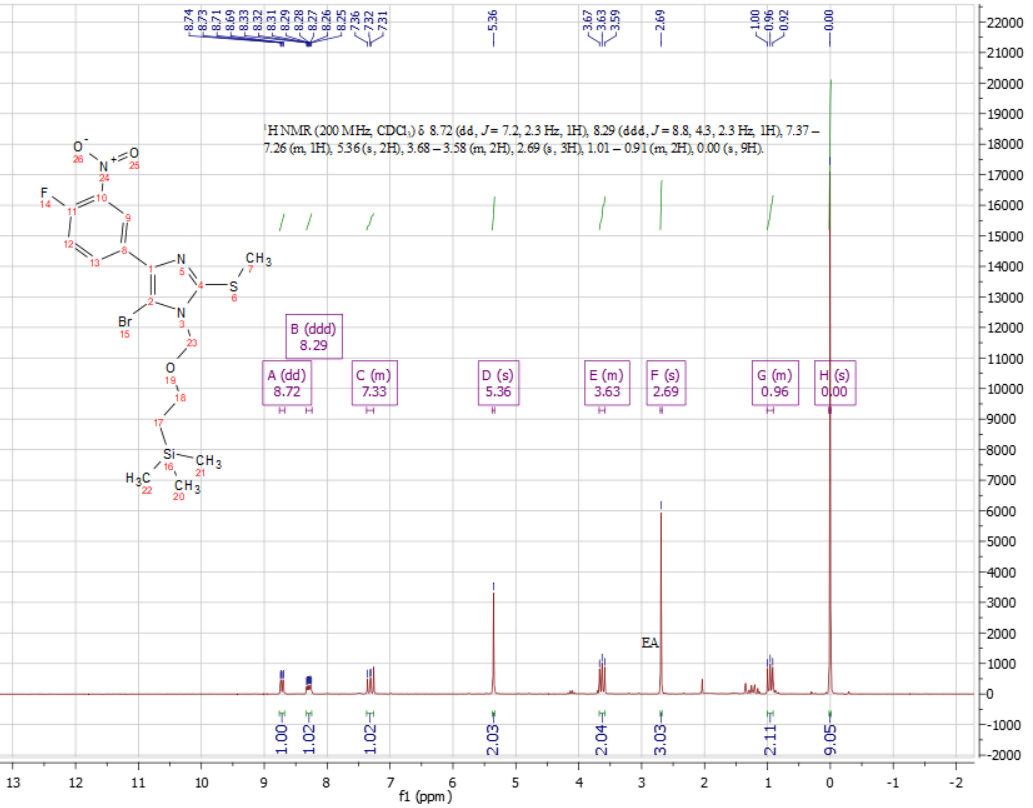


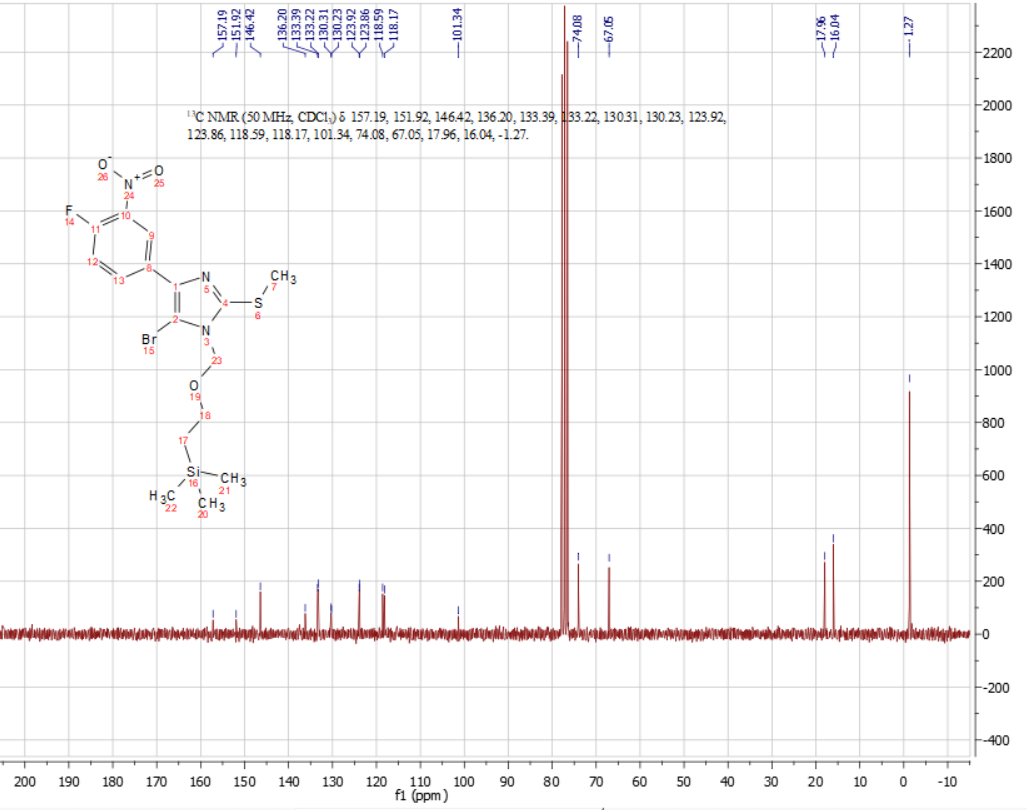


**S5**


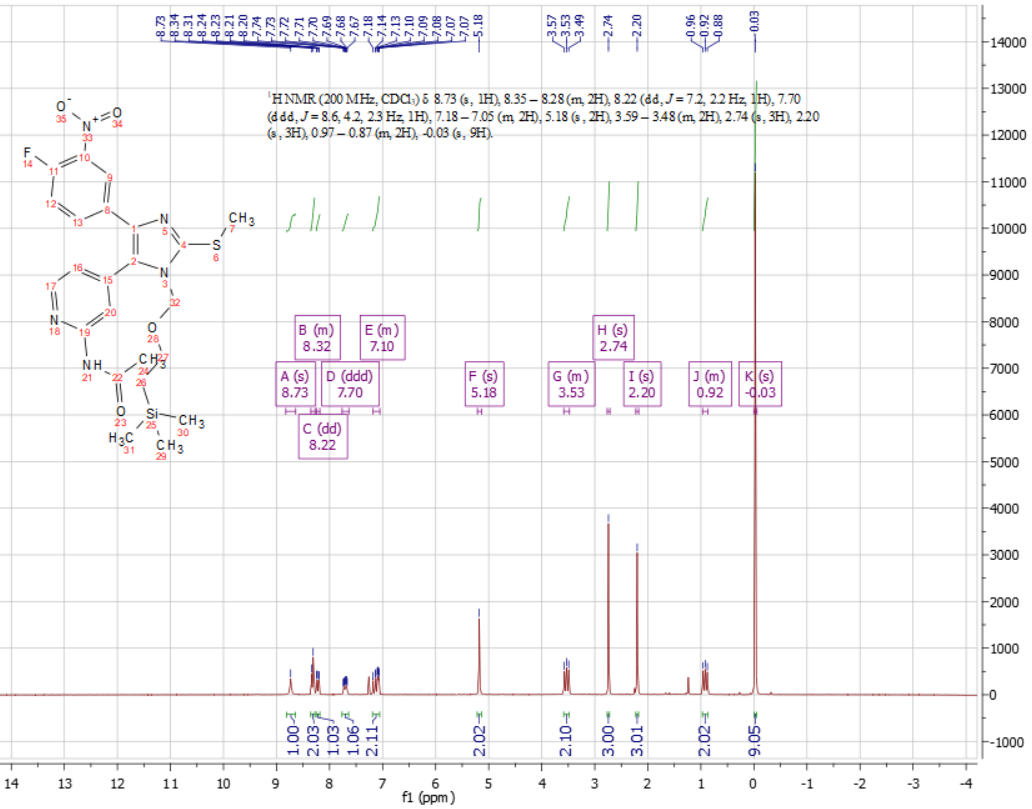


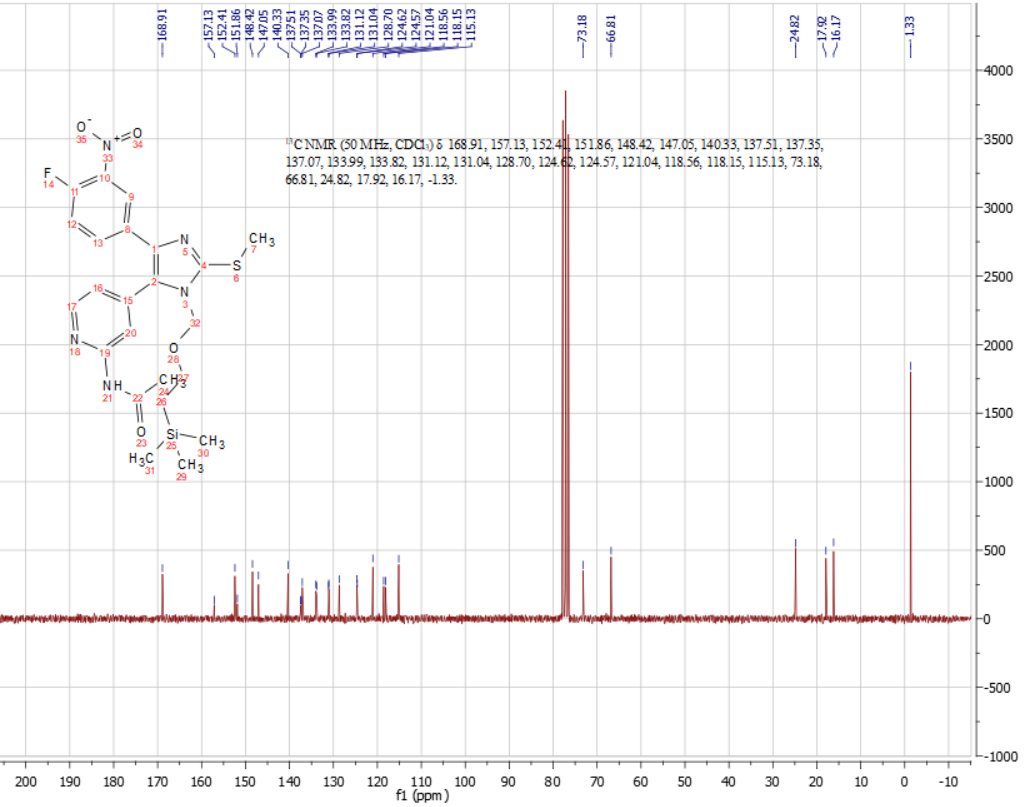


**S6**


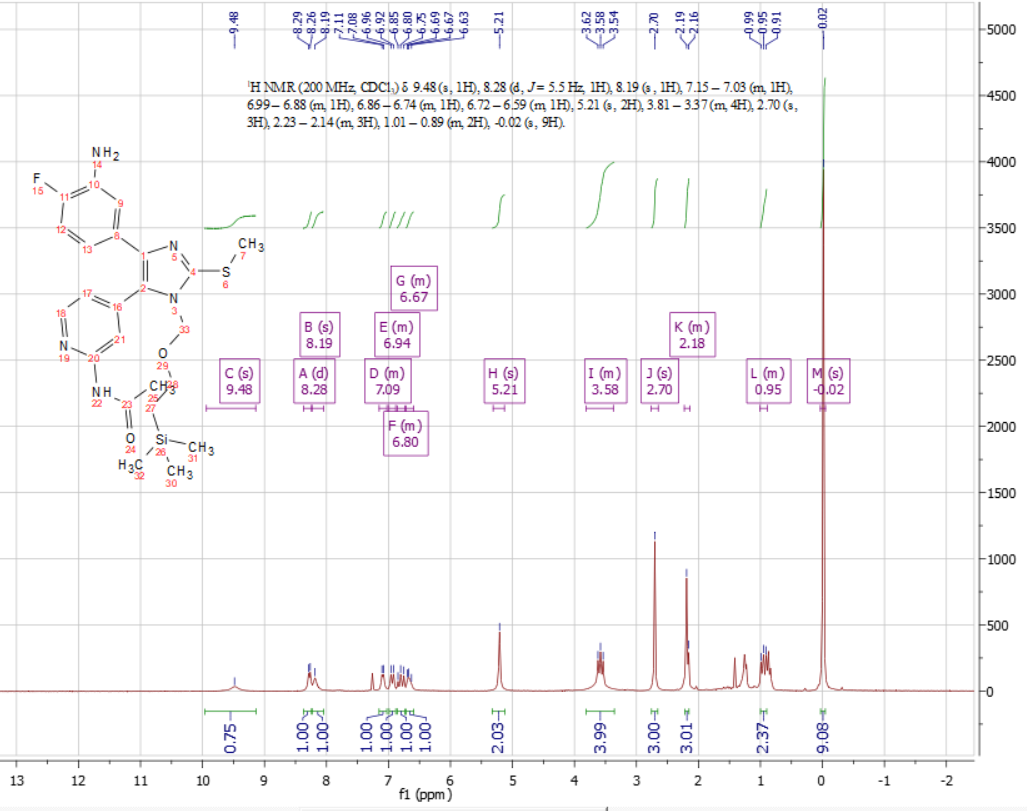


**S7**


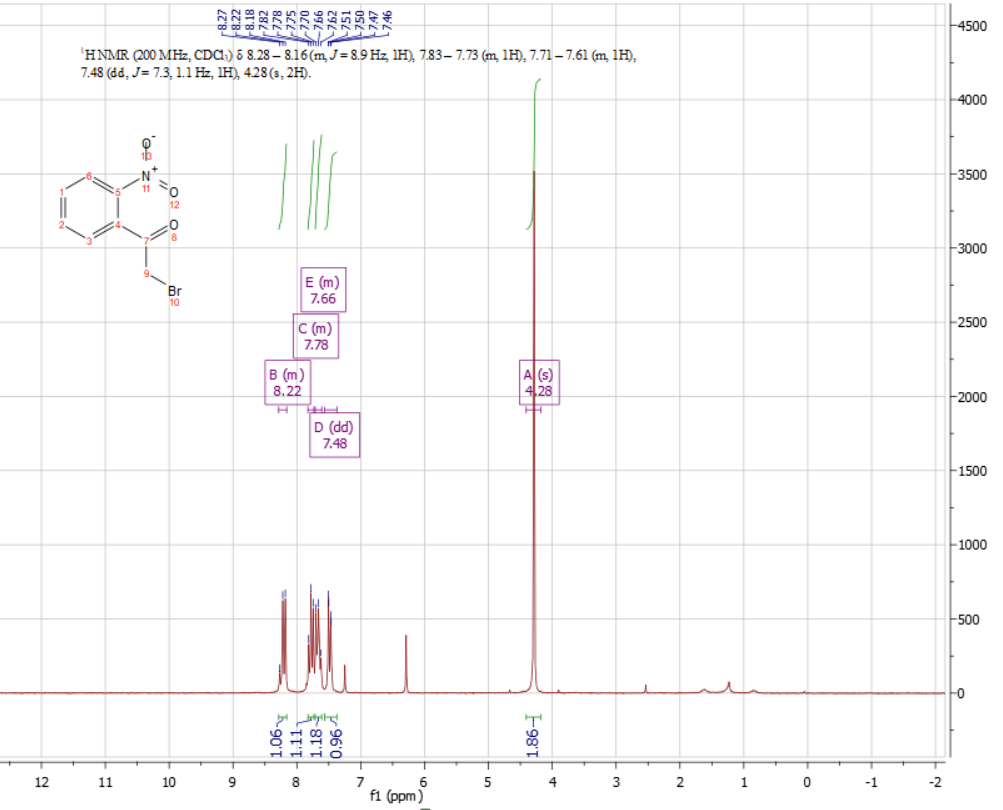

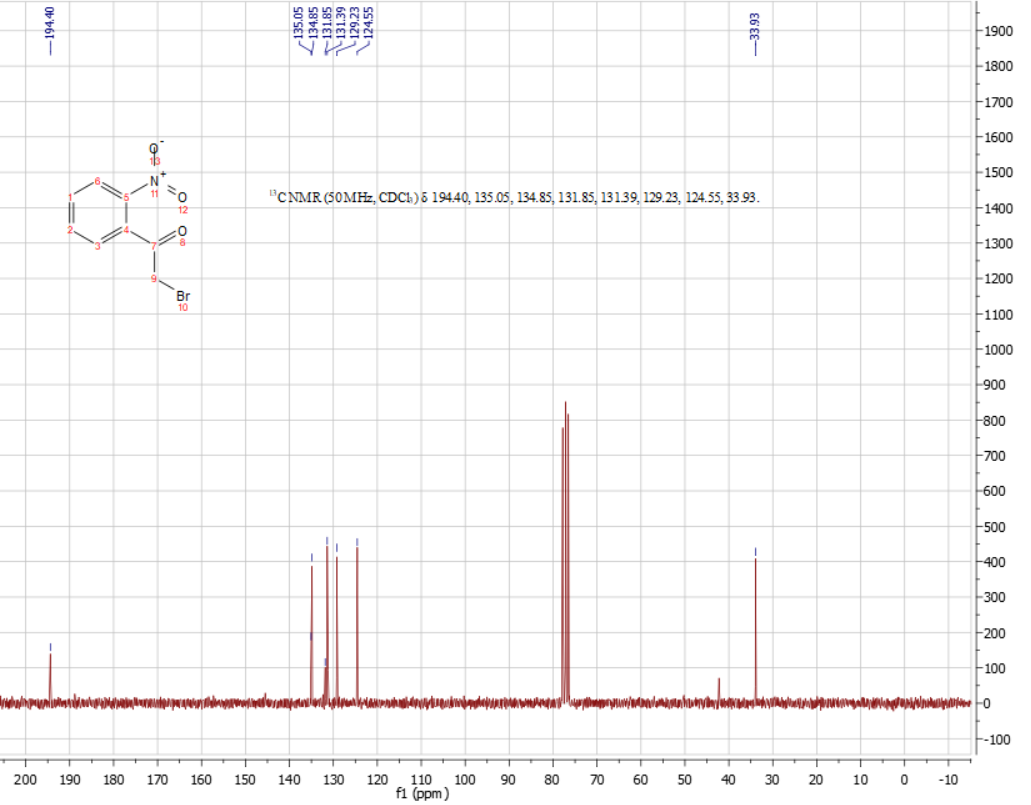


**S8**


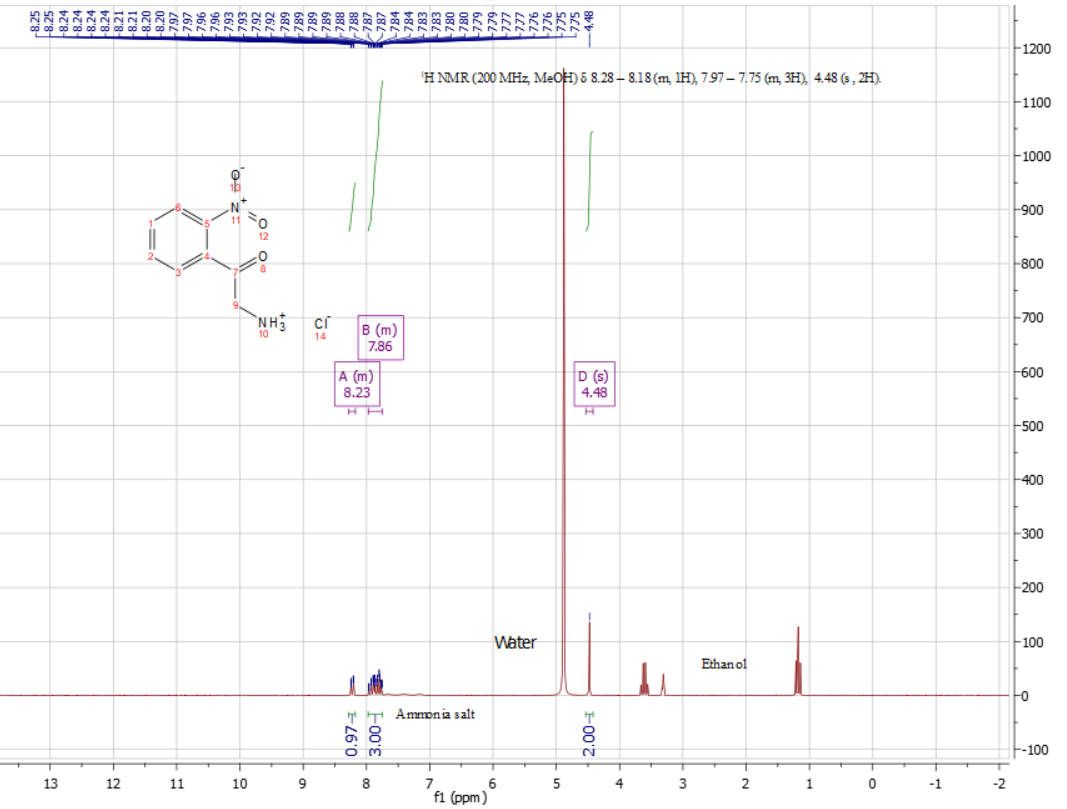

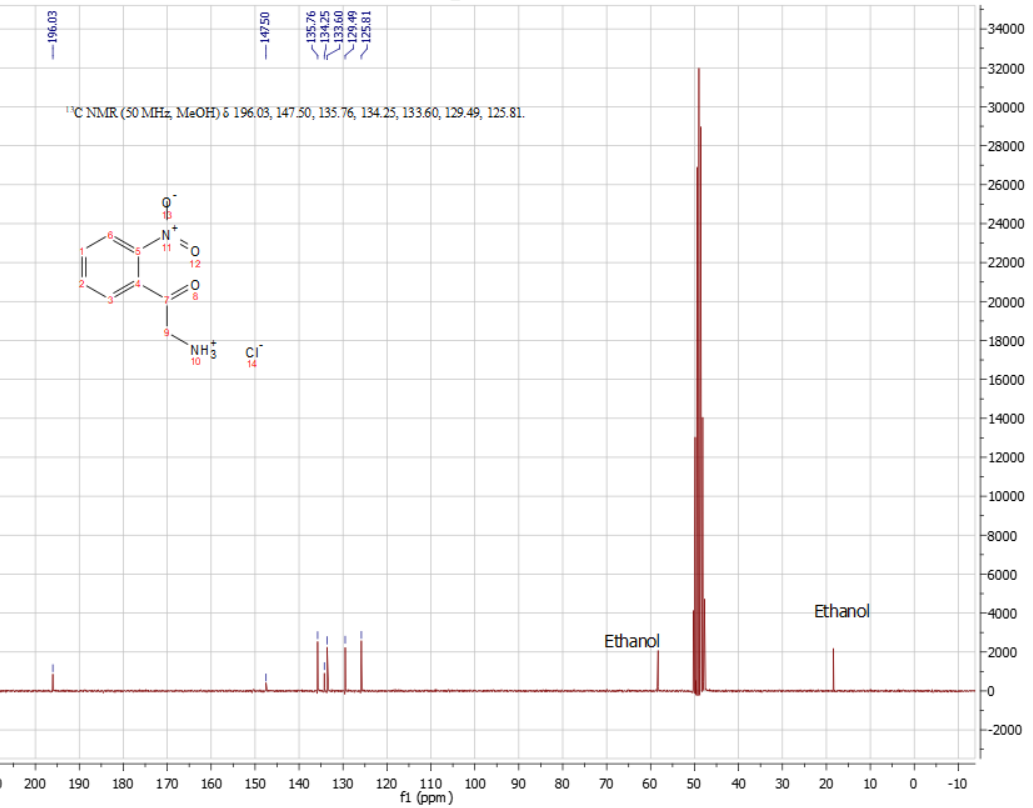


**S9**


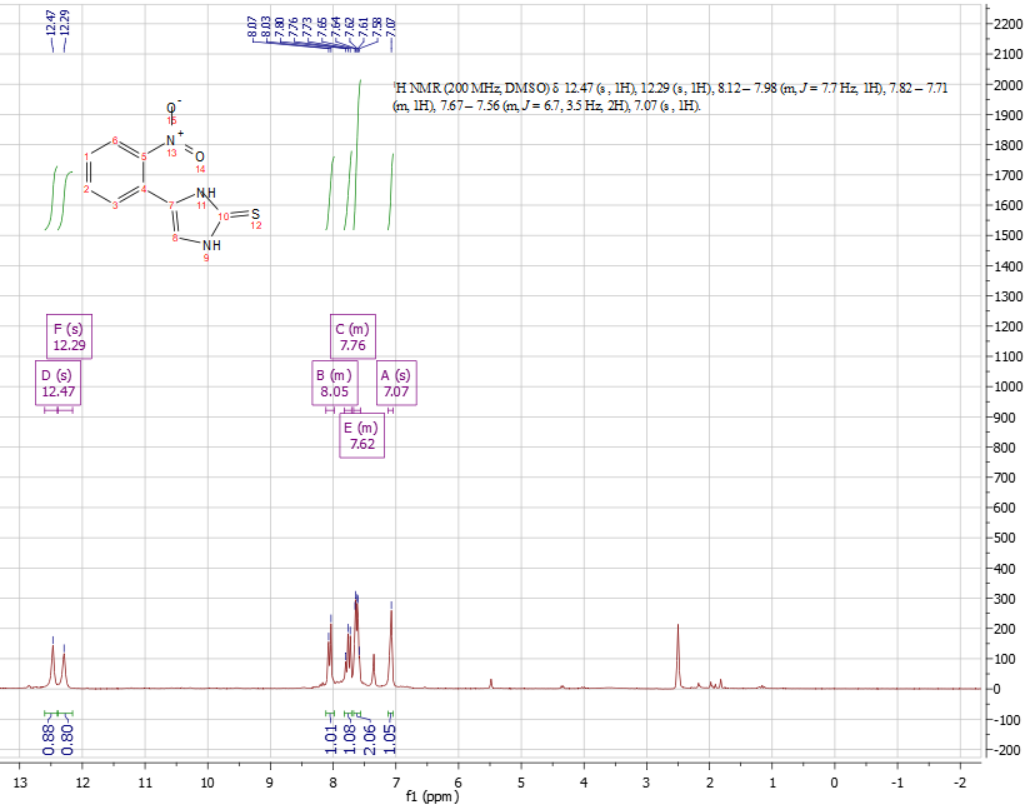


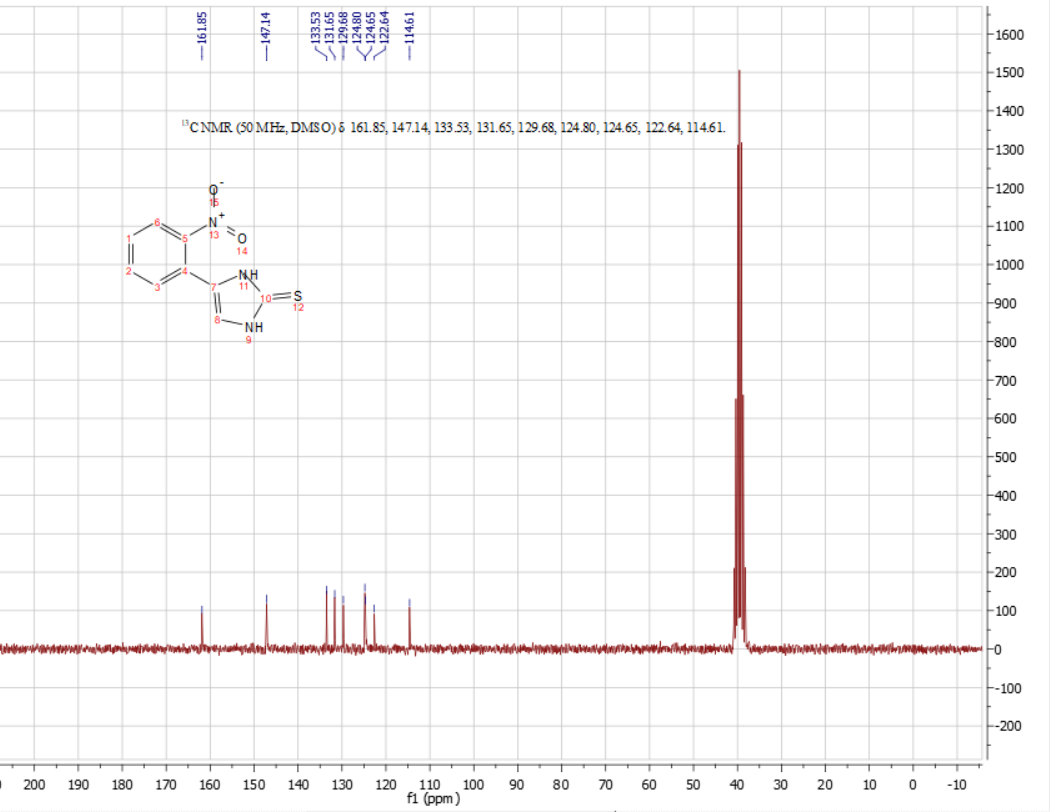


**S10**


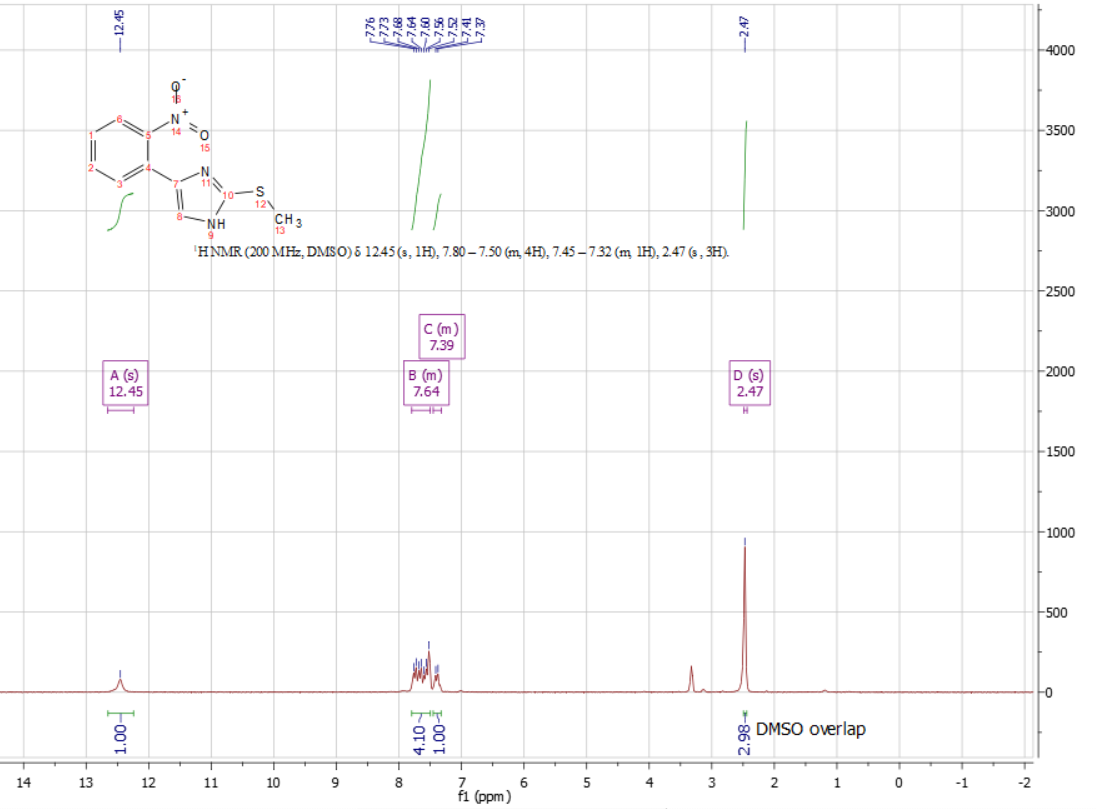


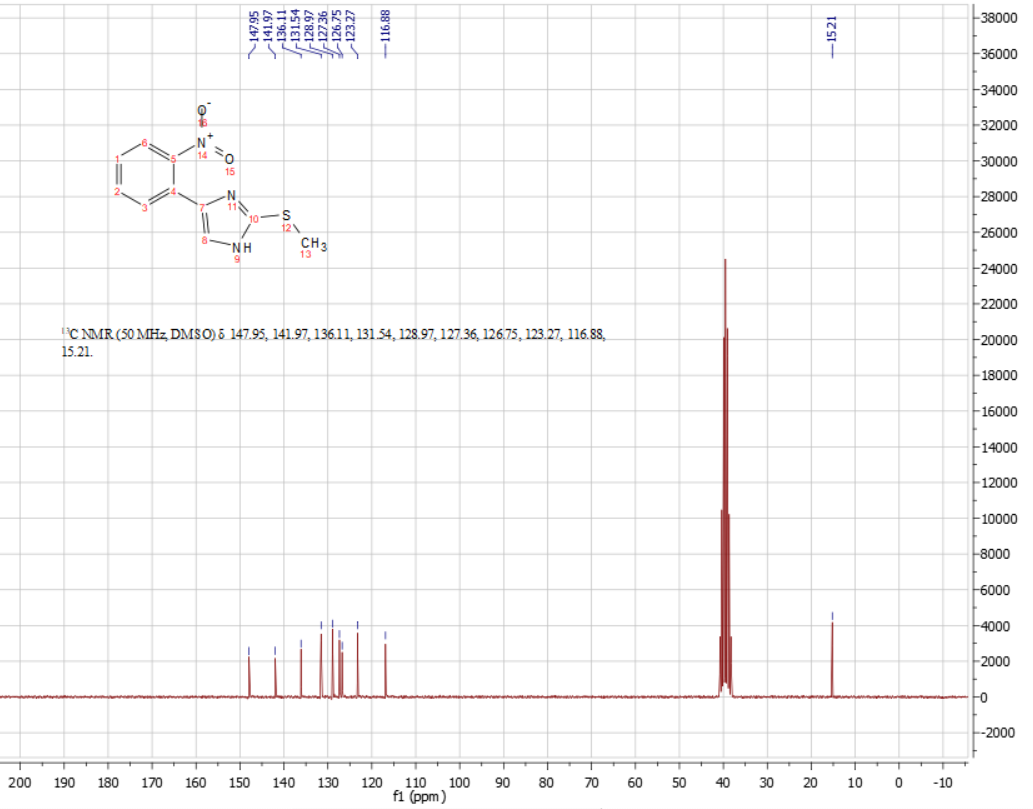


**S11**


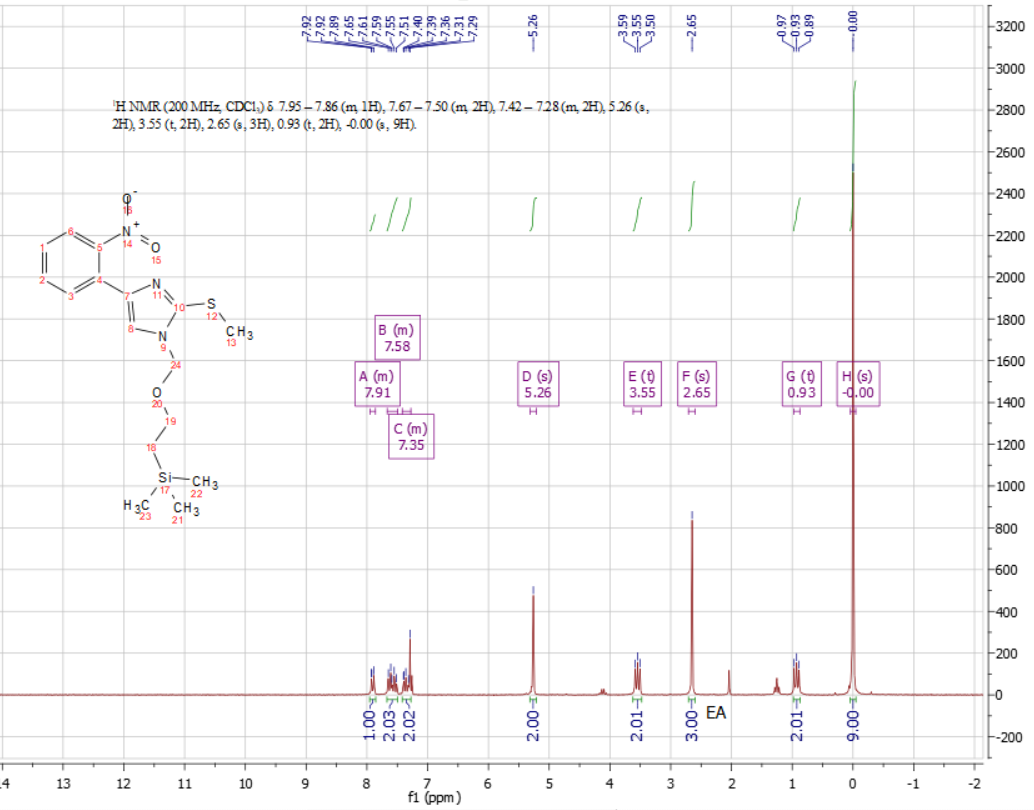


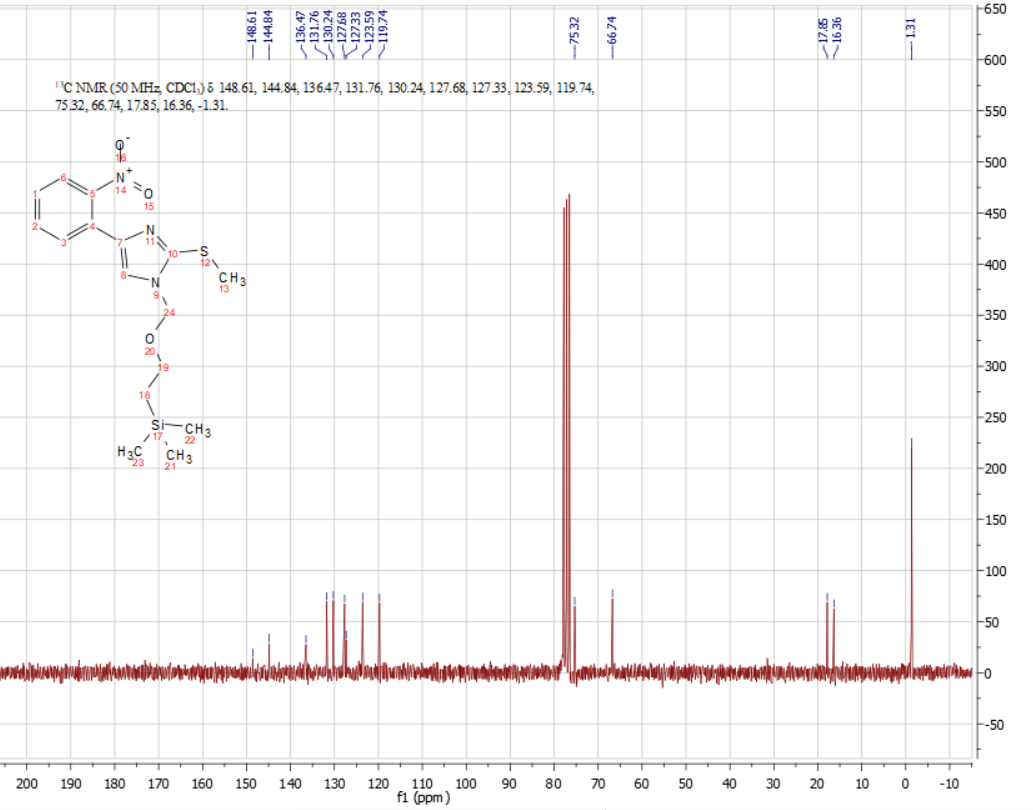


**S12**


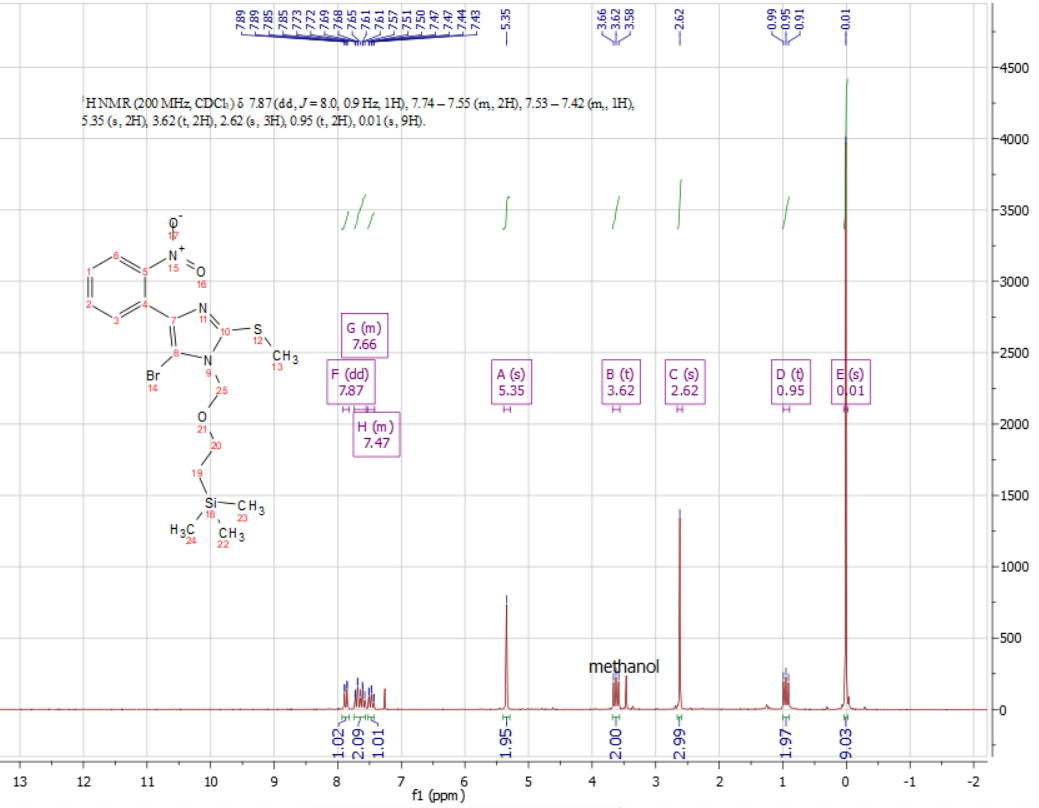


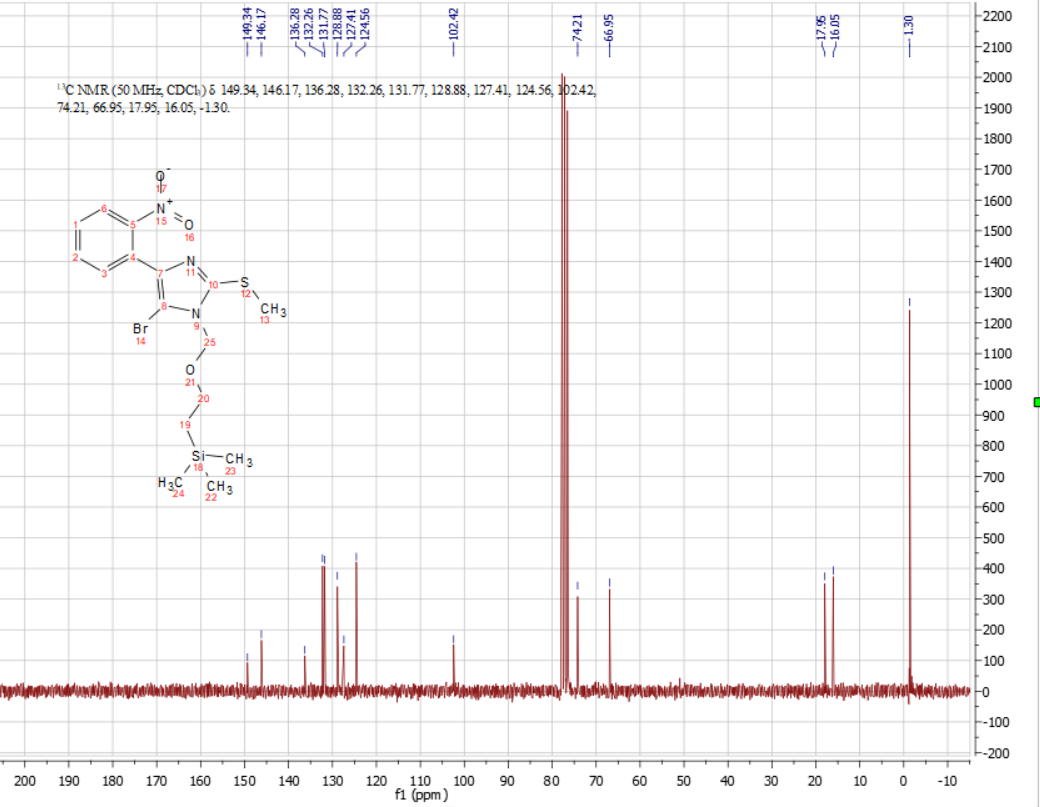


**S13**


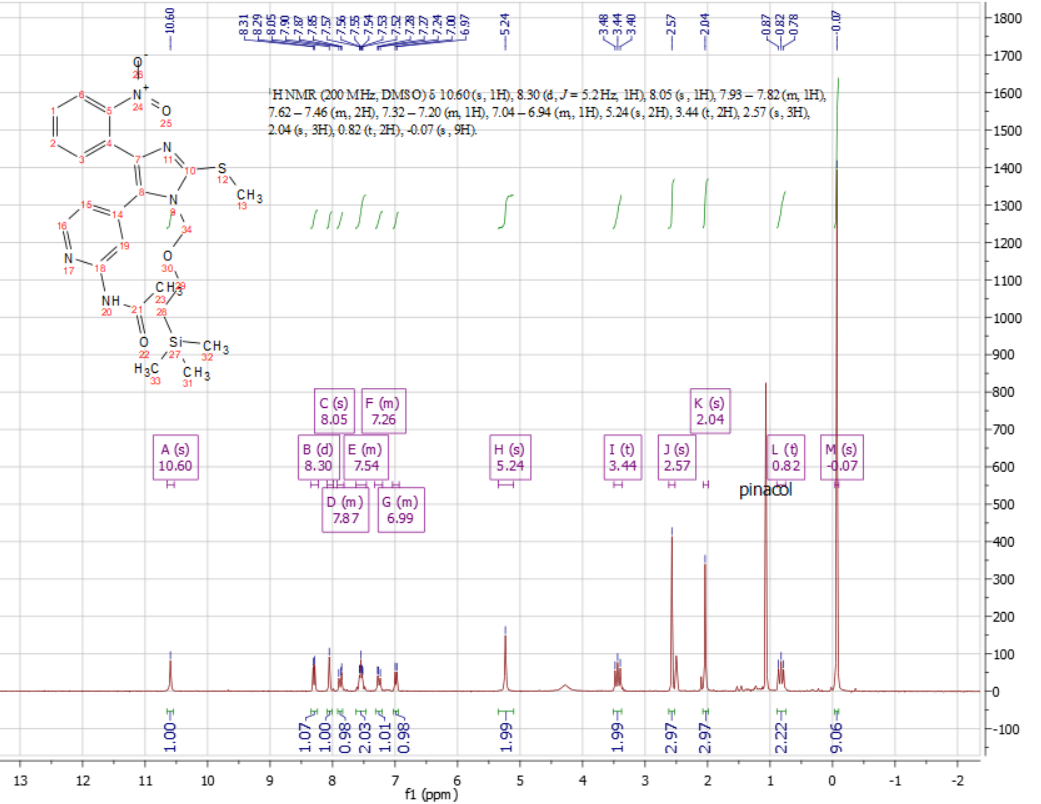


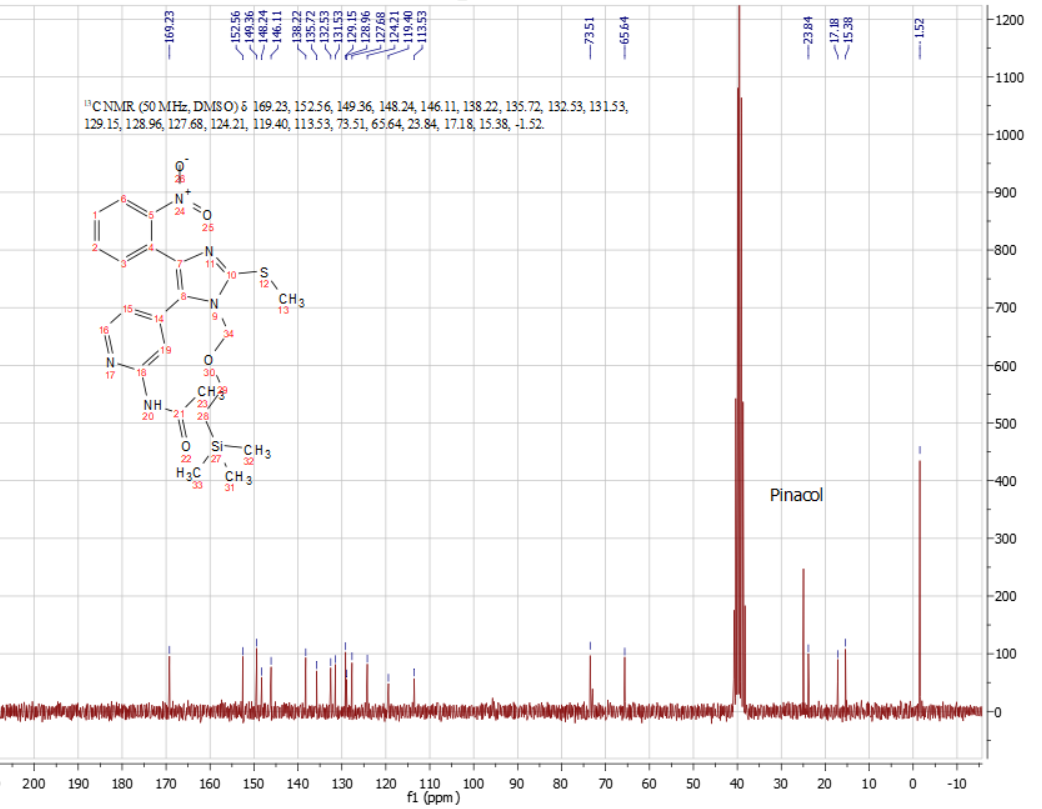


**S14**


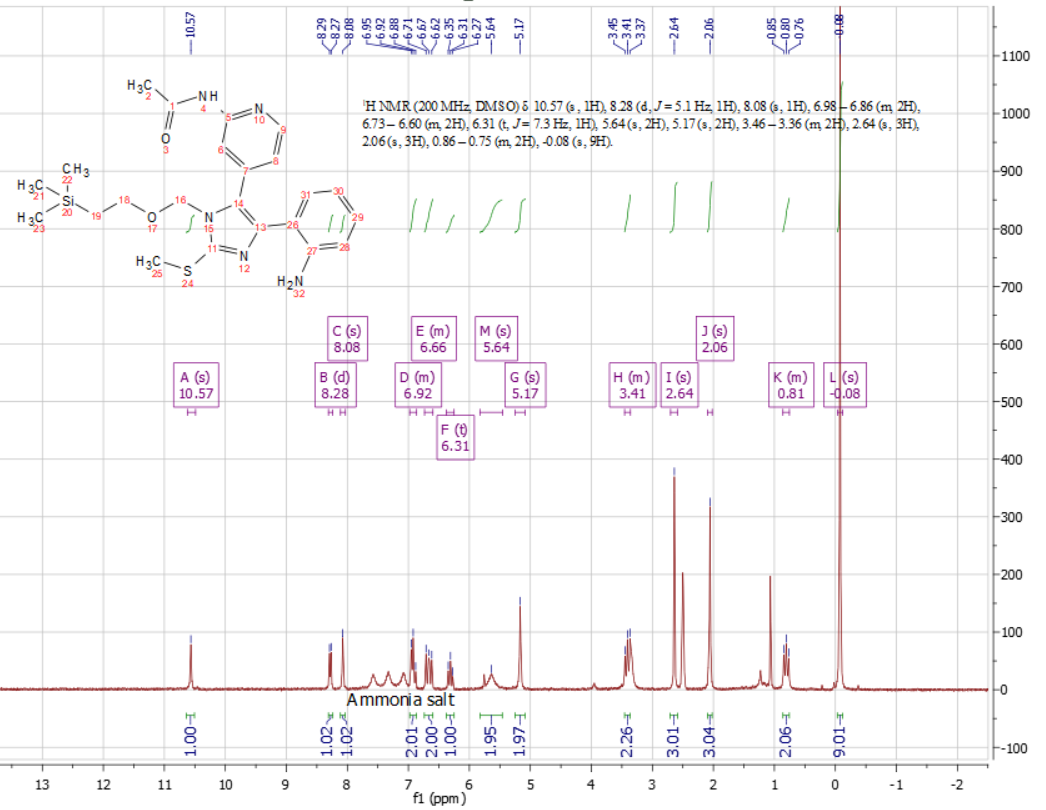


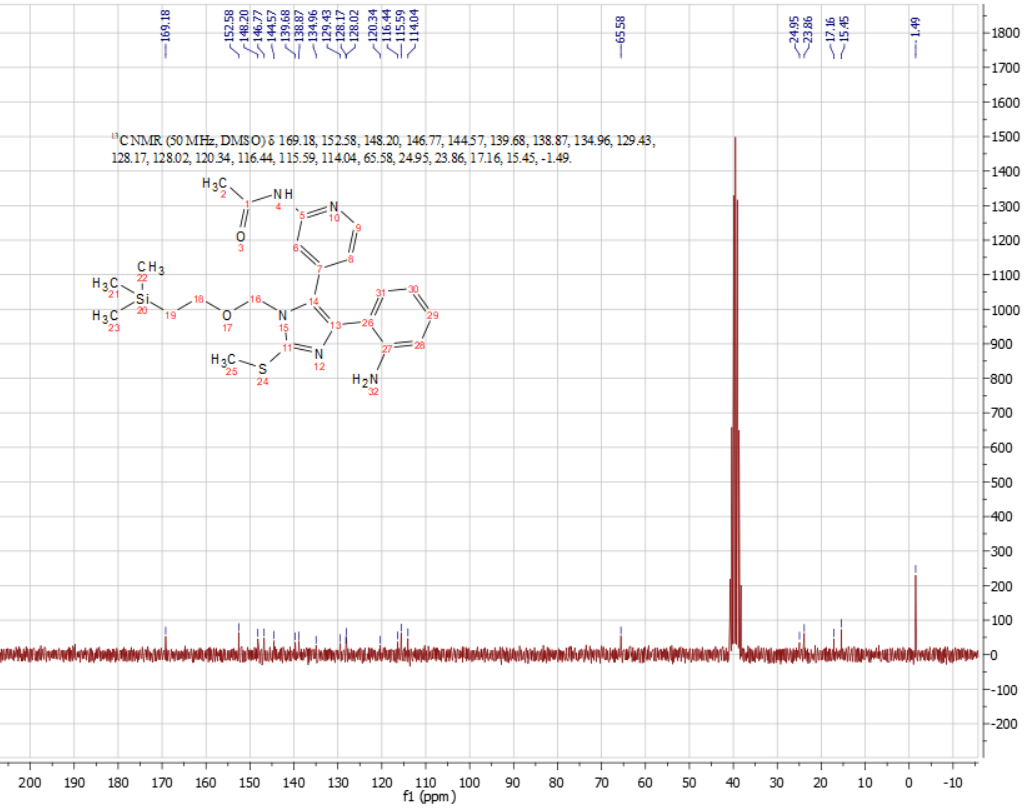


**2**

Compound


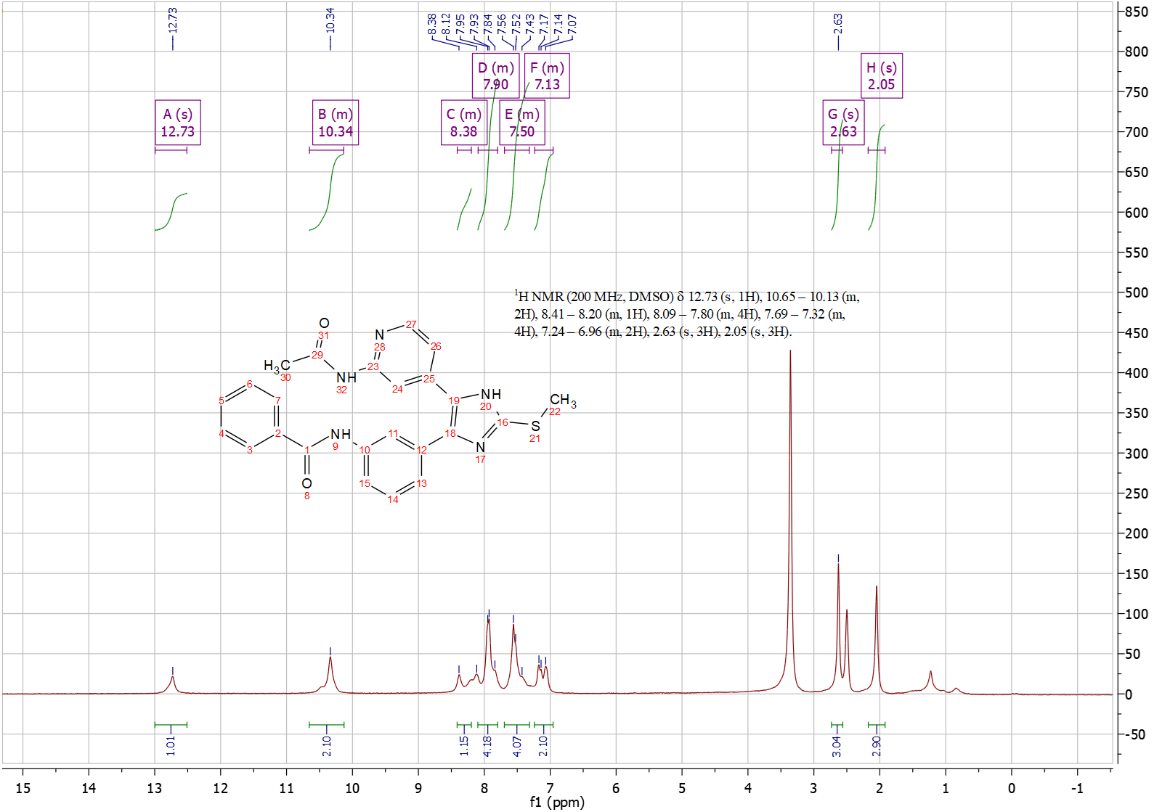


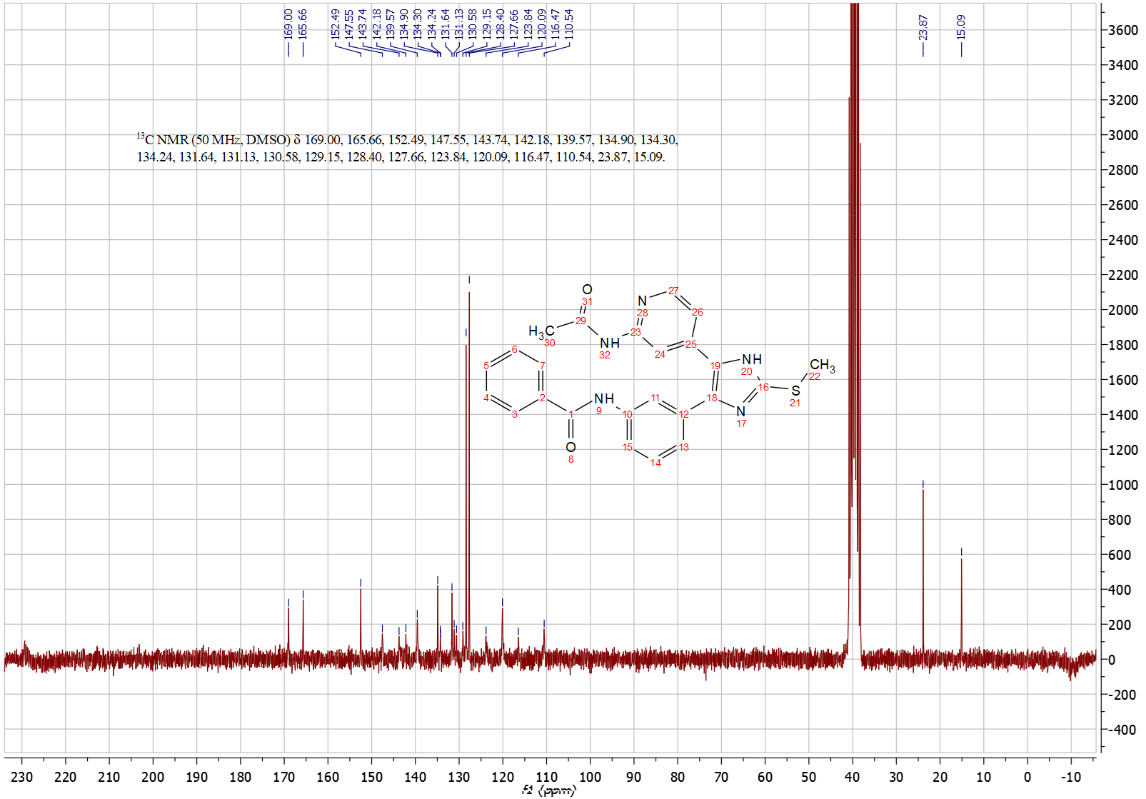


**3**

Intermediate


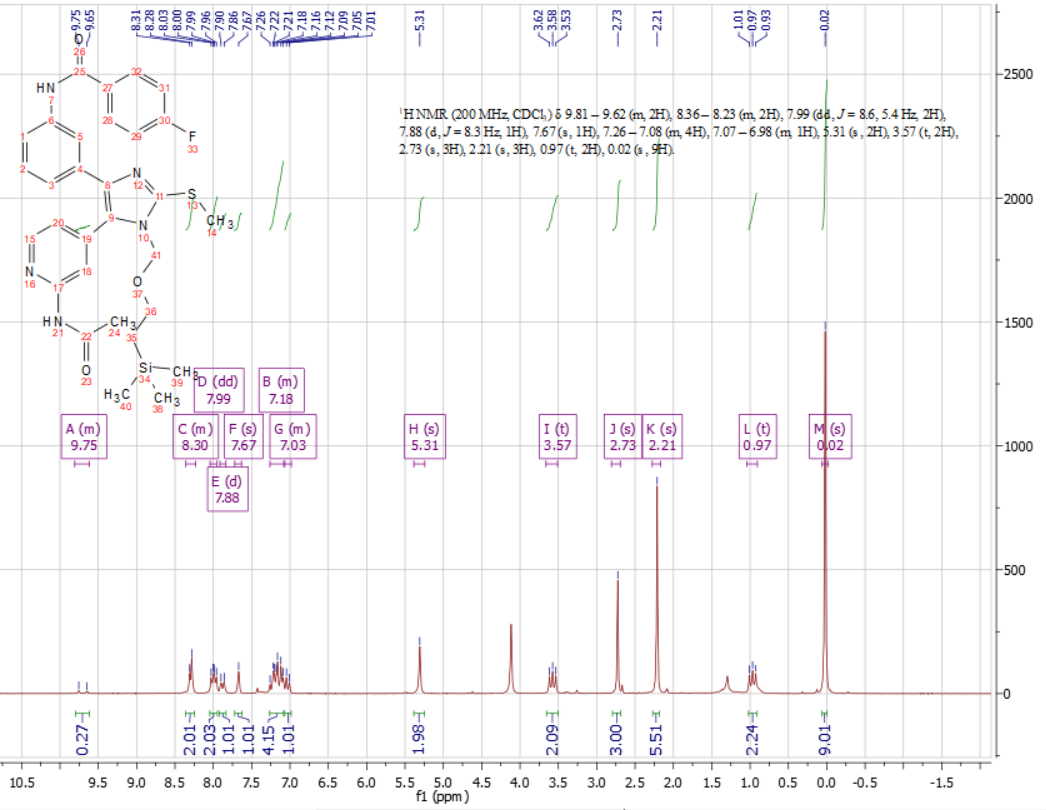


Compound


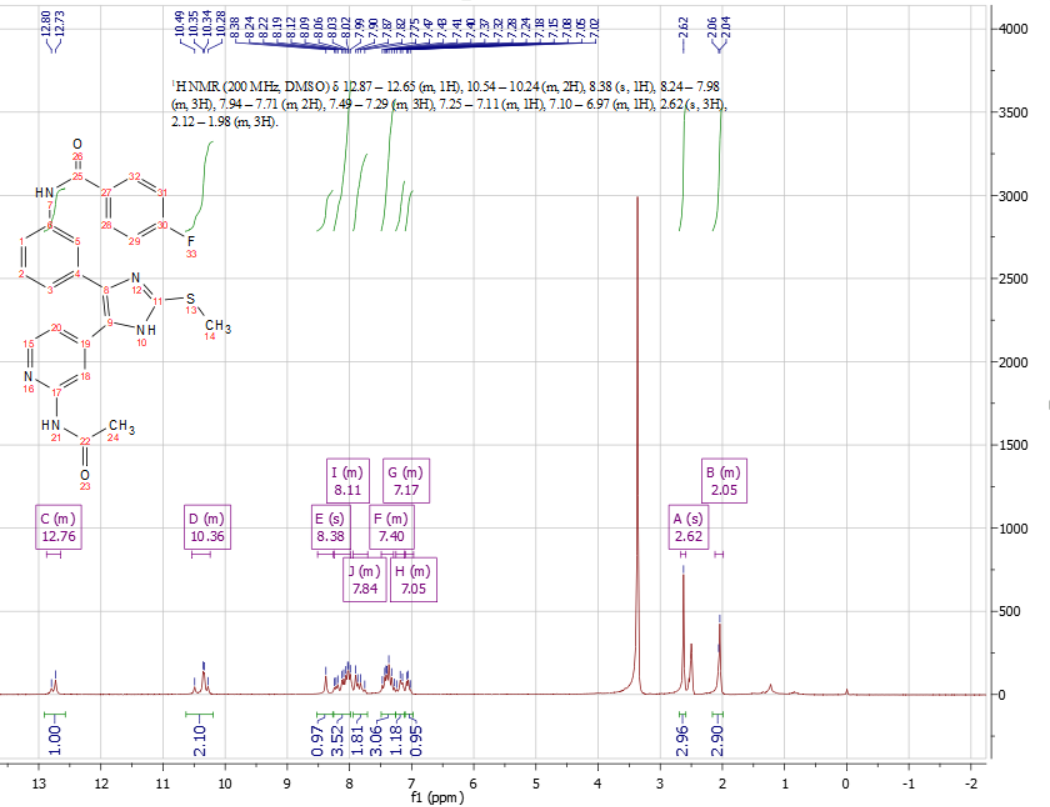


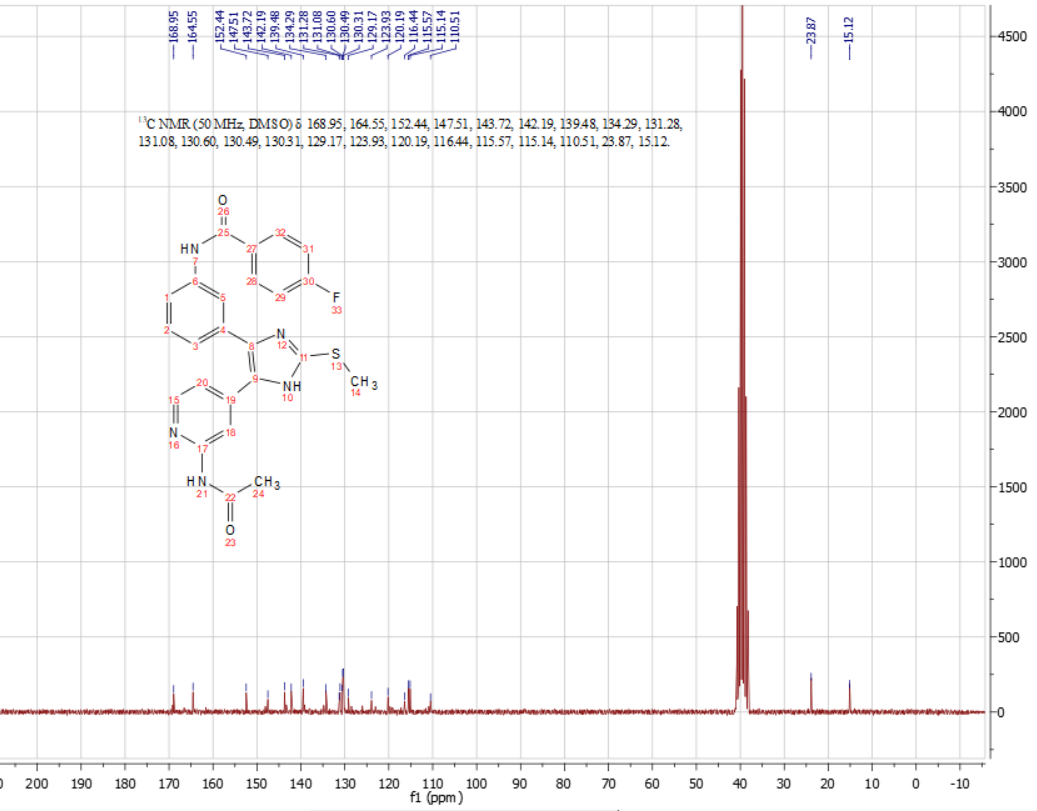


**4**

Intermediate


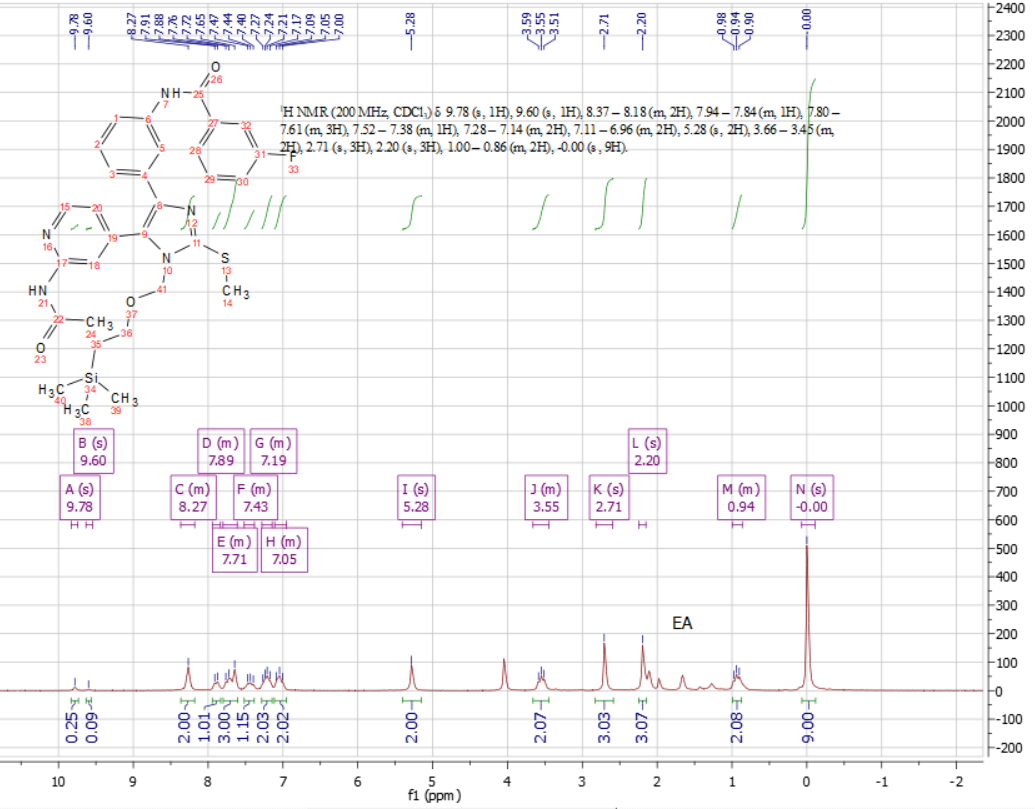


Compound


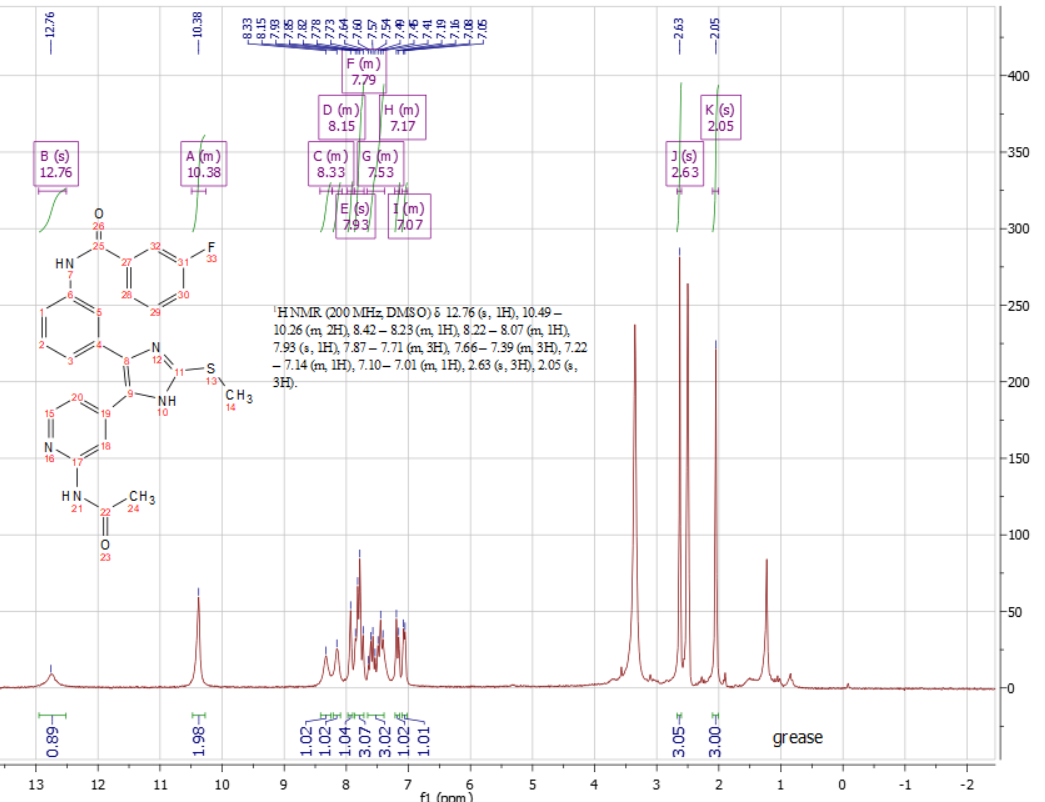


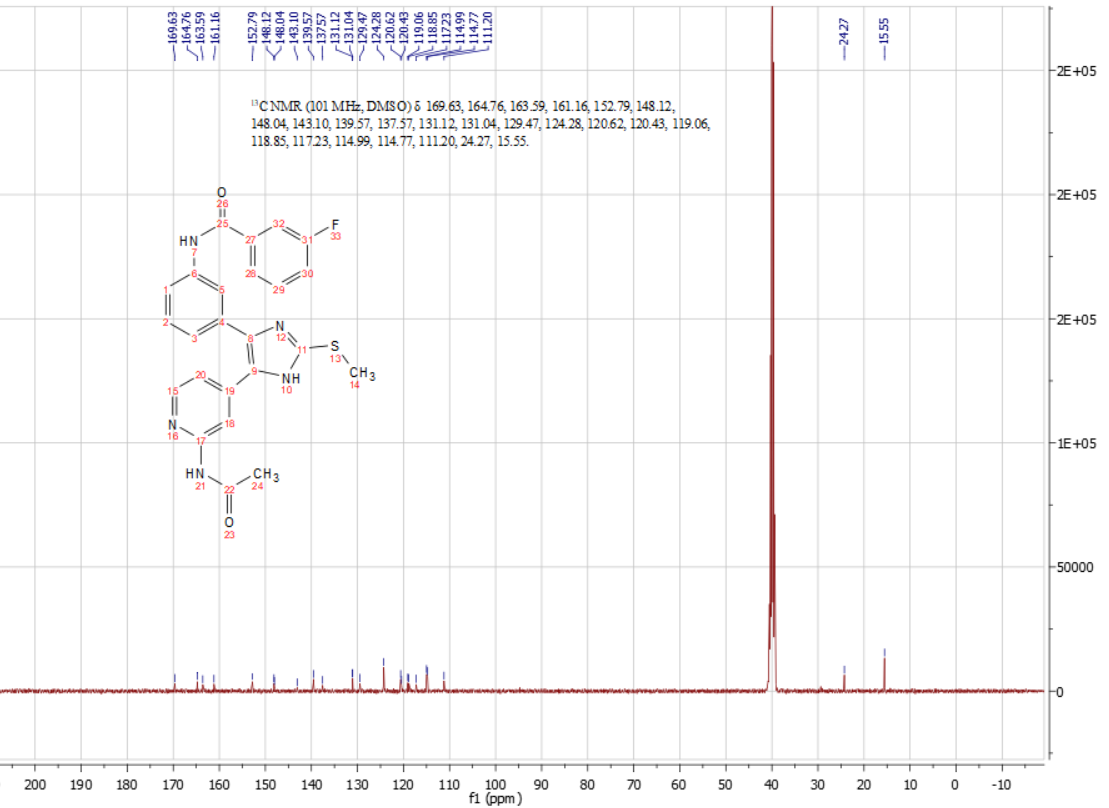


**5**

Intermediate


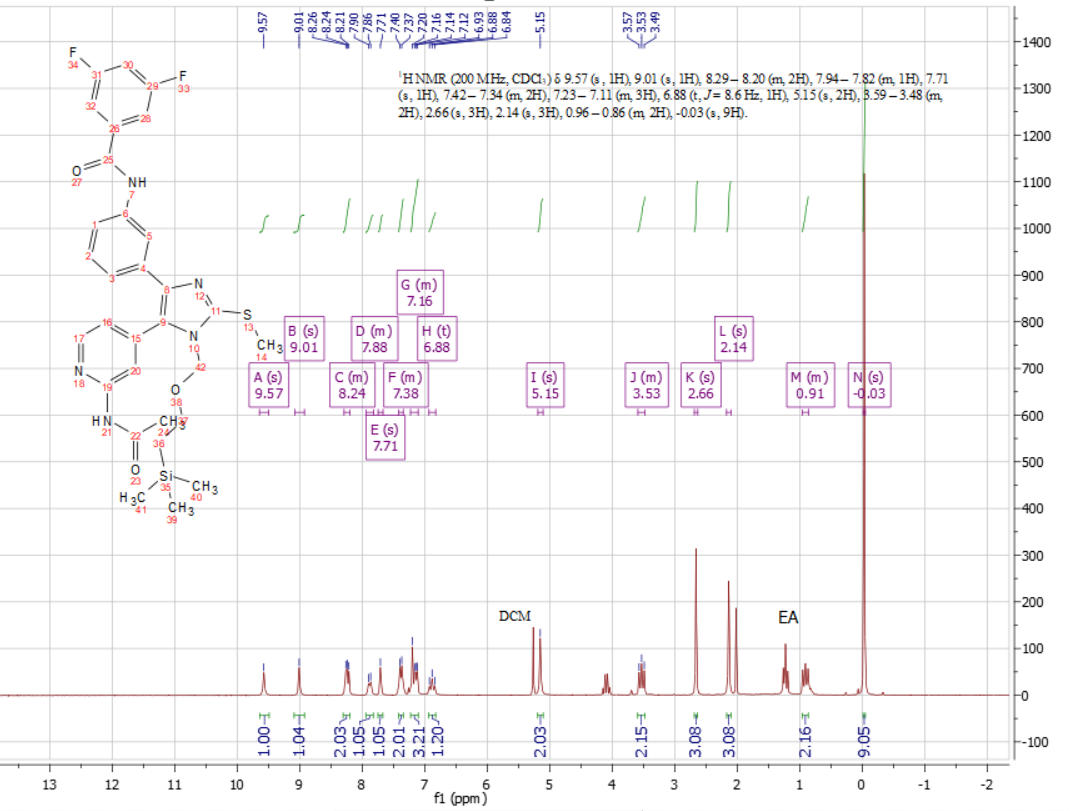


Compound


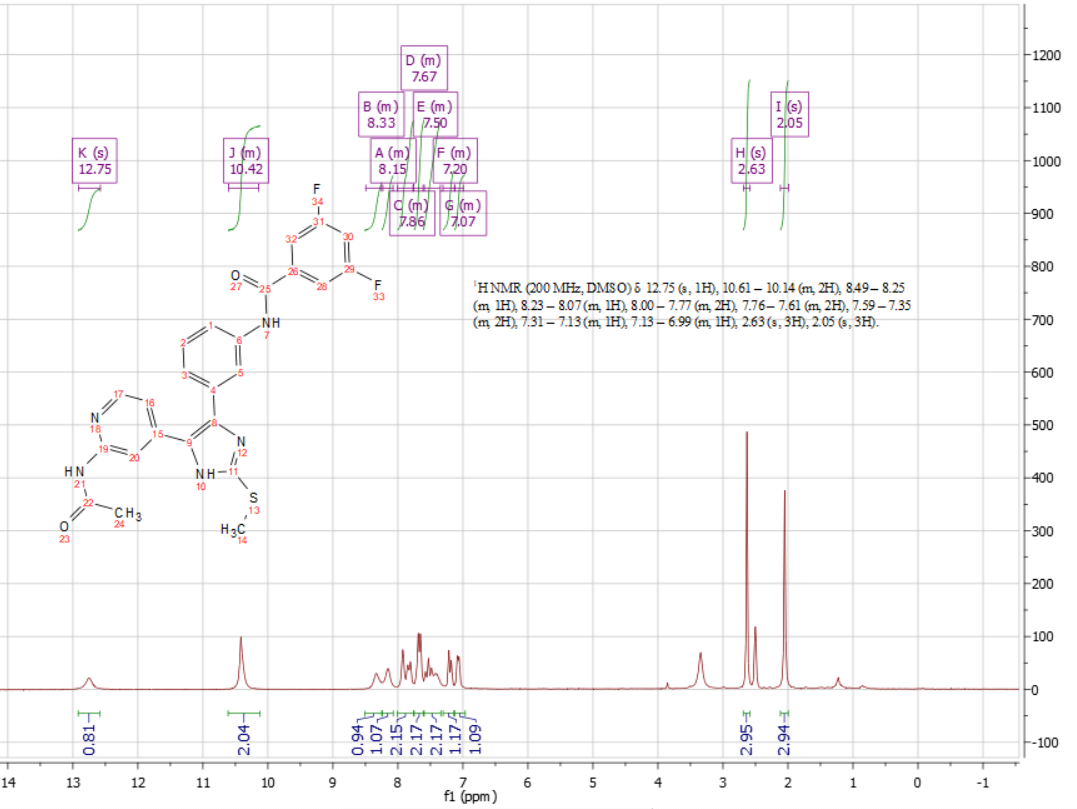


**6**

Compound


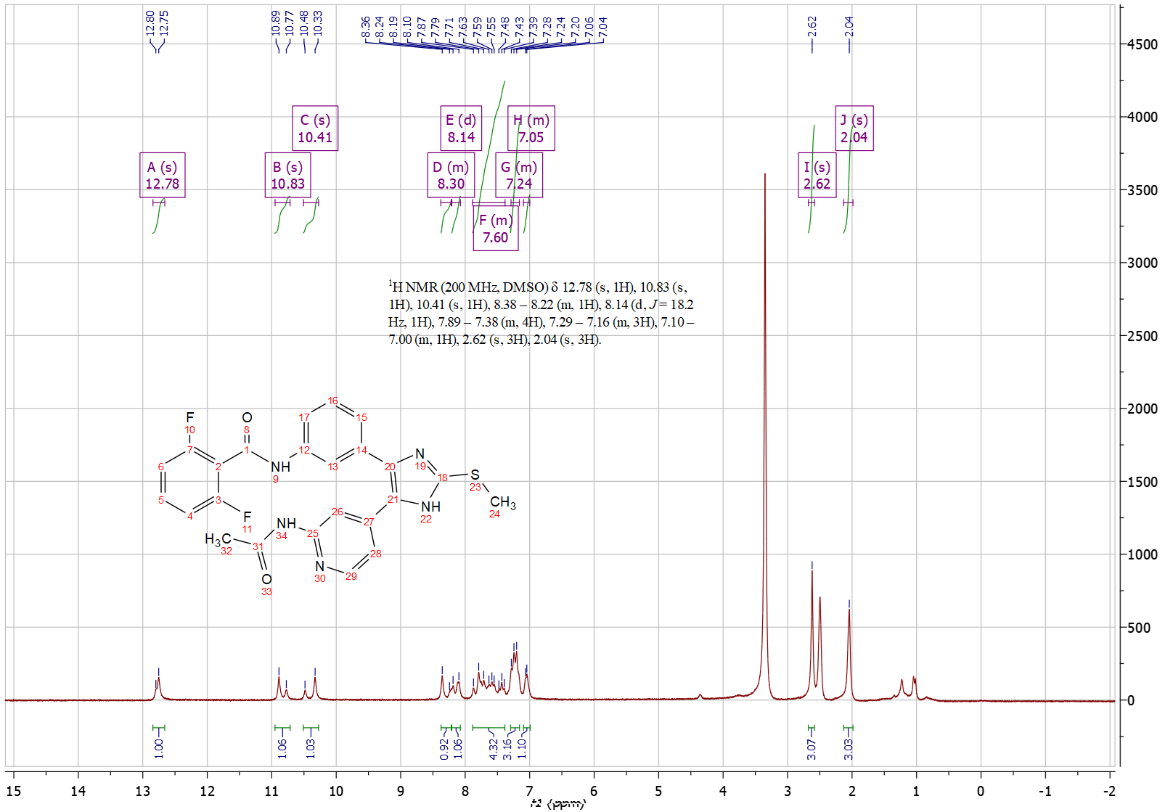


**7**

Compound


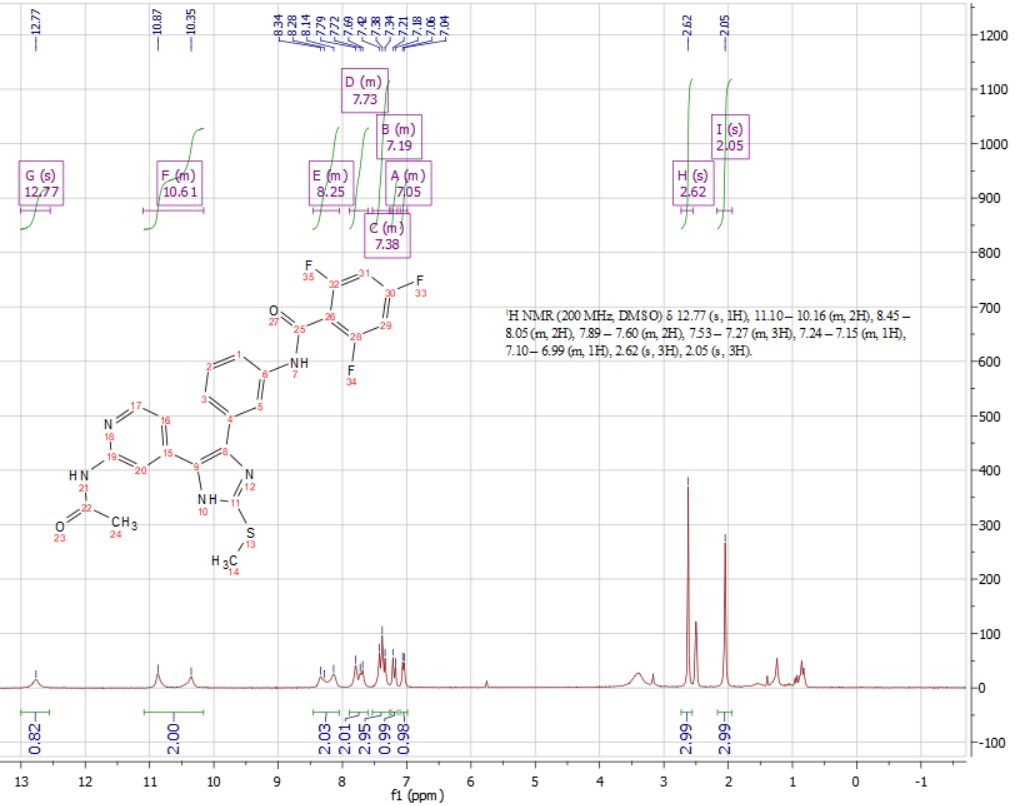


**10**

Intermediate


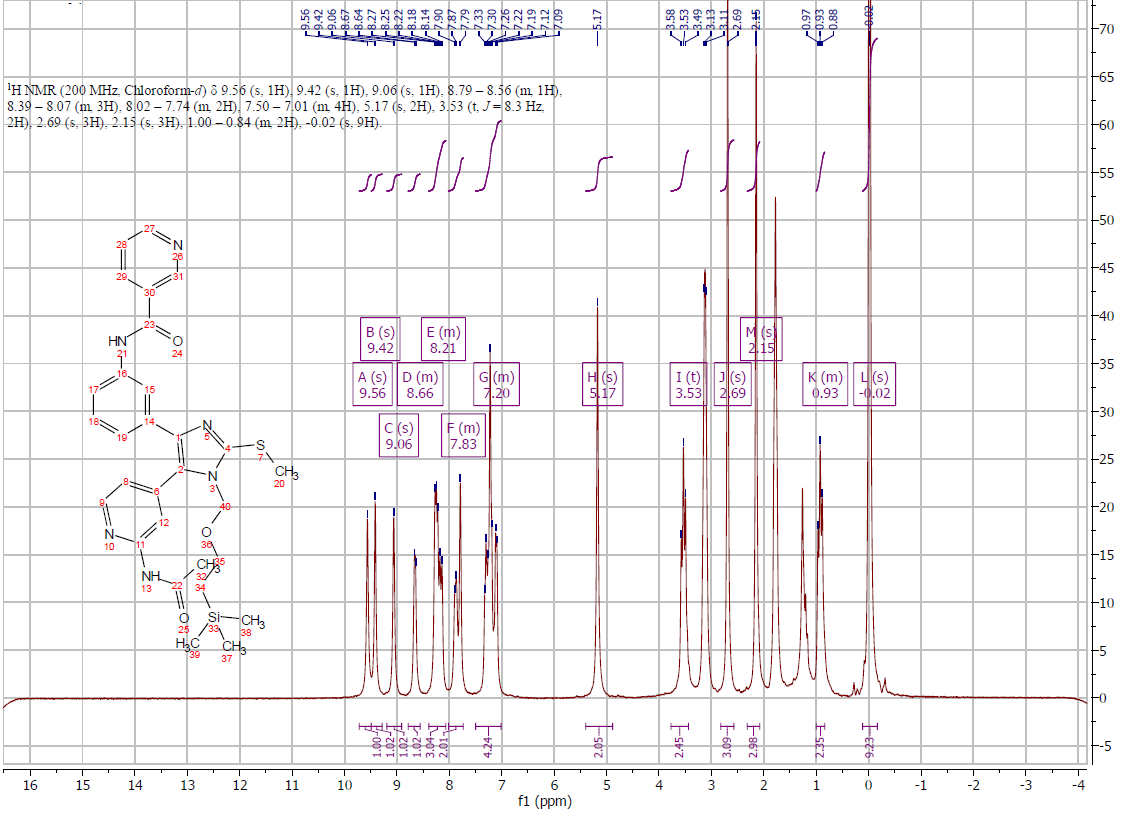


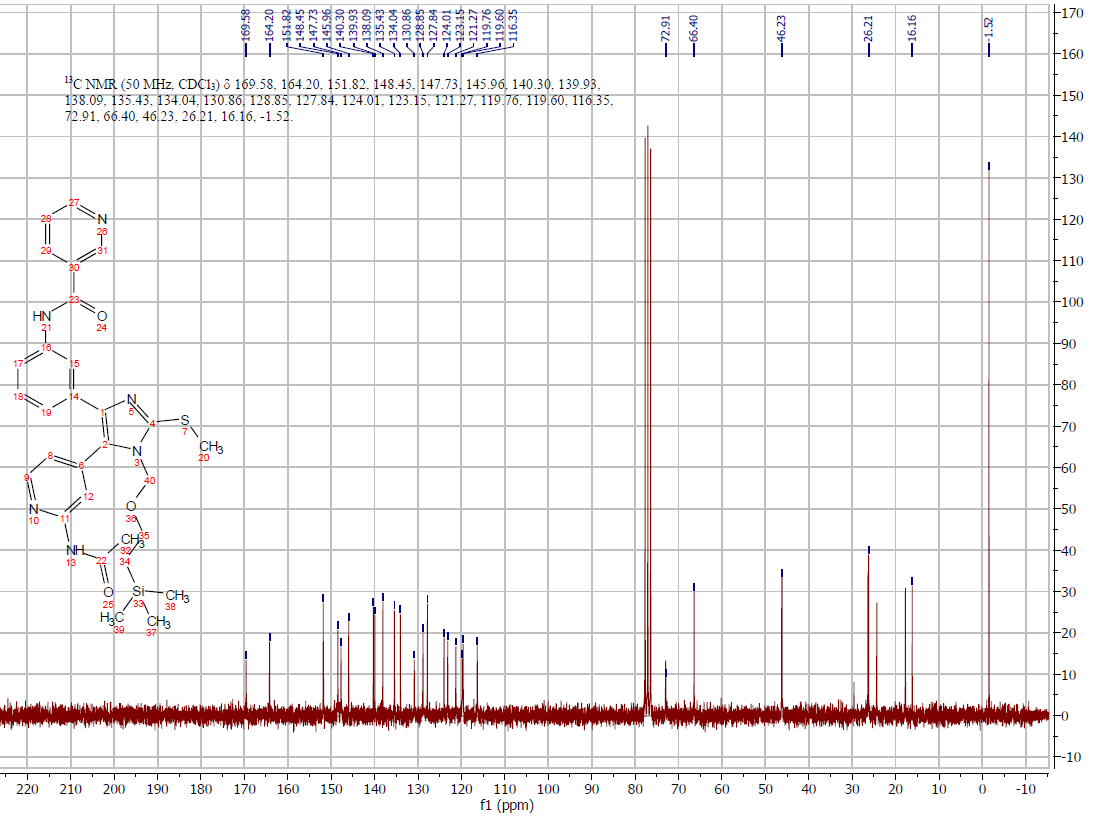


Compound


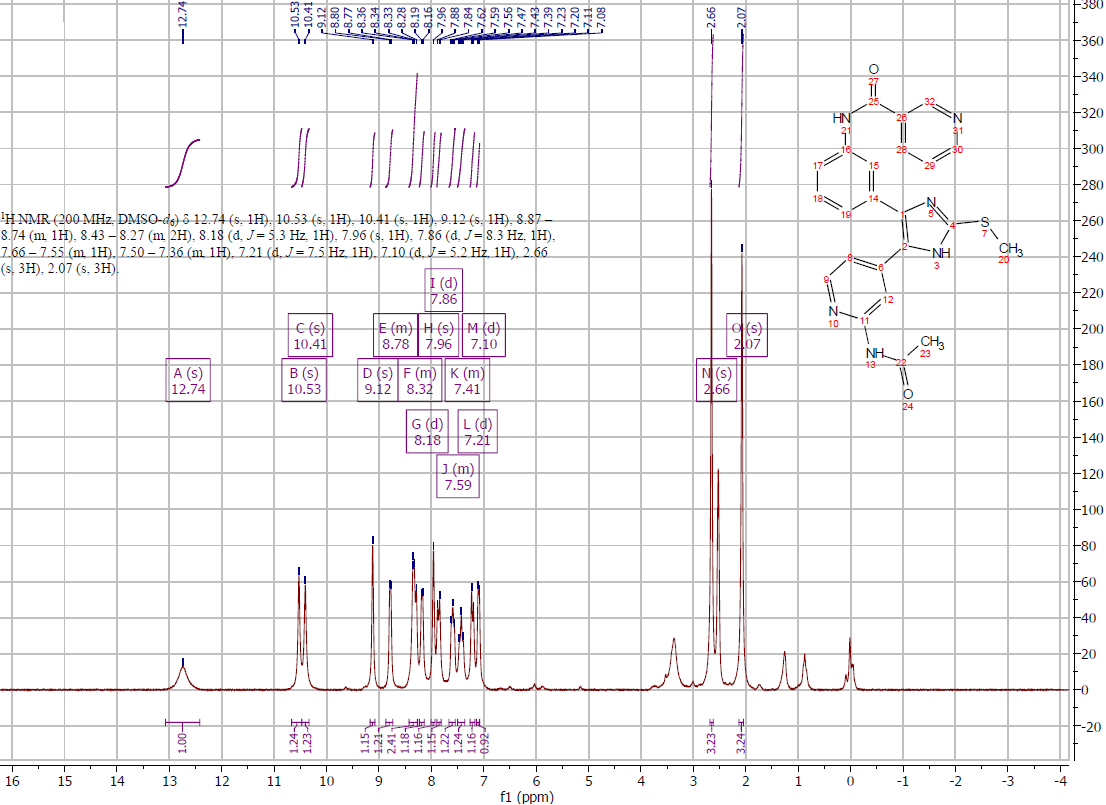


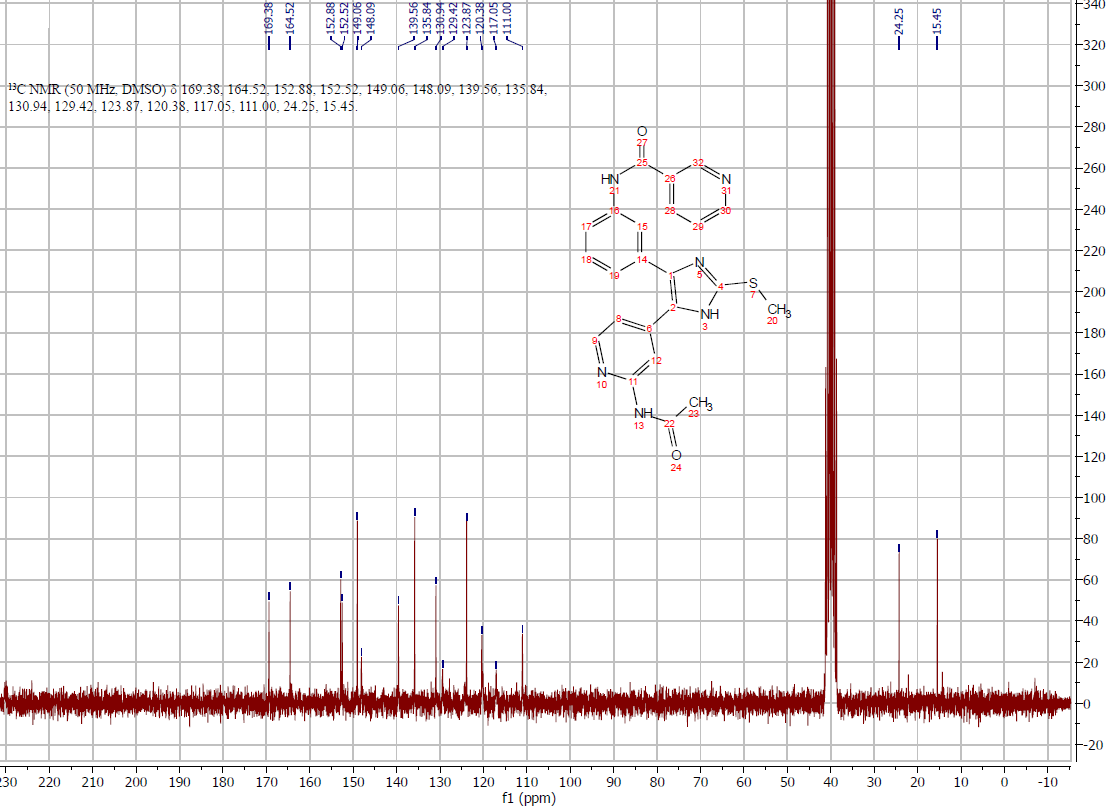


**11**

Intermediate


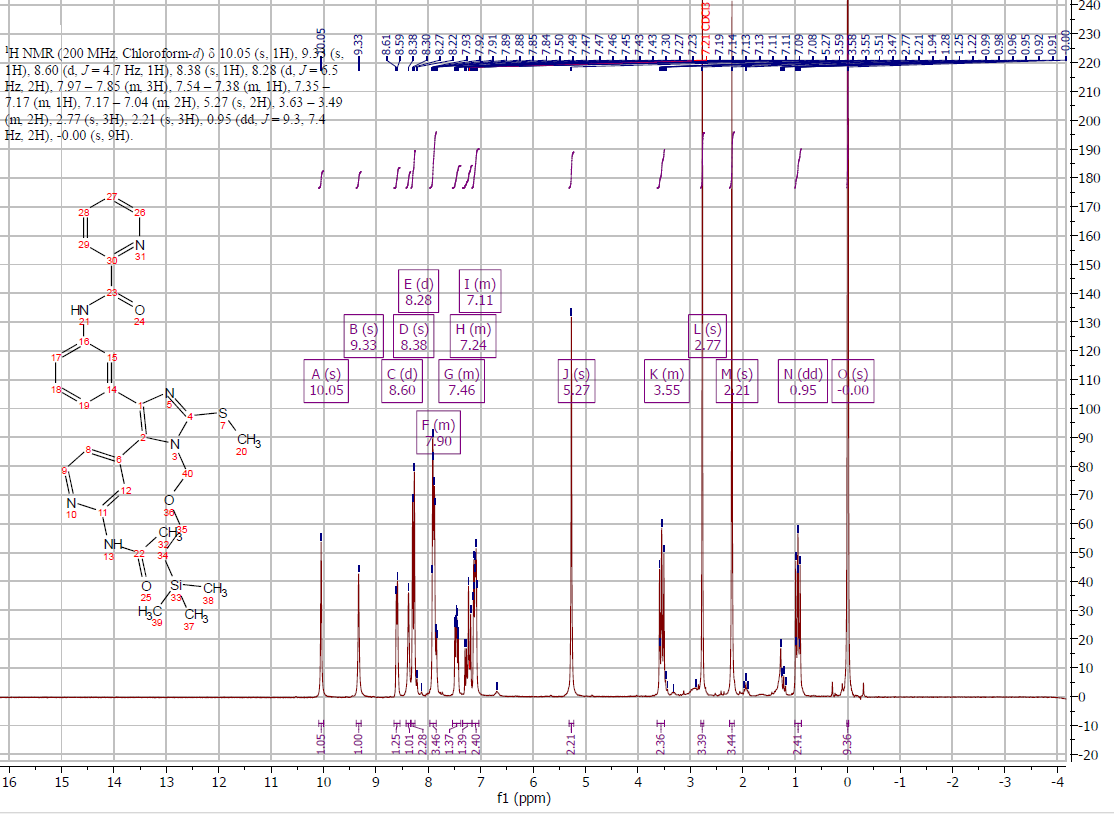


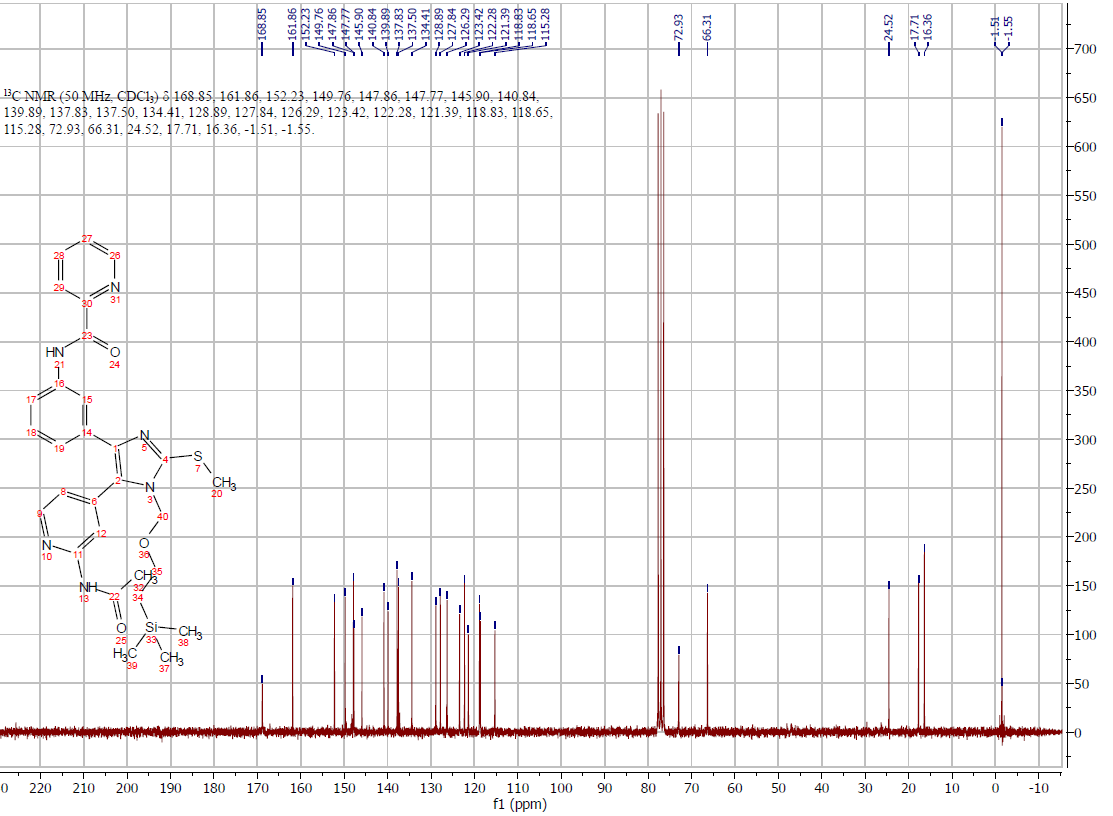


Compound


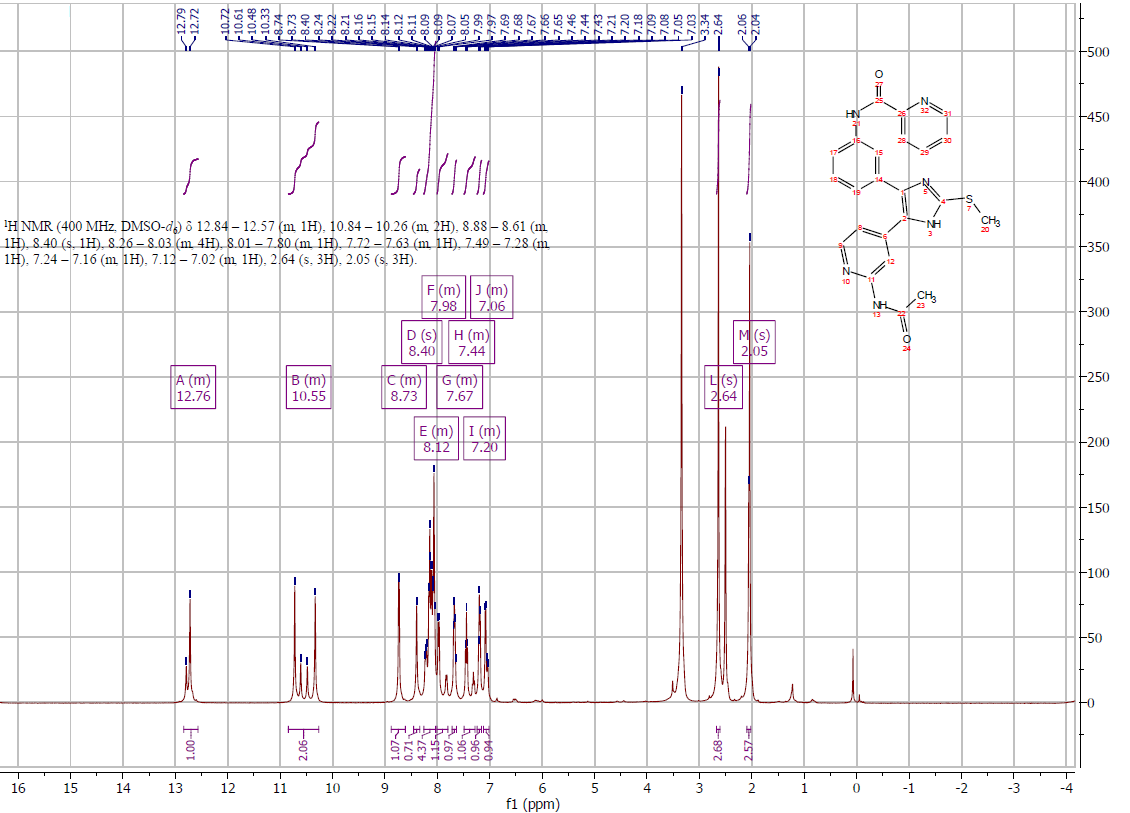


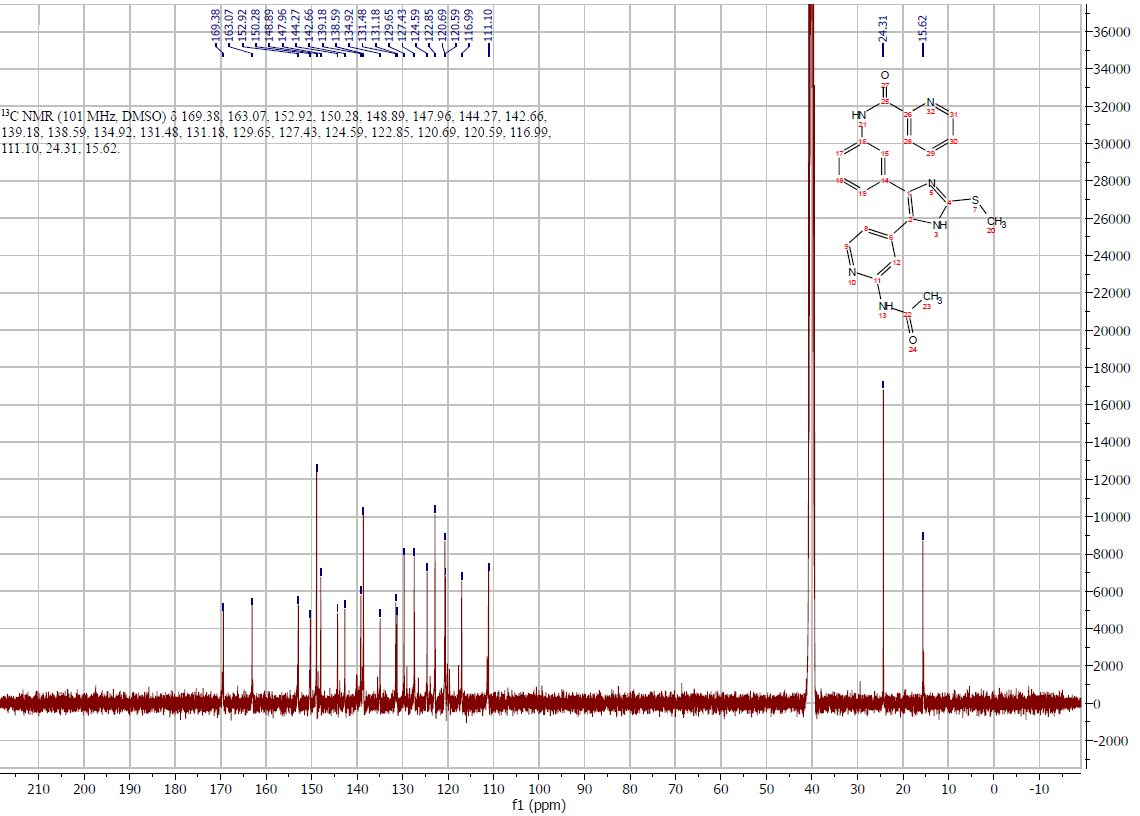


**12**

Intermediate


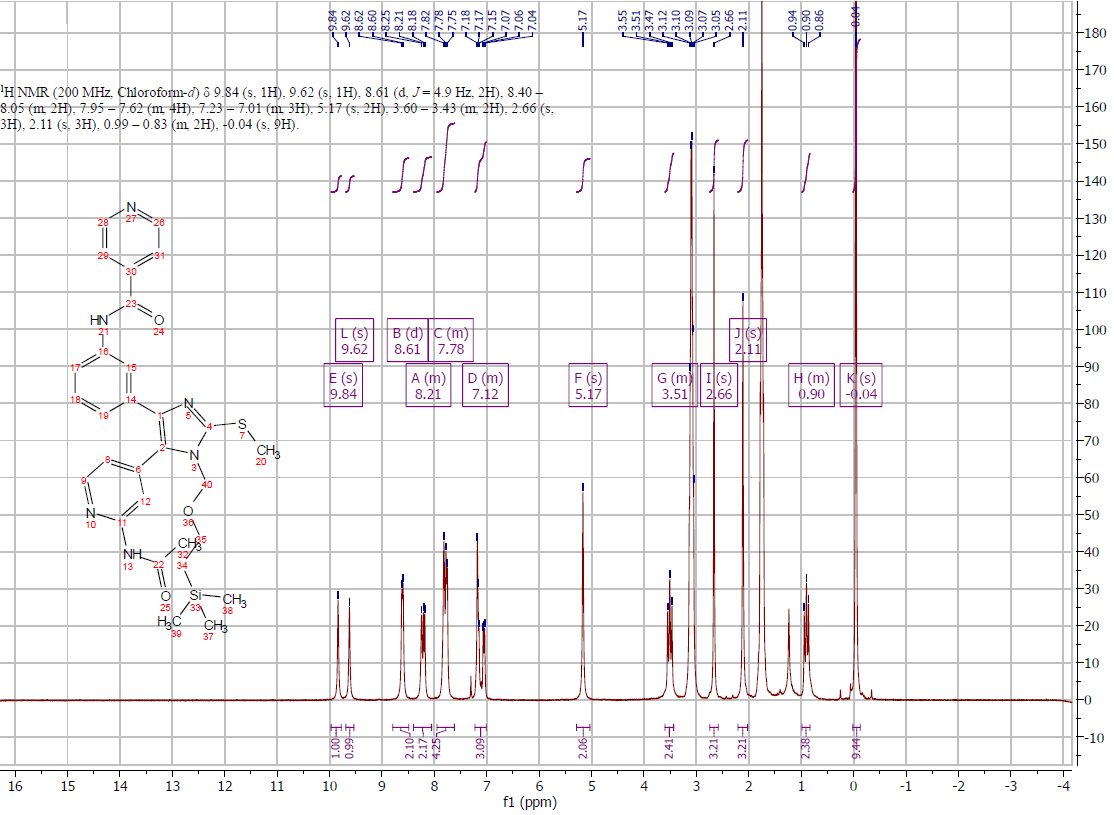


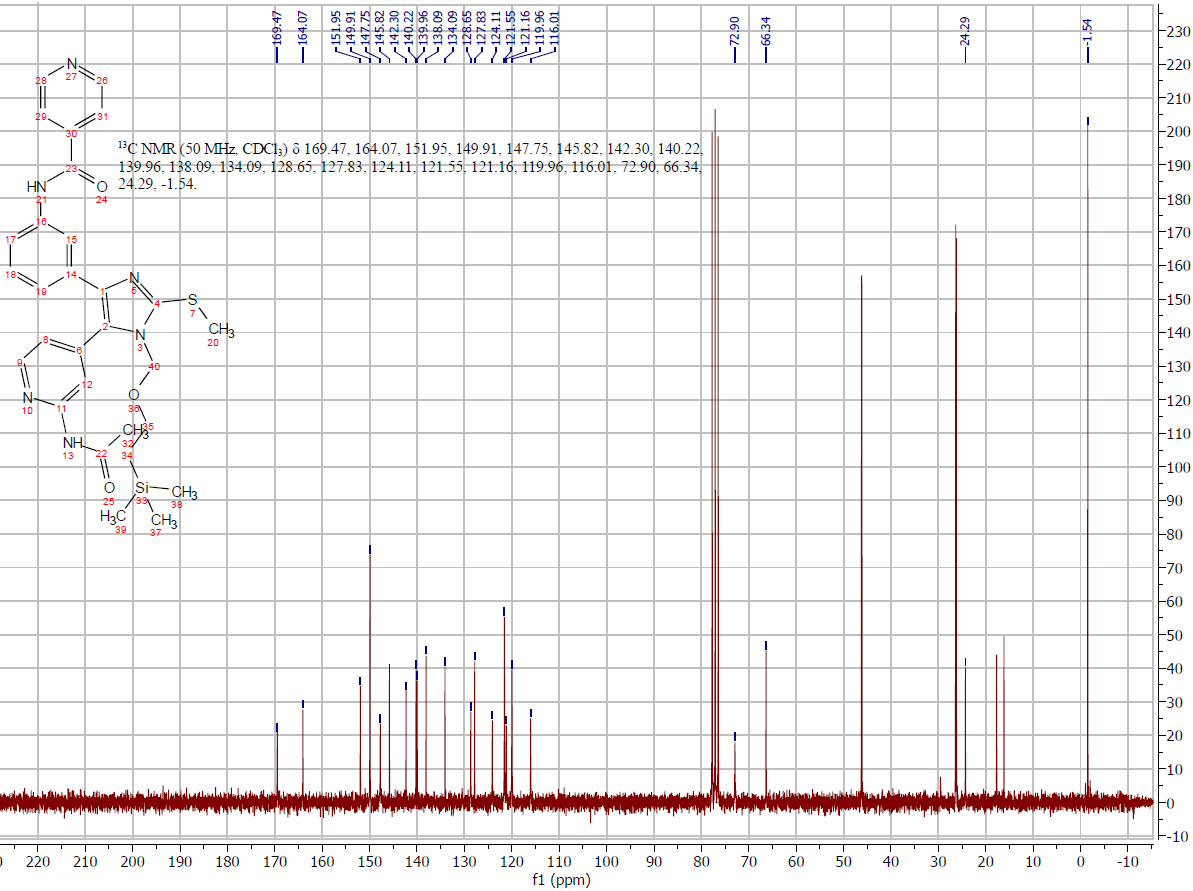


Compound


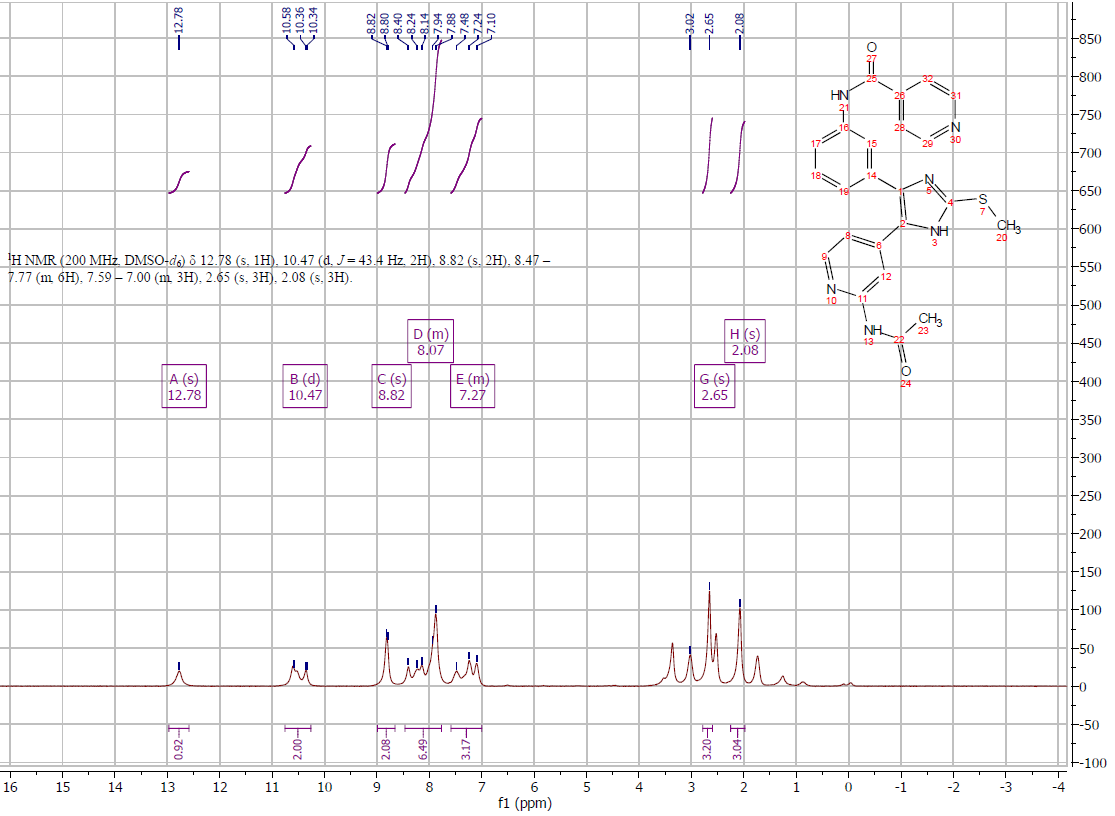


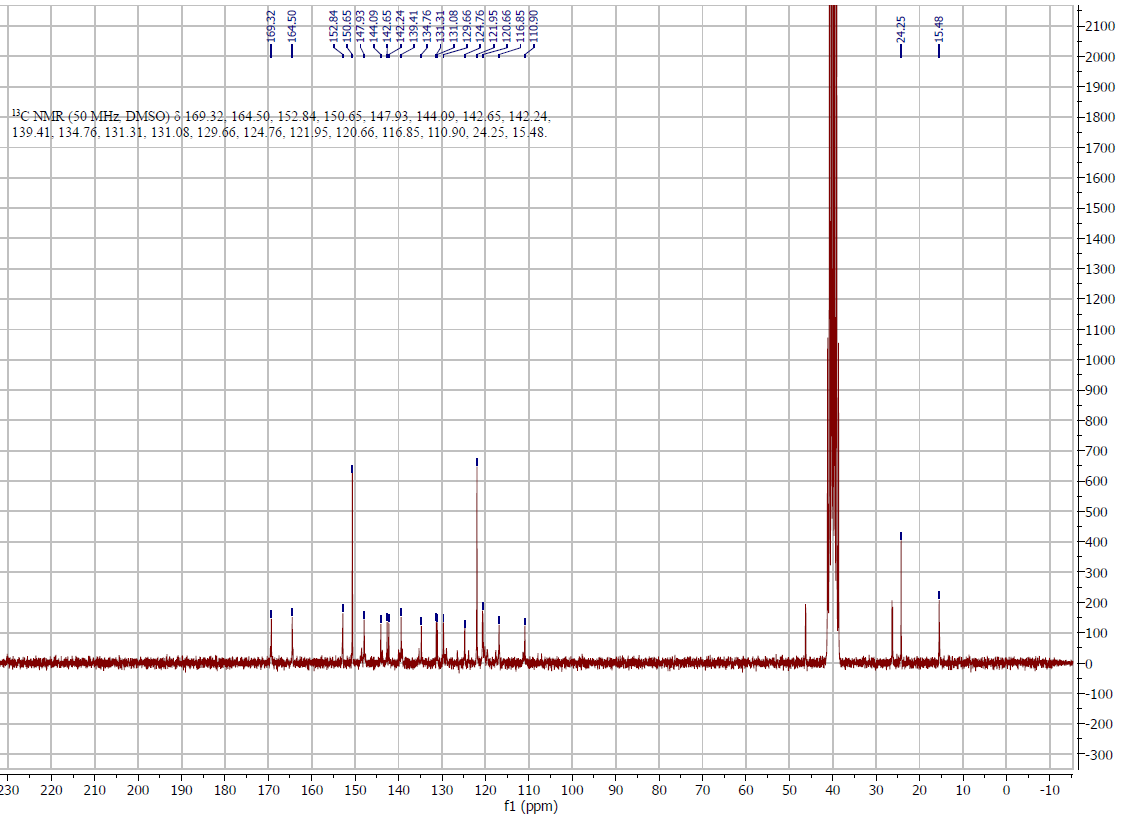


**13**

Intermediate


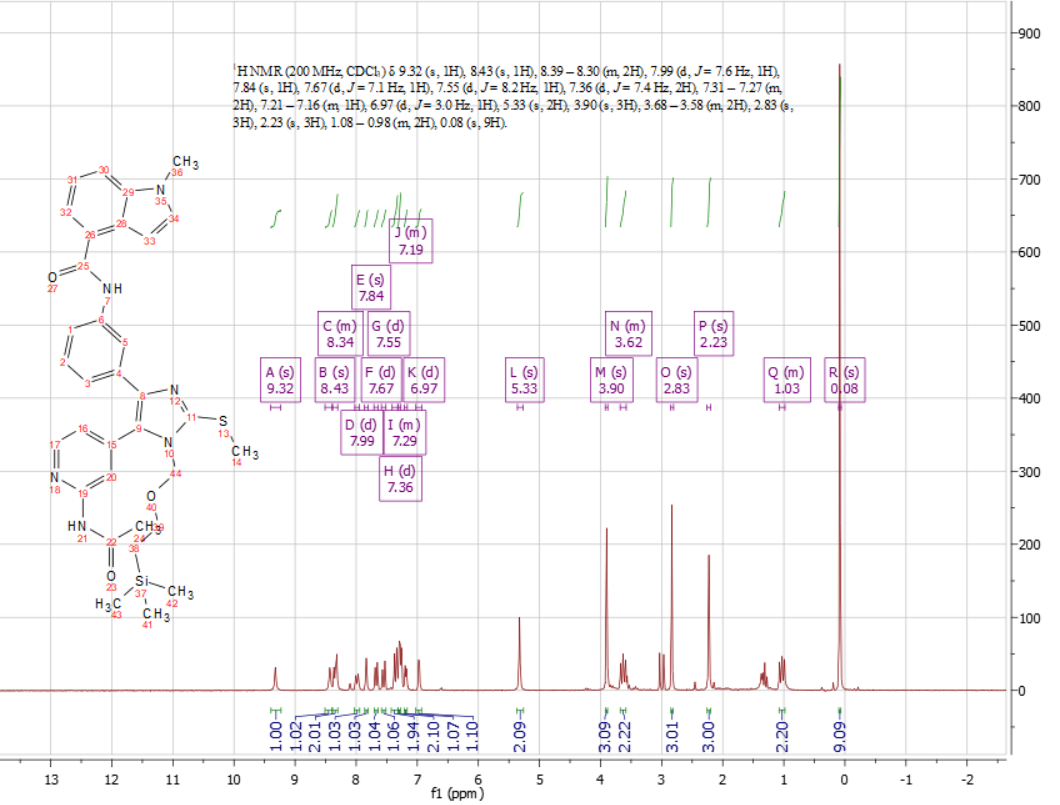


Compound


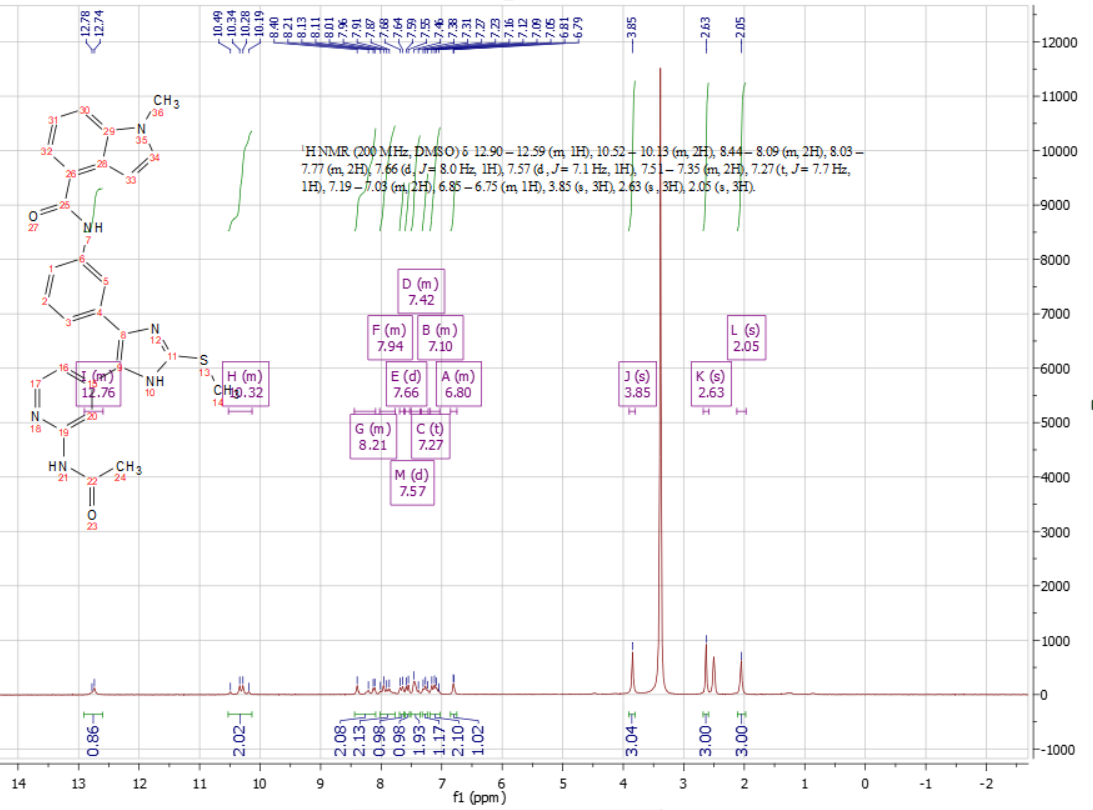


**14**

Compound


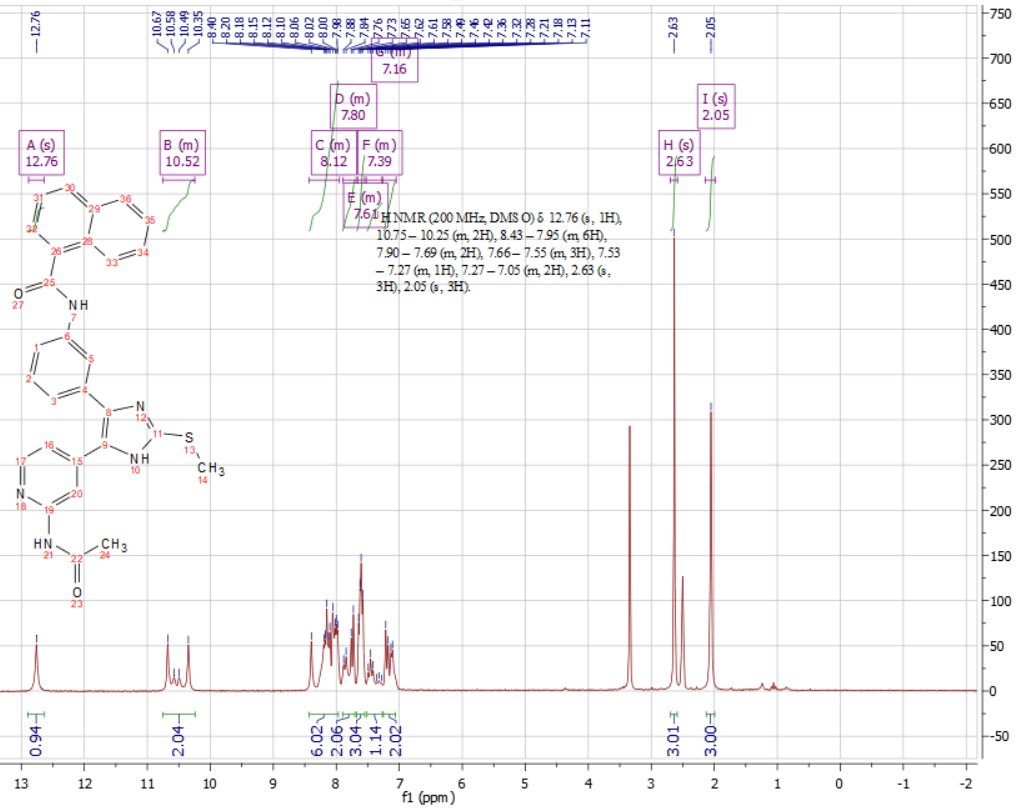


**15**

Compound


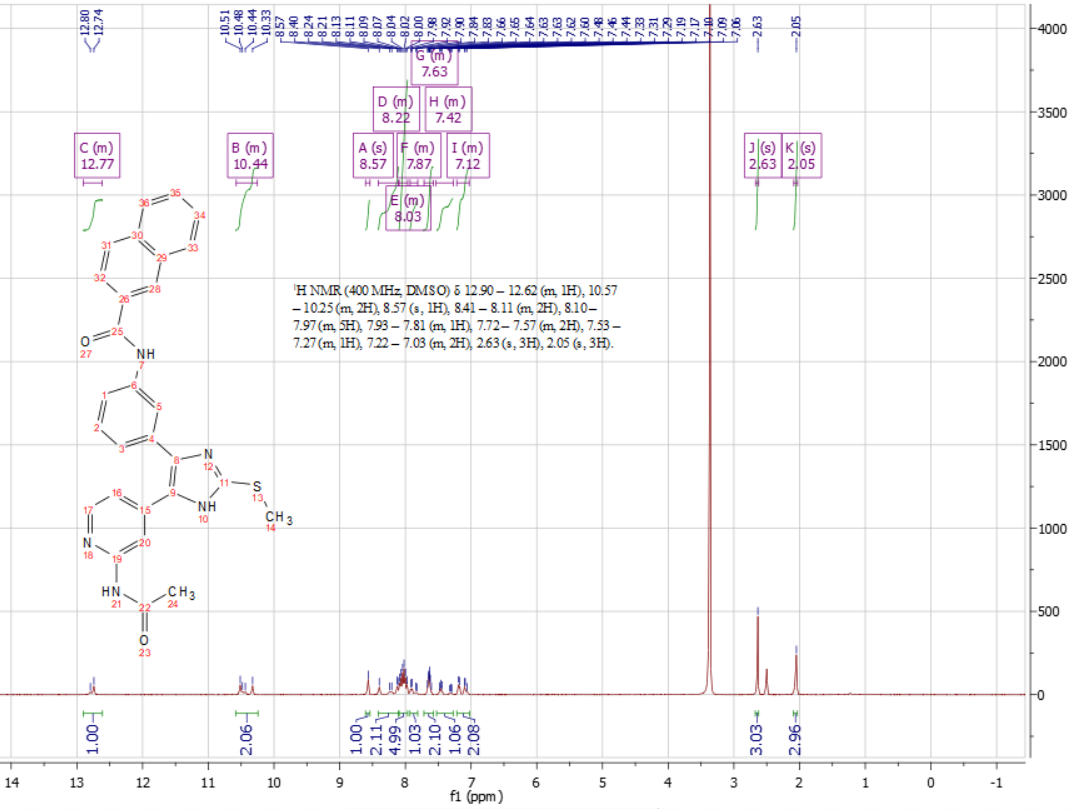


**16**

Compound


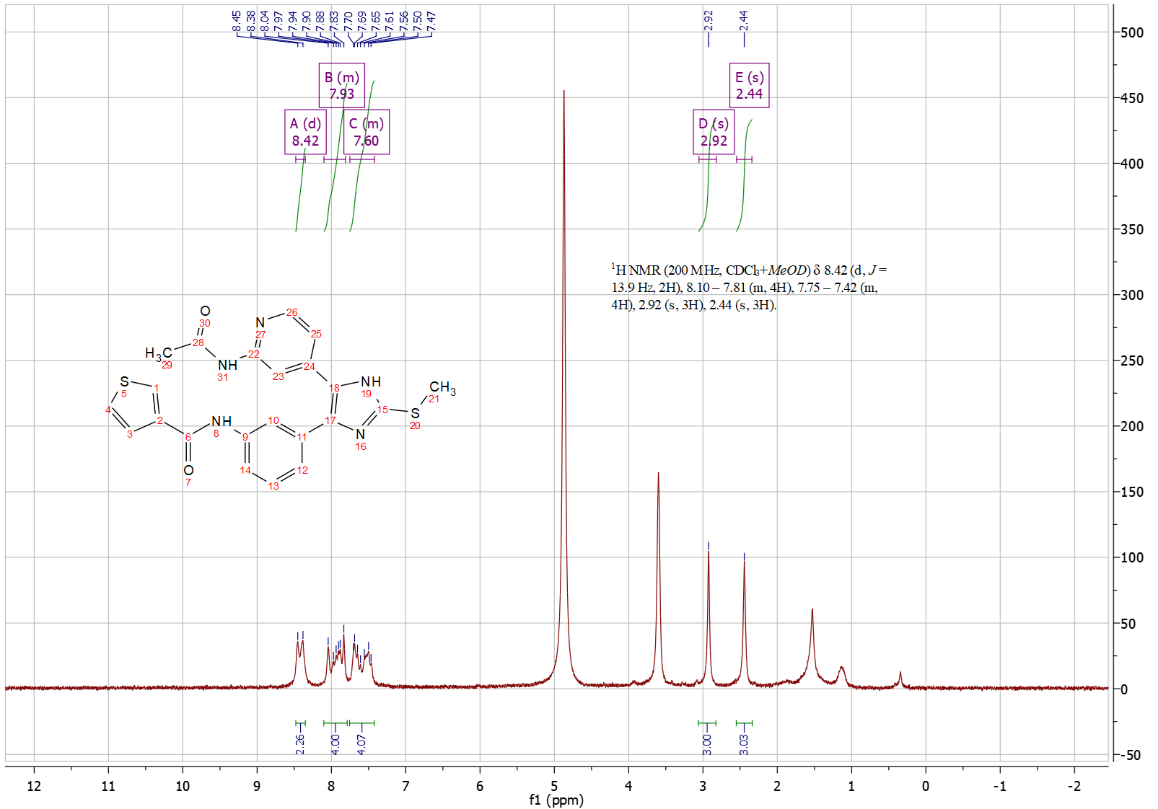


**17**

Intermediate


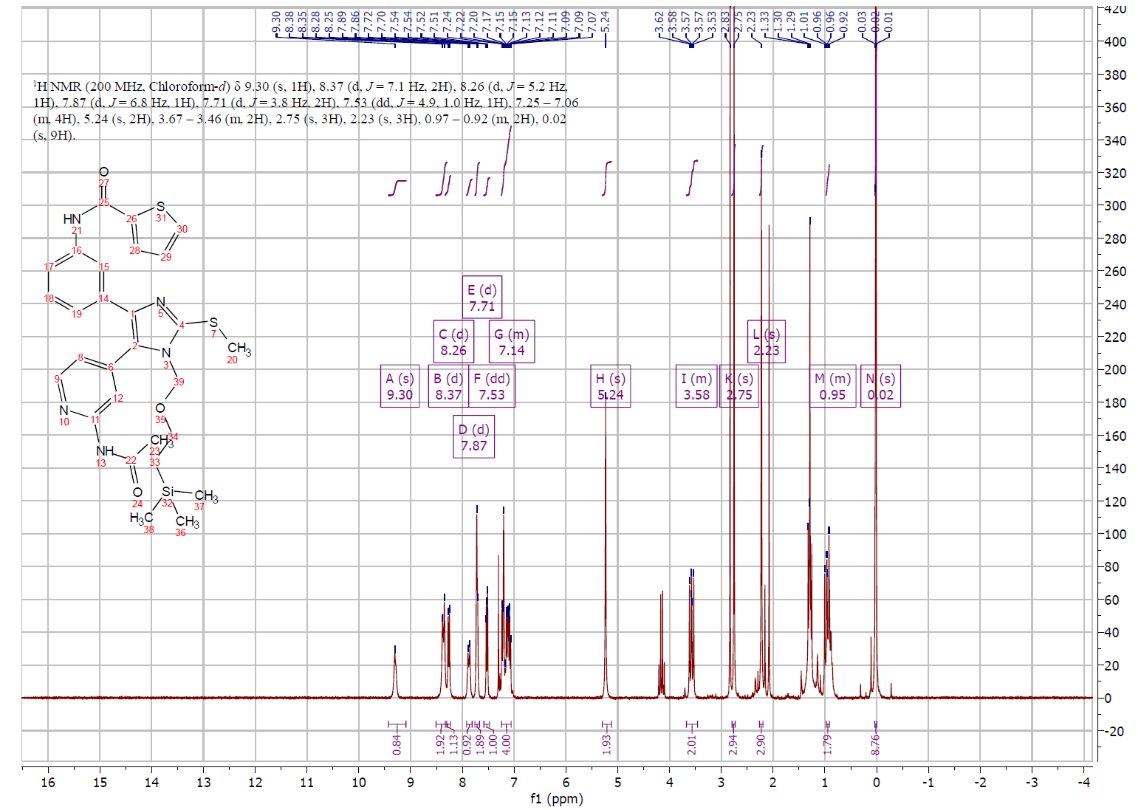


Compound


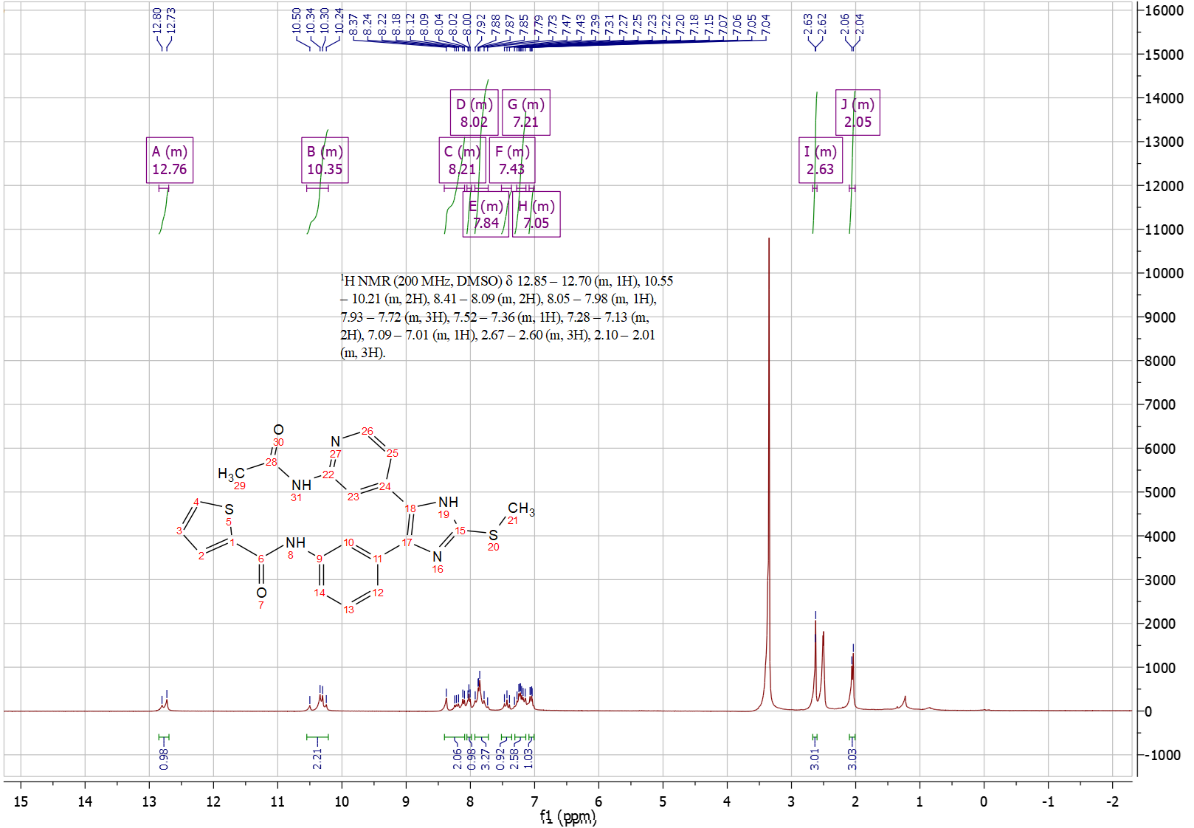


**18**

Compound


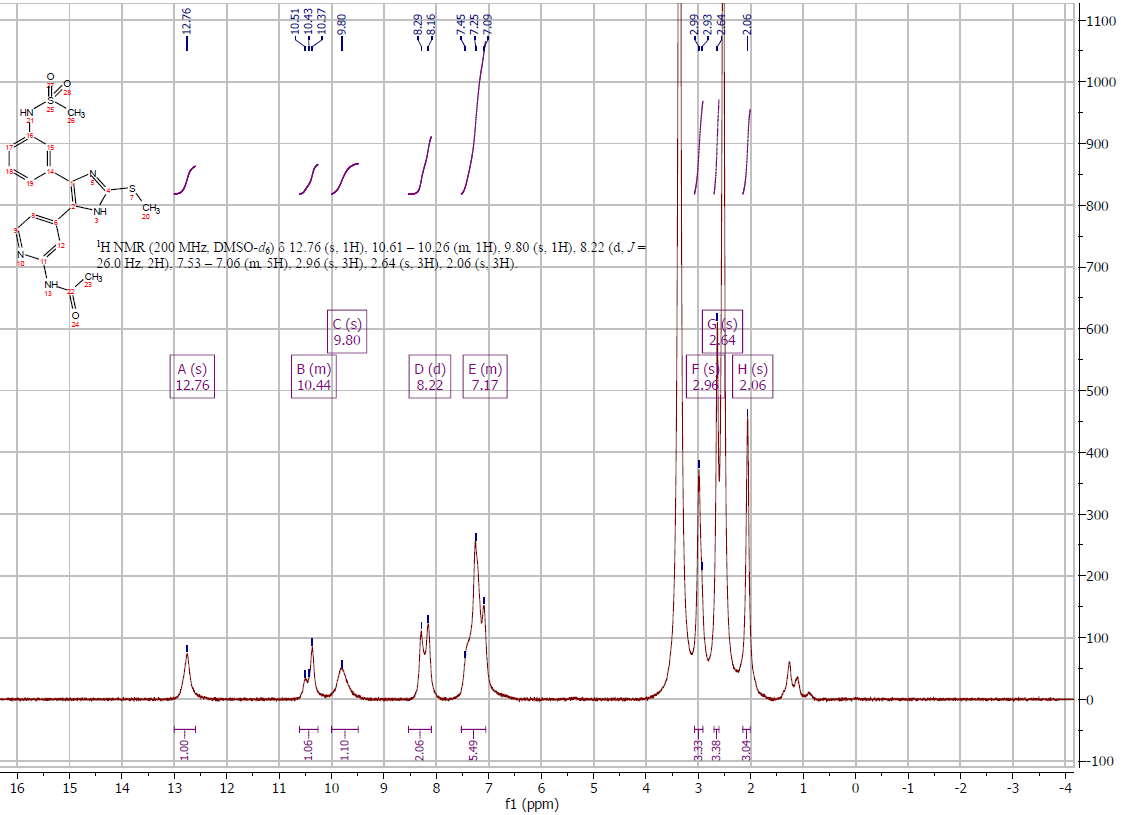


**19**

Compound


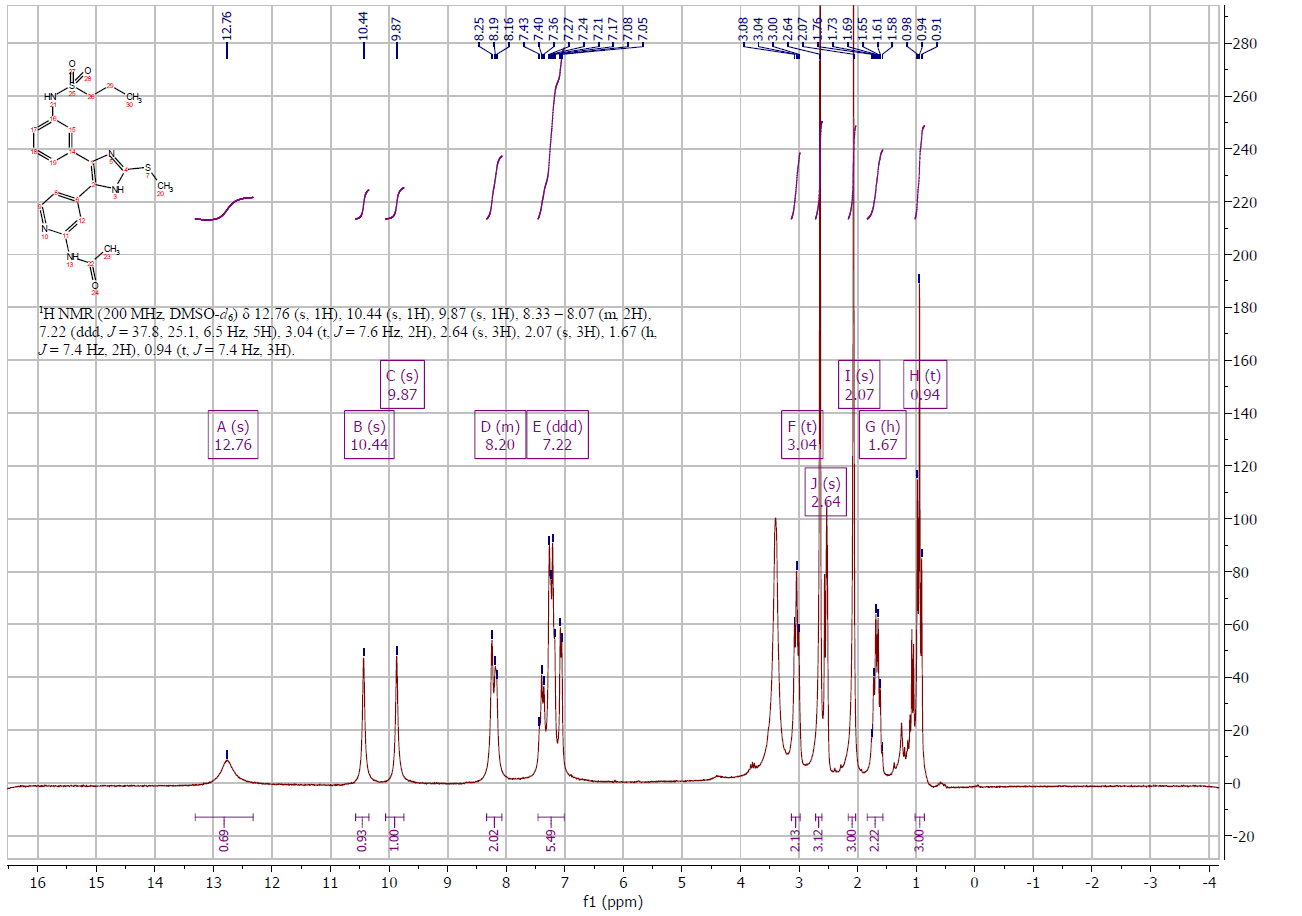


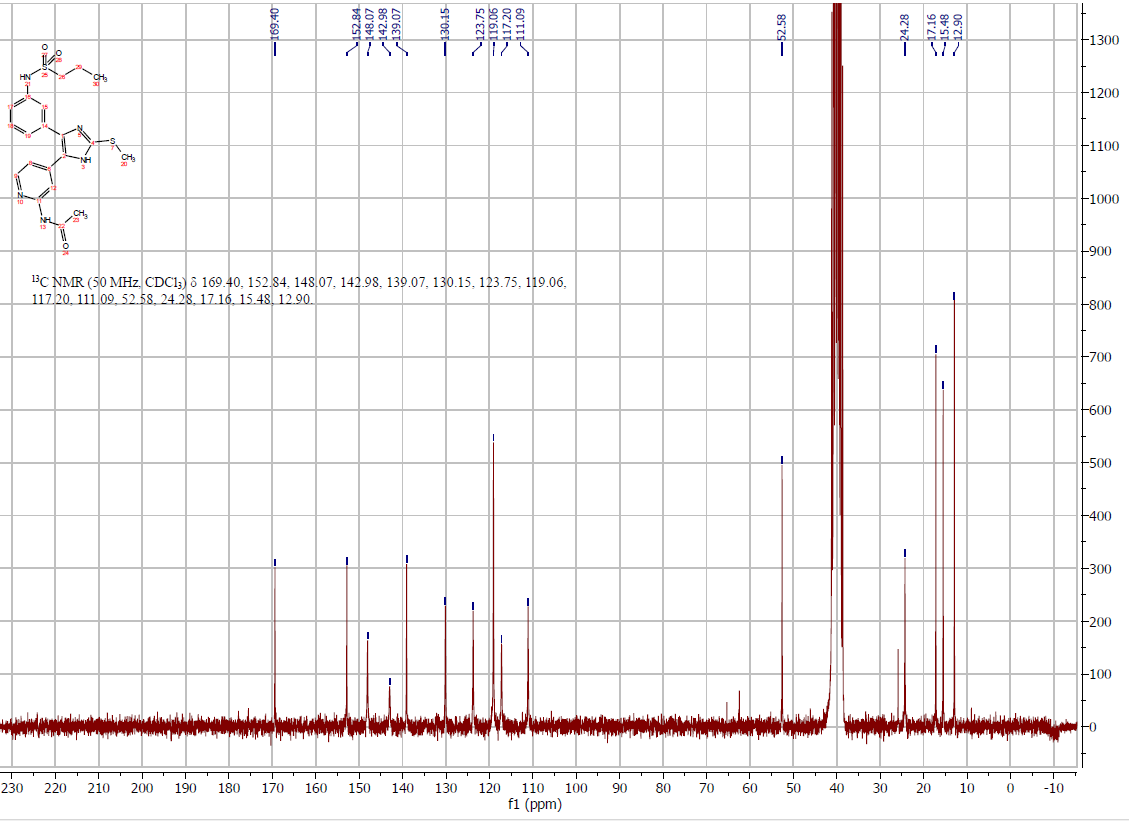


**20**

Compound


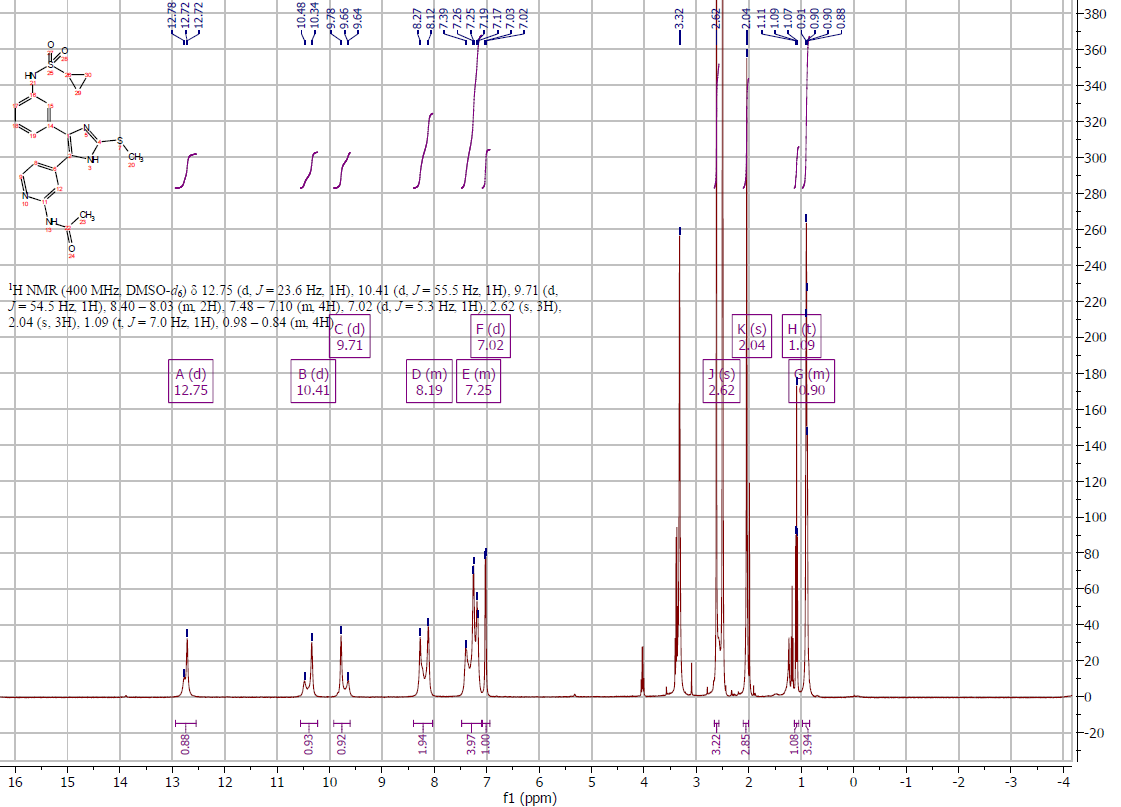


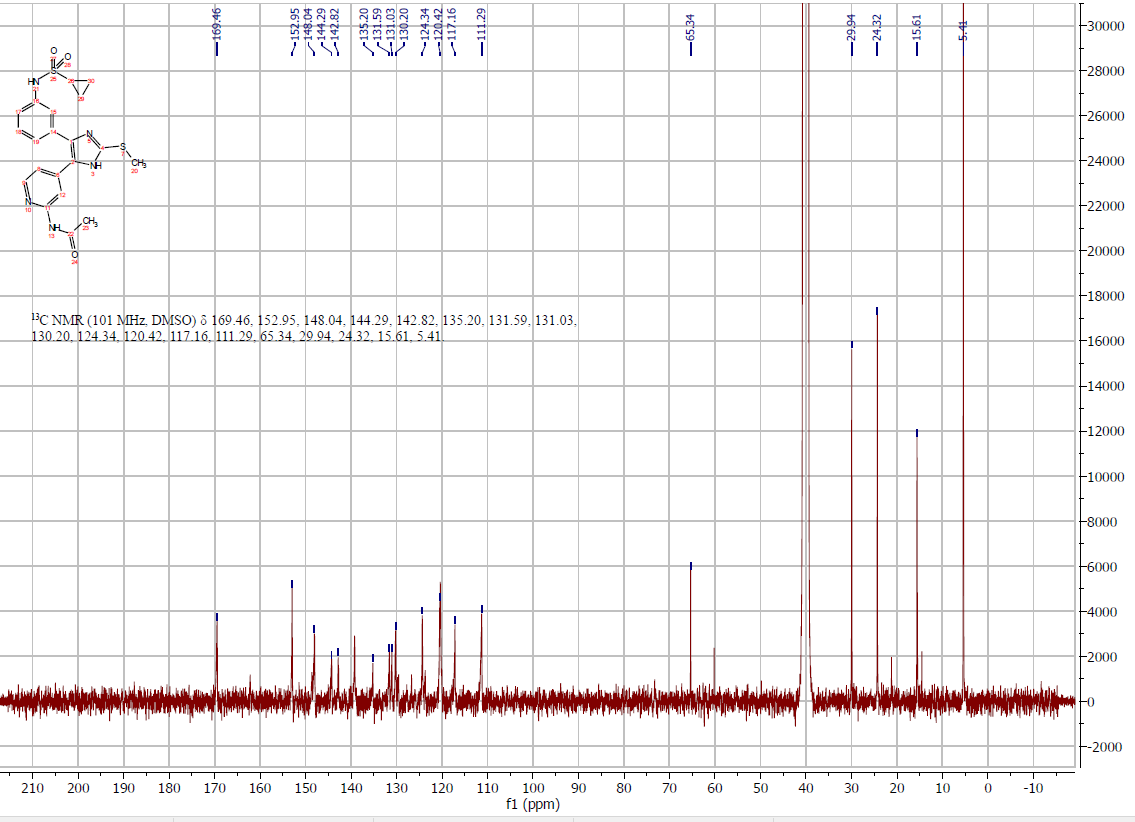


**21**

Compound


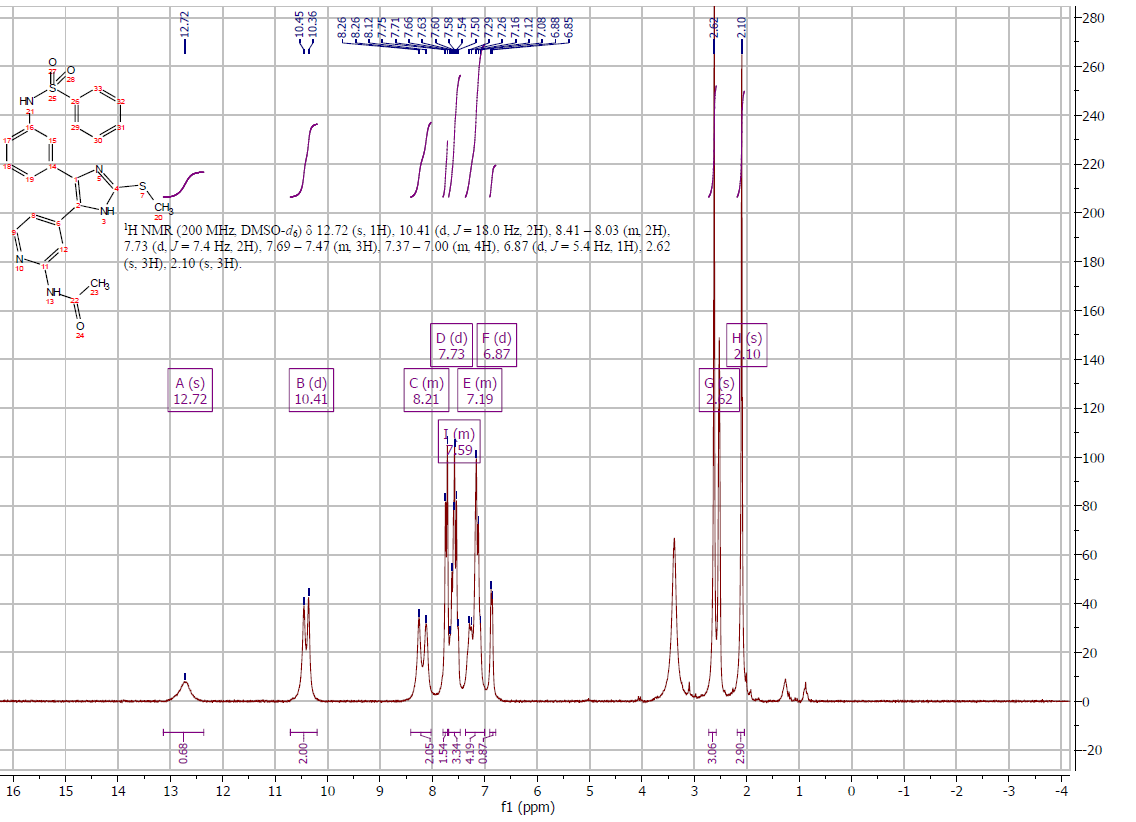


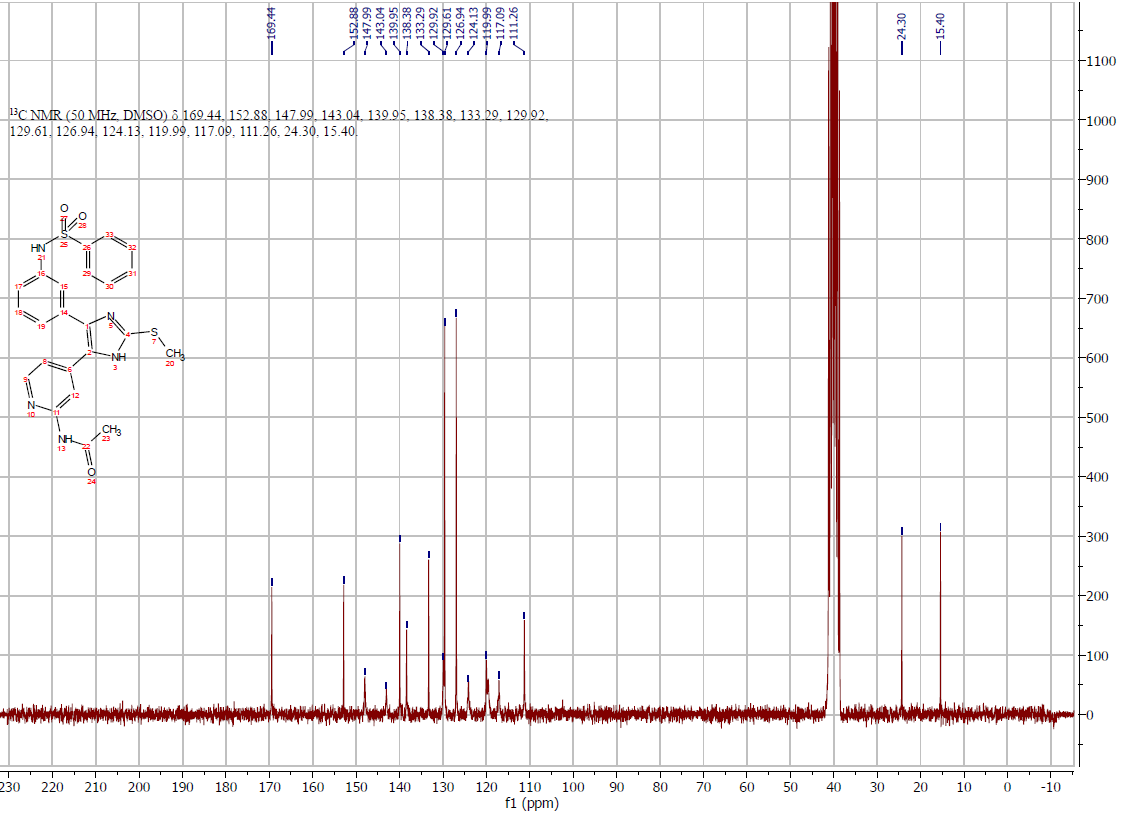


**22**

Compound


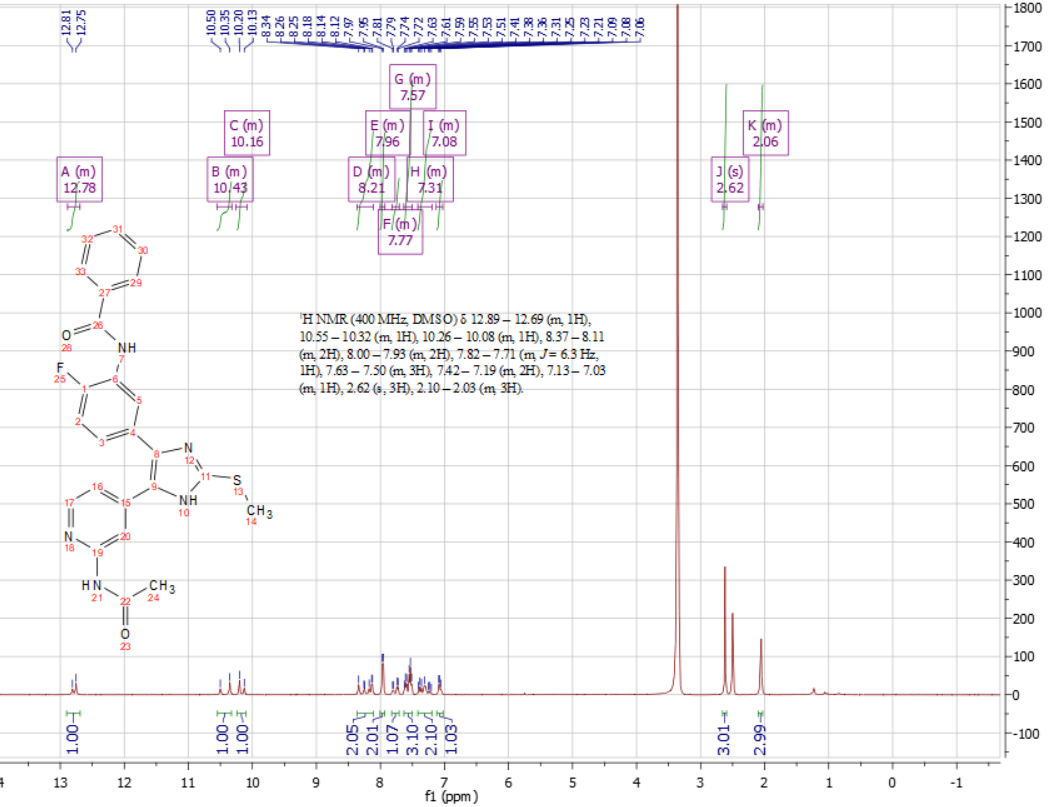


**23**

Compound


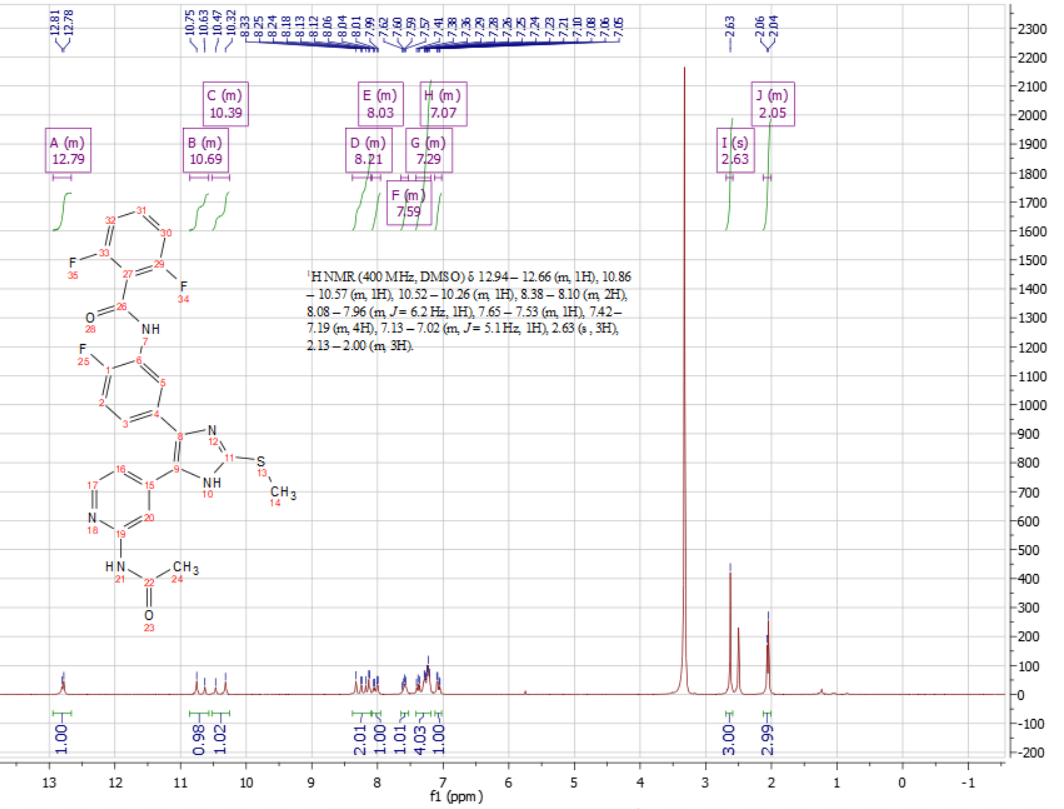


**24**

Compound


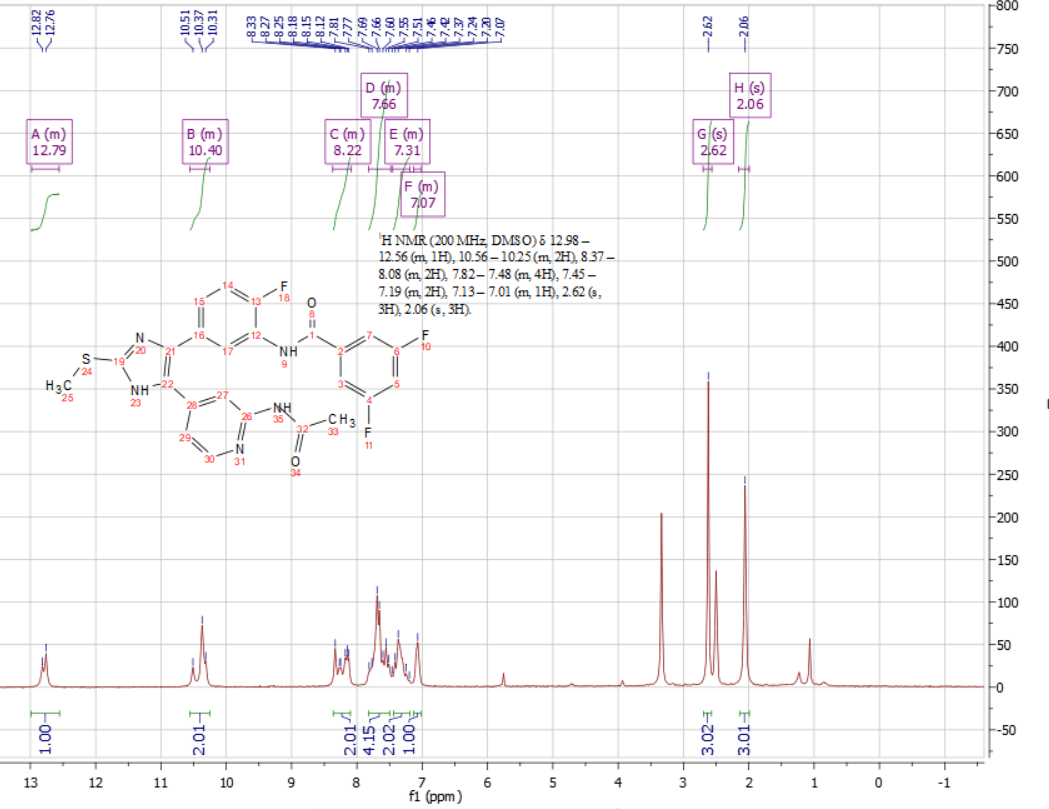


**25**

Compound


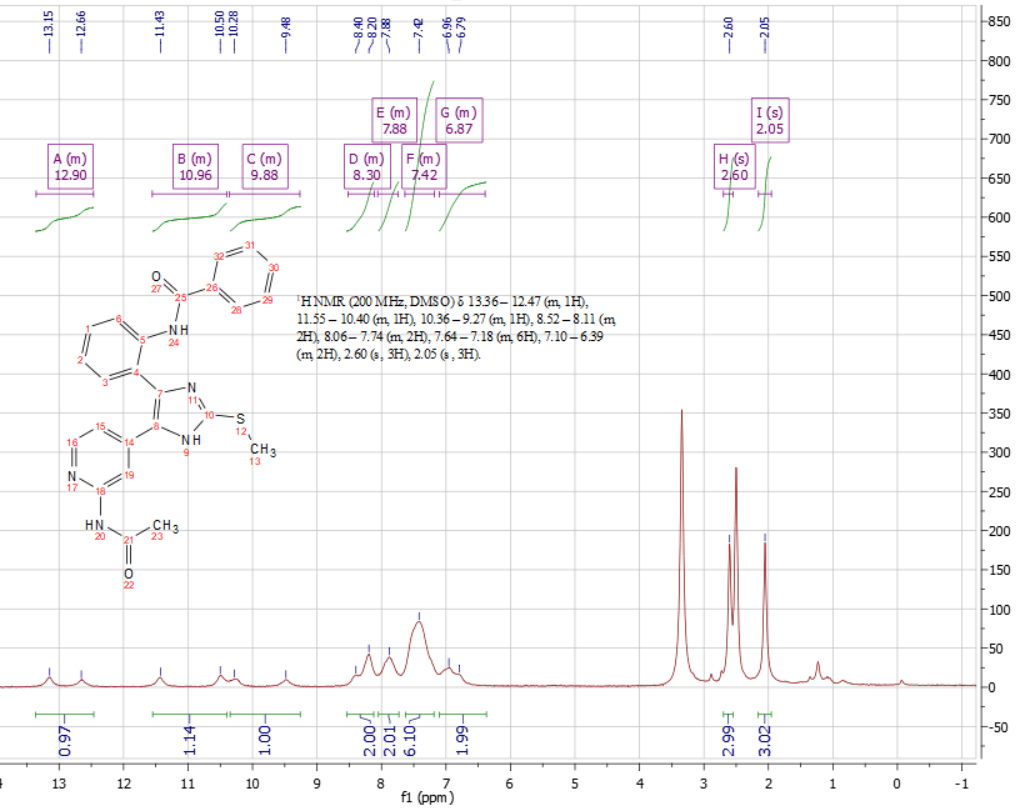


**26**

Intermediate


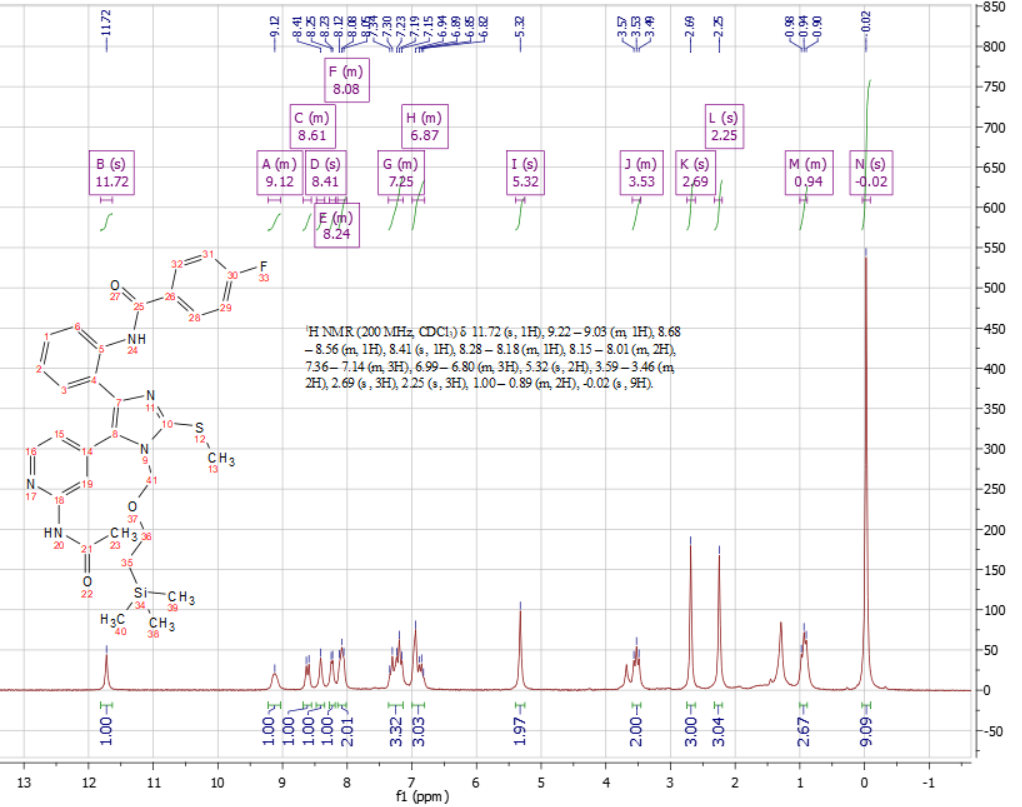


Compound


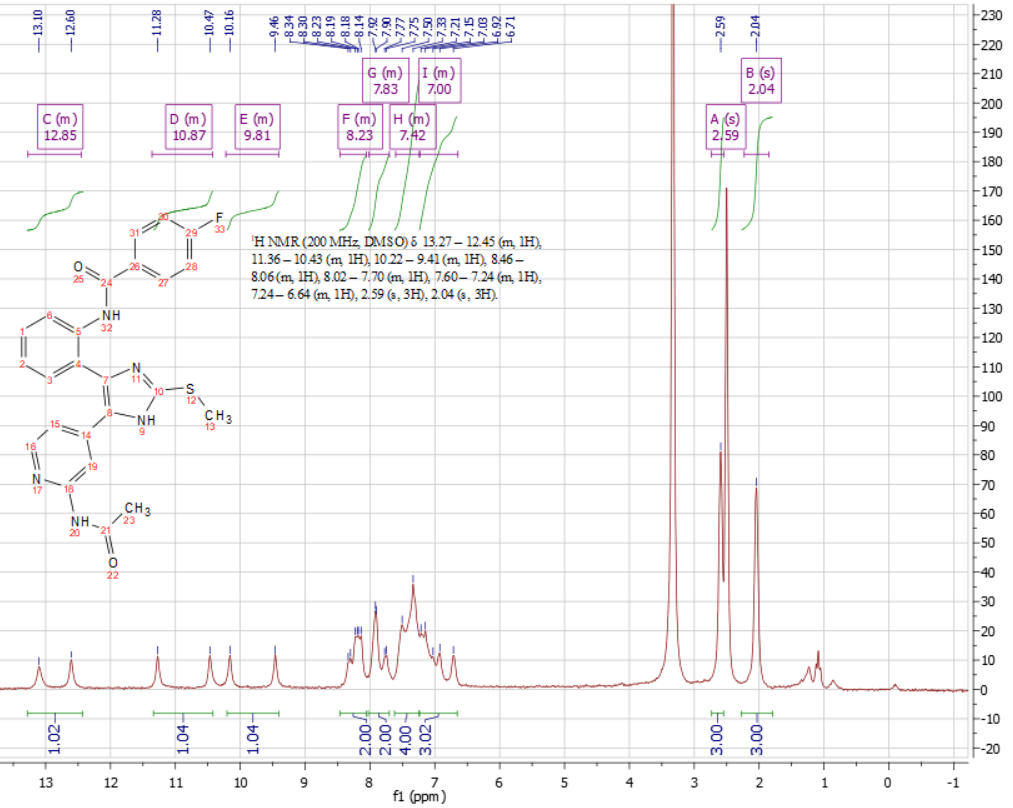


**27**

Intermediate


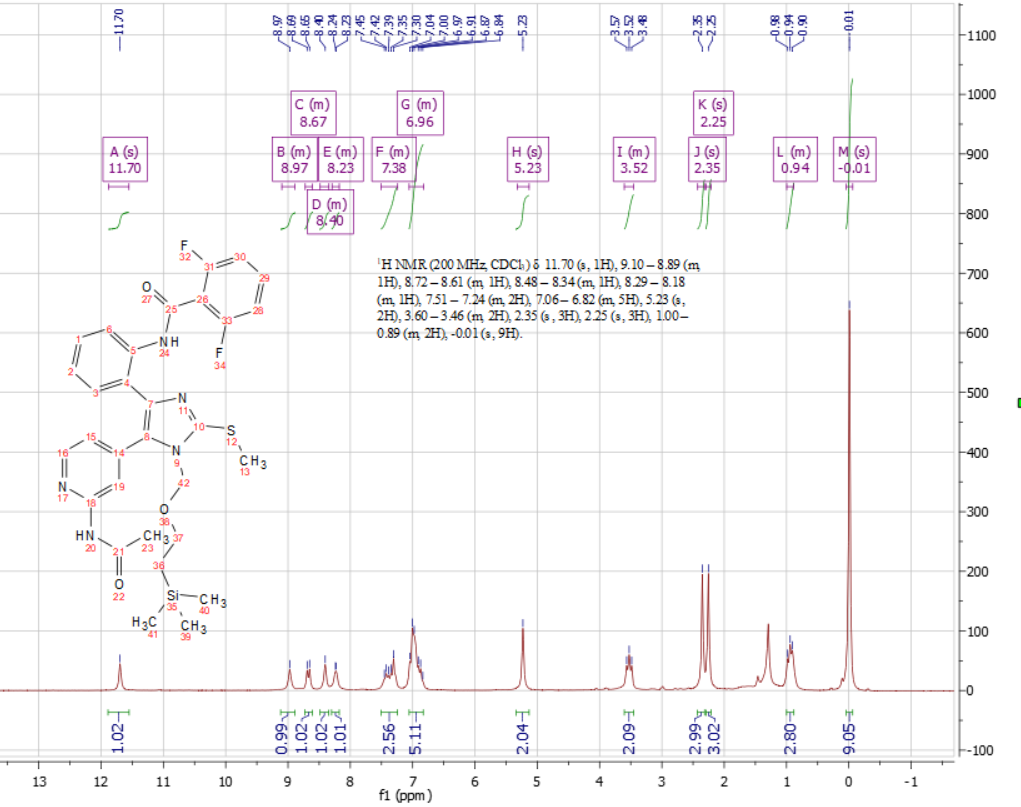


Compound


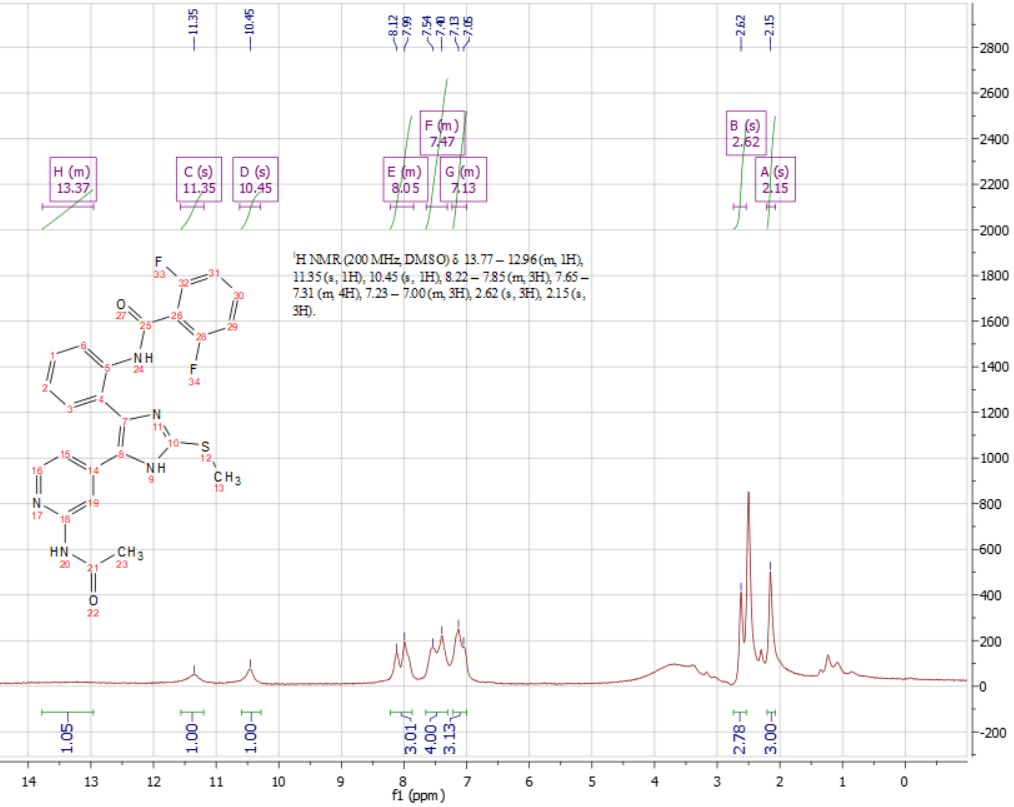


**28**

Intermediate


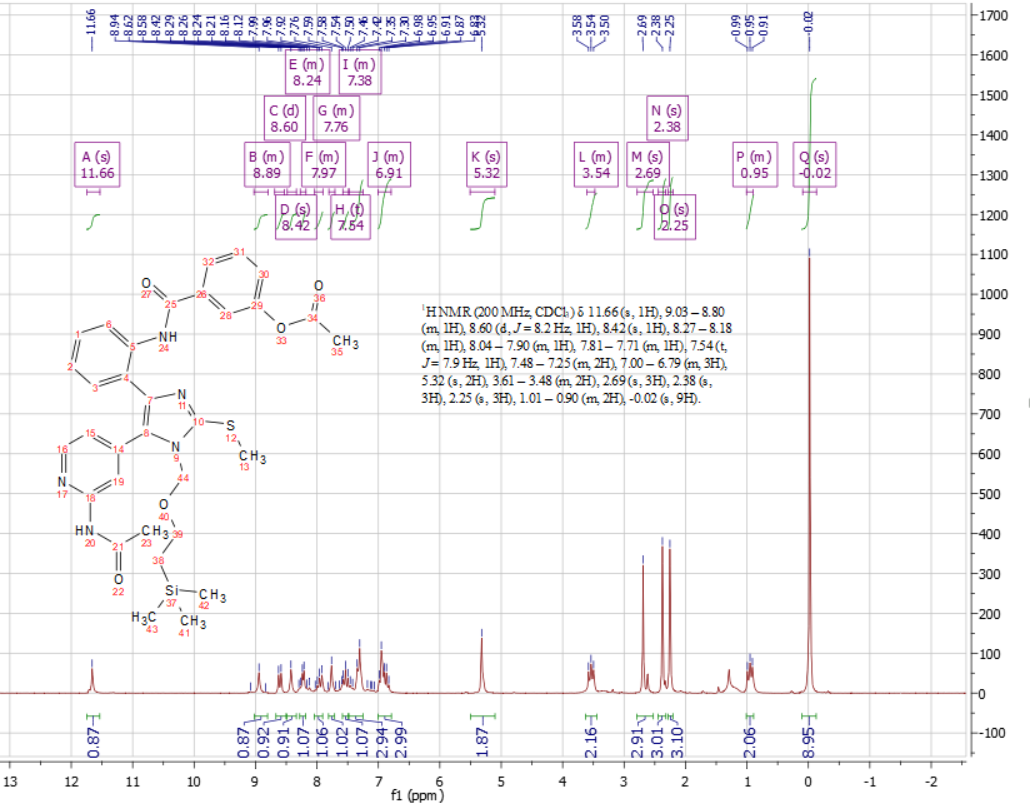


Compound


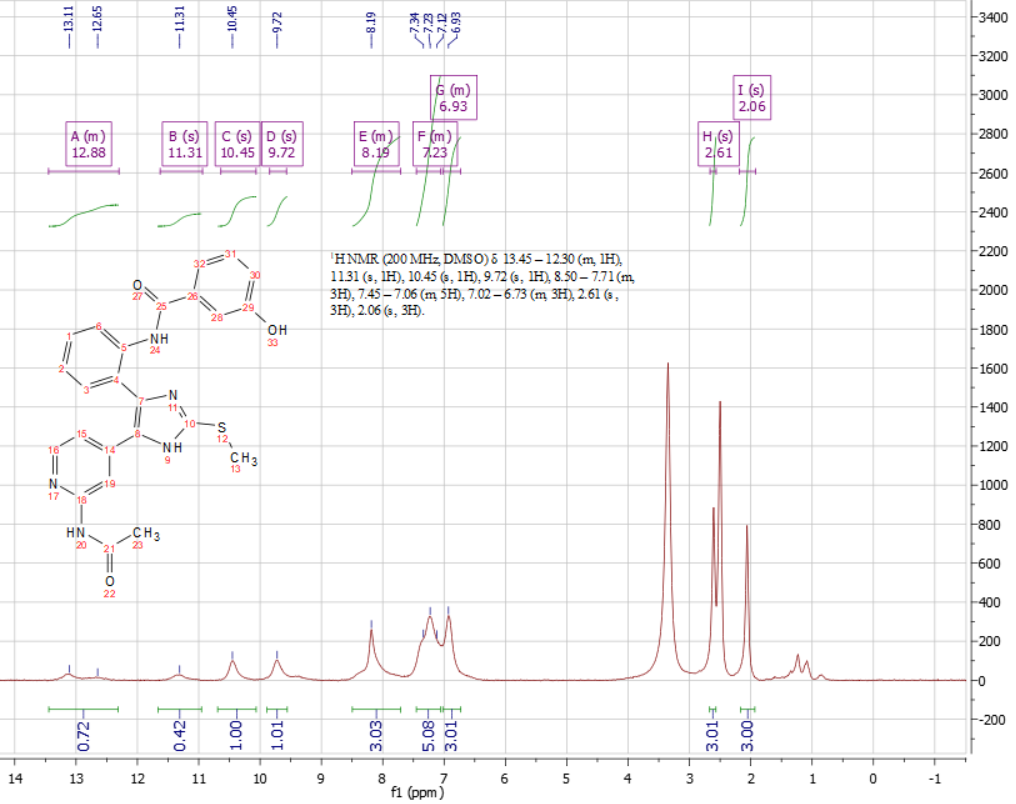


**29**

Intermediate


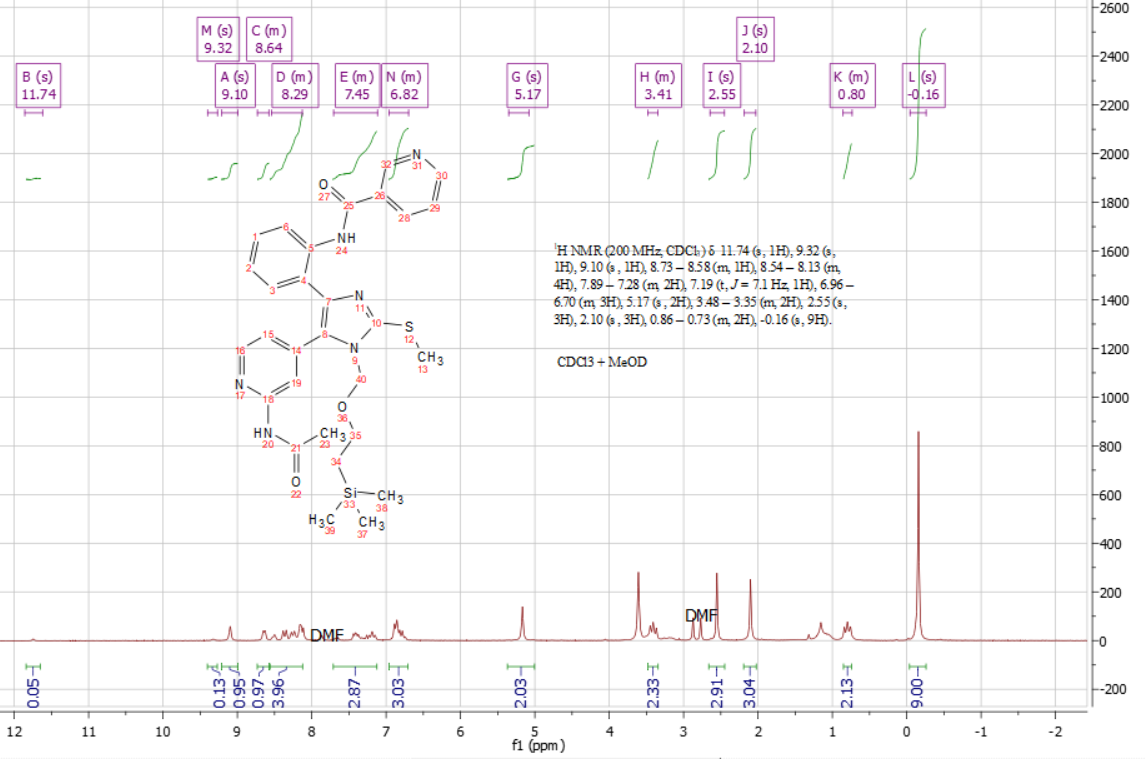


Compound


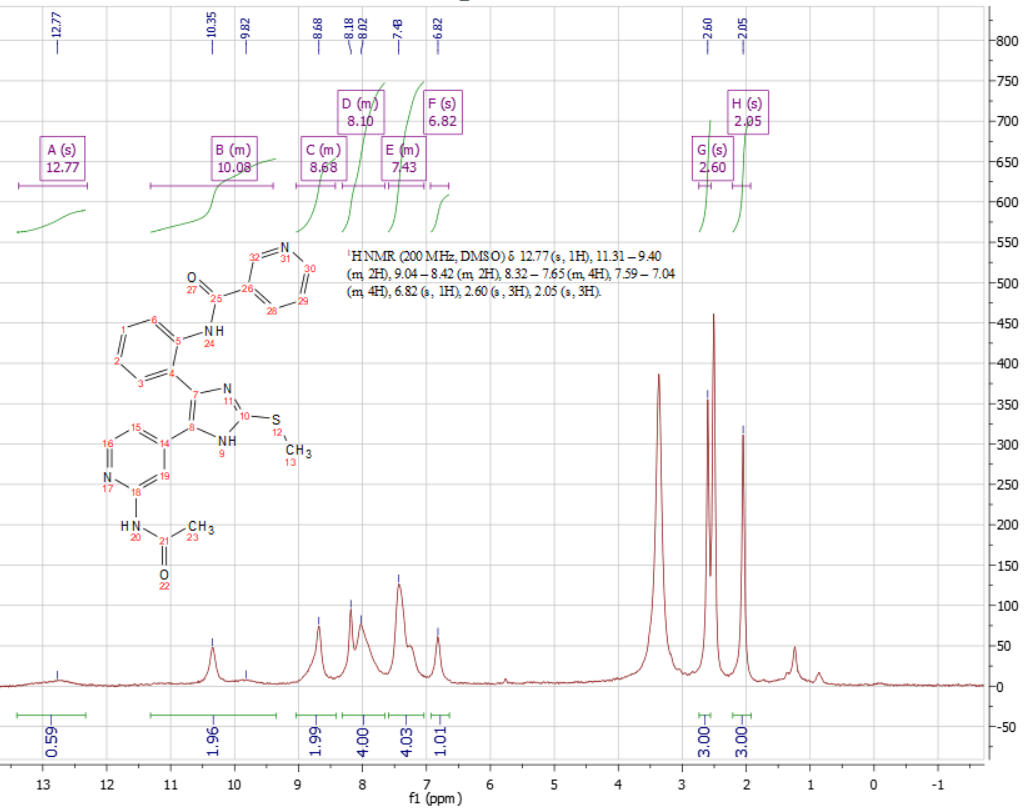


**30**

Intermediate


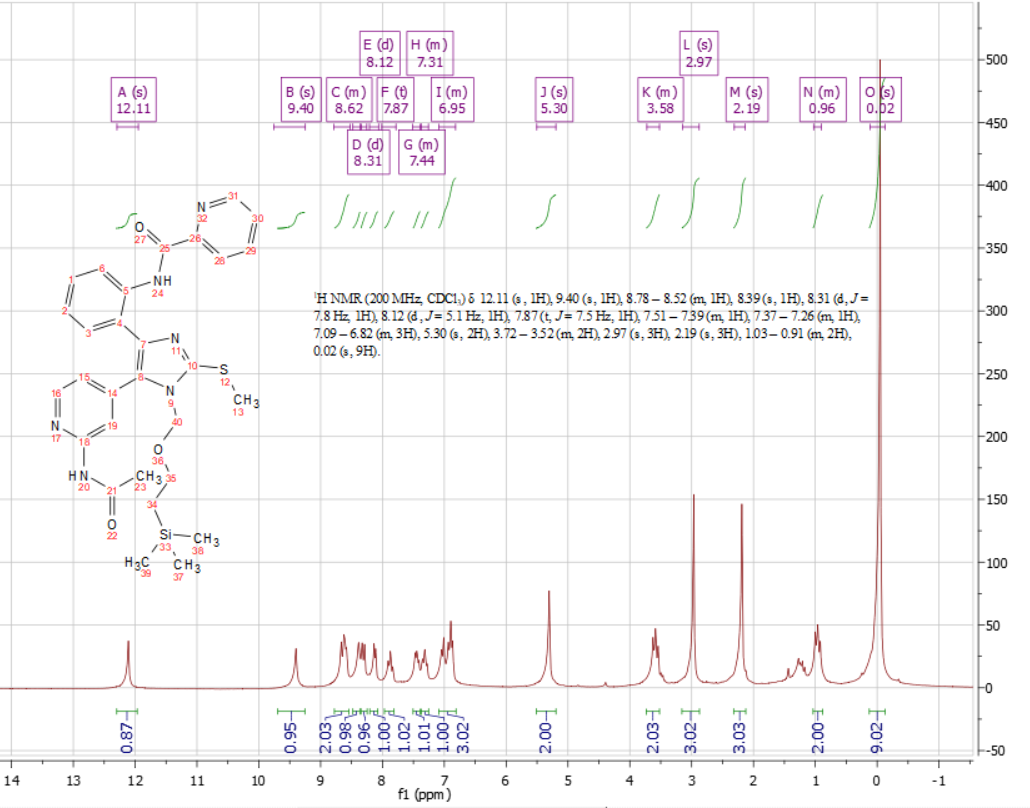


Compound


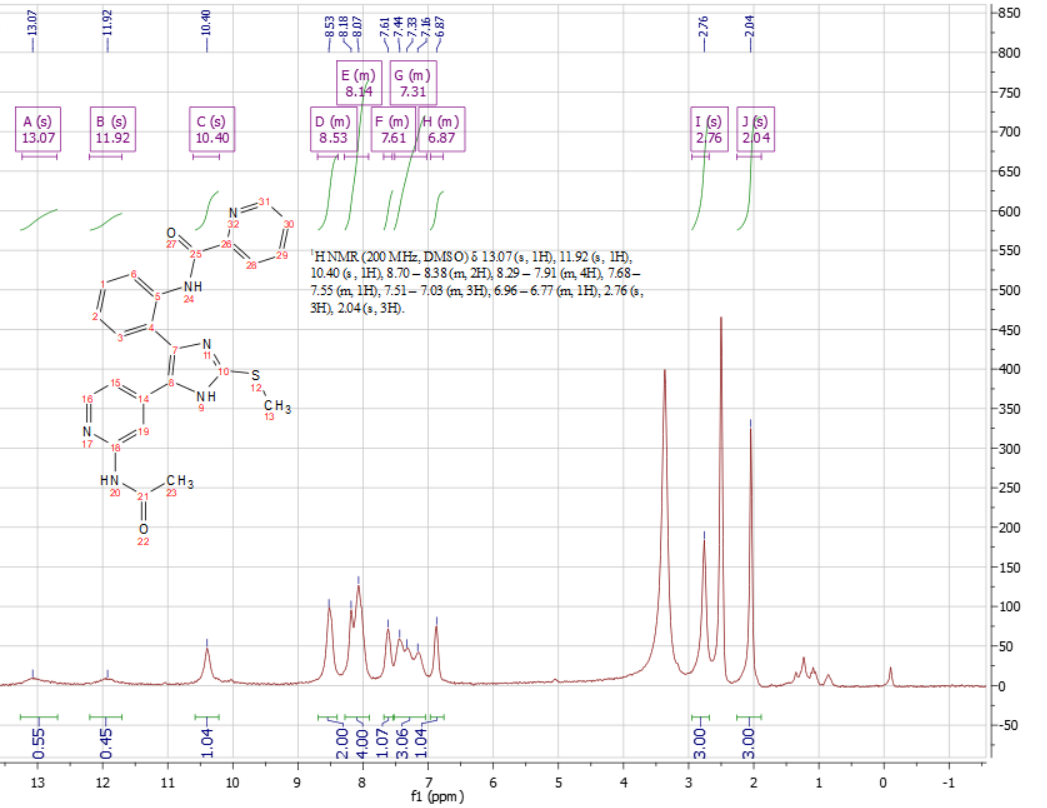


**31**

Intermediate


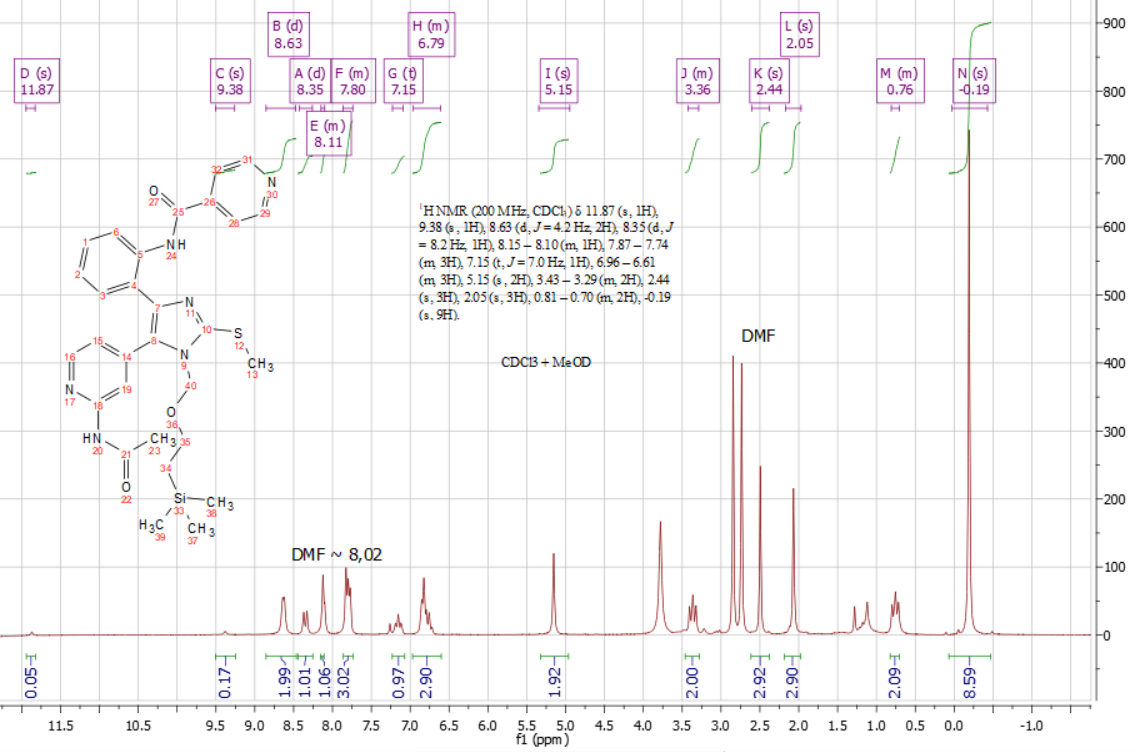


Compound


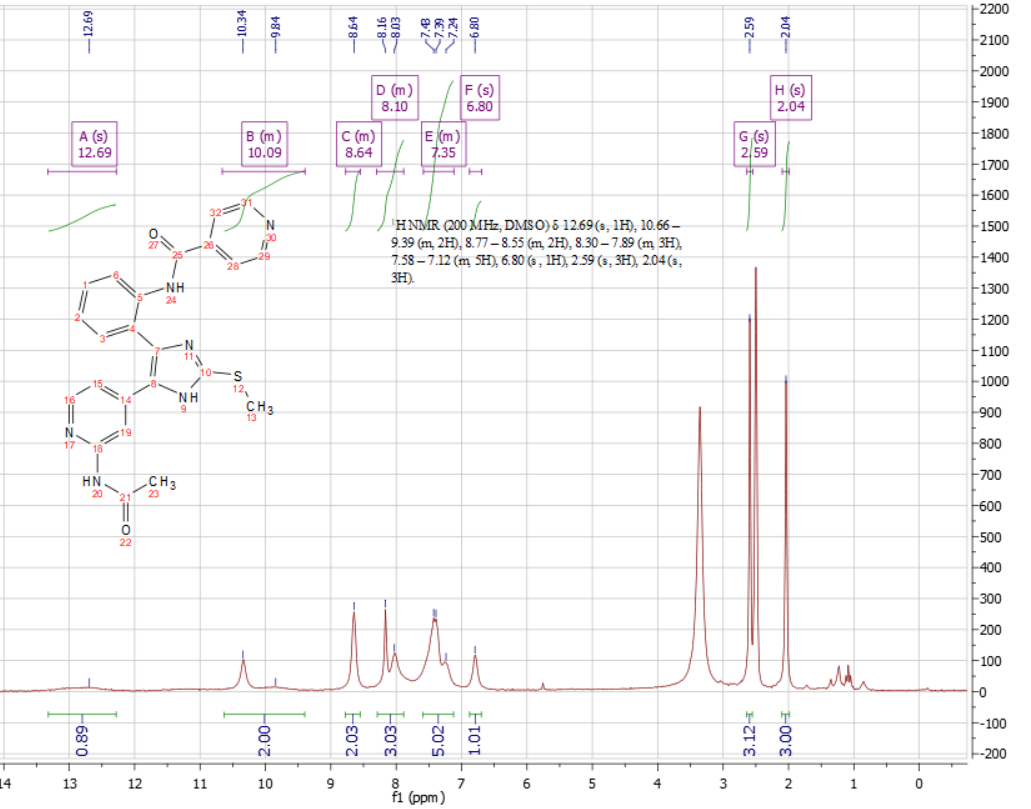

Supplement: Supplementary file 2 — SI SAR at back pocket submit R1 v02. [file ARDP-358-e70027-s002.docx]
